# Acquisition of green algal photobionts enables both chlorolichens and chloro-cyanolichens to activate photosynthesis at low humidity without liquid water

**DOI:** 10.1093/aobpla/plae025

**Published:** 2024-04-29

**Authors:** Fiona Ruth Worthy, Douglas Allen Schaefer, Dhanushka Wanasinghe, Jian Chu Xu, Li Song Wang, Xin Yu Wang

**Affiliations:** Yunnan Key Laboratory for Fungal Diversity and Green Development, Kunming Institute of Botany, Chinese Academy of Sciences, 132 Lanhei Road, Kunming, Yunnan 650201, China; Honghe Centre for Mountain Futures, Kunming Institute of Botany, Chinese Academy of Sciences, 132 Lanhei Road, Kunming, Yunnan 650201, China; Key Laboratory for Plant Diversity and Biogeography of East Asia, Kunming Institute of Botany, Chinese Academy of Sciences, 132 Lanhei Road, Kunming, Yunnan 650201, China; Honghe Centre for Mountain Futures, Kunming Institute of Botany, Chinese Academy of Sciences, 132 Lanhei Road, Kunming, Yunnan 650201, China; Honghe Centre for Mountain Futures, Kunming Institute of Botany, Chinese Academy of Sciences, 132 Lanhei Road, Kunming, Yunnan 650201, China; Department of Soil Science, College of Food and Agriculture Sciences, King Saud University, Riyadh 11362, Saudi Arabia; Honghe Centre for Mountain Futures, Kunming Institute of Botany, Chinese Academy of Sciences, 132 Lanhei Road, Kunming, Yunnan 650201, China; Yunnan Key Laboratory for Fungal Diversity and Green Development, Kunming Institute of Botany, Chinese Academy of Sciences, 132 Lanhei Road, Kunming, Yunnan 650201, China; Key Laboratory for Plant Diversity and Biogeography of East Asia, Kunming Institute of Botany, Chinese Academy of Sciences, 132 Lanhei Road, Kunming, Yunnan 650201, China; Yunnan Key Laboratory for Fungal Diversity and Green Development, Kunming Institute of Botany, Chinese Academy of Sciences, 132 Lanhei Road, Kunming, Yunnan 650201, China; Key Laboratory for Plant Diversity and Biogeography of East Asia, Kunming Institute of Botany, Chinese Academy of Sciences, 132 Lanhei Road, Kunming, Yunnan 650201, China

**Keywords:** humidity, *Lobaria*, molecular phylogeny, mycobiont, *Nostoc*, *Parachloroidium*, photobiont, photosynthesis, *Symbiochloris*

## Abstract

Cyanobacteria require liquid water for photosynthesis, whereas green algae can photosynthesise with water vapour alone. We discovered that several *Lobaria* spp. which normally have cyanobacteria as the sole photobiont, in some regions of the trans-Himalayas also harboured green algae. We tested whether green algal acquisition was: limited to high elevations; obtained from neighbouring chloro-*Lobaria* species; enabled photosynthesis at low humidity. *Lobaria* spp. were collected from 2000 to 4000 m elevation. Spectrophotometry quantified green algal abundance by measuring chlorophyll *b* (absent in cyanobacteria). Thalli cross-sections visually confirmed green algal presence. We sequenced gene regions: *Lobaria* (*ITS-EF-1α-RPB2*), green algae (*18S-RBC-L*) and *Nostoc* (*16S*). Phylogenetic analysis determined myco-photobiont associations. We used a custom closed-circuit gas exchange system with an infrared gas analyser to measure CO_2_ exchange rates for desiccated specimens at 33%, 76%, 86% and 98% humidity. Cross-sections revealed that the photobiont layers in putative cyano*-Lobaria* contained both cyanobacteria and green algae, indicating that they should be considered chloro-cyanolichens. Chloro-*Lobaria* had no visible cephalodia nor cyanobacteria in the photobiont layer. Chloro-*Lobaria* and chloro-cyano-*Lobaria* had comparable levels of chlorophyll *b*. Chloro-*Lobaria* usually contained *Symbiochloris.* Chloro-cyano-*Lobaria* mainly associated with *Parachloroidium* and *Nostoc*; infrequently with *Symbiochloris*, *Apatococcus, Chloroidium*, *Pseudochlorella, Trebouxia*. Sequences from two green algal genera were obtained from within some thalli. Desiccated specimens of every *Lobaria* species could attain net photosynthesis with light exposure and 33% humidity. CO_2_ exchange dynamics over a five-day period differed between species. At all elevations, chloro-cyano-*Lobaria* spp. had abundant green algae in the photobiont layer, but green algal strains mostly differed to those of chloro-*Lobaria* spp. Both chloro-*Lobaria* and chloro-cyano-*Lobaria* were capable of conducting photosynthesis without liquid water. The data strongly suggest that they attained positive net photosynthesis.

## Teaser

Multiple *Lobaria* lichen species, previously described as partnerships between cyanobacteria and fungi (cyanolichens), were discovered to have a third partner: green algae. Such 'chloro-cyanolichens' are rare. They had comparable abundance of green algae to neighbouring *Lobaria* 'chlorolichens' in which cyanobacteria are absent. In a few cases, chloro-cyanolichens shared the green algal genera of neighbouring chlorolichens. But most green algae in chloro-cyanolichens were *Parachloroidium* , whereas *Symbiochloris* was predominant in chlorolichens.

Green algae, but not cyanobacteria, can rehydrate and photosynthesise in humid air, without requiring liquid water. Their green algae enabled the chloro-cyanolichens to photosynthesise without liquid water: a clear advantage in dry conditions. We also detected photosynthesis even while lichens were completely desiccated, in dry air. This expands our understanding of the conditions in which key life processes are possible. Lichens could maintain a positive carbon balance for longer periods than previously thought possible: increasing their contribution to terrestrial carbon cycles.

## Introduction

Lichen associations usually comprise: chlorolichens (mycobiont associated with green algal photobionts), cyanolichens (mycobiont associated with cyanobacteria), and cephalolichens (mycobiont associated with primary green algal photobionts, with cyanobacteria comprising secondary photobionts, present in structures called cephalodia). Cephalodia appear to primarily contribute to nitrogen fixation (Rai *et al.* 1981). Occasionally, photosymbiodemes occur which have adjacent thalli with the same mycobiont but differ in having green algal or cyanobacterial photobionts (Green *et al.* 1993). A fifth association is rarely mentioned; chloro-cyanolichens, in which green algae and cyanobacteria are co-primary photobionts, present within the main body of the thallus, with both contributing to photosynthesis (Henskens *et al.* 2012; Magain and Serusiaux 2014).

Whether chloro-cyanolichens’ possession of co-primary photobionts confers any advantage has yet to be tested. It could theoretically expand the potential duration of photosynthesis in dry habitats. Early studies proved that chlorolichens, but not cyanolichens, could photosynthesise without liquid water (Lange *et al.* 1986). Experiments dividing photosymbiodemes demonstrated that this difference corresponded to having green algae versus cyanobacteria as photobionts (Green *et al.* 1993, 2002) – hereafter ‘chlorobionts’ versus ‘cyanobionts’. Macerating cyanolichen tissue showed differing water requirements were independent of thallus structure (Lange *et al.* 1993). Scanning electron microscopy showed that previously-dried *Trebouxia* green algal cells regain full turgidity with water vapour exposure whereas *Nostoc* cyanobacterial cells cannot regain turgidity unless given liquid water (Budel and Lange 1991).

Cyanolichens are most diverse and abundant in moist and shaded habitats, such as forests (Rikkinen 2015). Cyanolichens can also colonise drier regions, including semi-deserts, arctic tundra (Rikkinen 2015) and Antarctica (Wirtz *et al.* 2003), especially if dew is abundant (Gauslaa 2014). Their higher water holding capacity allows them to prolong the period for which they remain hydrated (Gauslaa and Coxson 2011). However, chlorolichens appear to have a competitive advantage when the main water source is humid air (Gauslaa 2014).

The lowest relative humidity (RH) reported at which chlorolichens have attained positive net photosynthetic rates (NP) was 80% RH for *Ramalina capitata* (Ach.) Nyl. collected from Spain, where RH rarely fell below 80% (Pintado and Sancho 2002). *Ramalina cactacearum* Follmann and *Heterodermia spinulosa* (Kurok.) J.C. Wei, collected from Chilean fog oases, reached positive NP at 82% RH (Lange and Redon 1983). Amongst chlorolichens, humidity thresholds for photosystem II (PSII) activation have been shown to be photobiont-dependent, e.g. 76% for lichens with trentepohlioid, 82% with trebouxioid, and 92% with coccomyxoid algae (Phinney *et al.* 2019).

Recent genetic studies have shown that some lichen associations incorporate more than one type of fungi (Spribille *et al.* 2016; Tuovinen *et al.* 2019), possess multiple chlorobiont species in a single thallus (Paul *et al.* 2018; Blázquez *et al.* 2022), or have the same mycobiont, but differing species of photobionts in different individuals (Singh *et al.* 2017; Blázquez *et al.* 2022). Other mycobionts are highly specific: a single mycobiont always has the same photobiont (Wagner *et al.* 2020). Selectivity can be asymmetrical e.g. generalist photobionts associating with many selective mycobionts (Dal Forno *et al.* 2021; Sanders and Masumoto 2021). For all bionts, selectivity can vary with climate (Singh *et al.* 2017). Such ‘photobiont switching’, ‘stealing’ or ‘sharing’ may occur through mycobionts acquiring the photobionts associated with neighbouring lichens (Cardos *et al.* 2019), either directly from lichen thalli (Holtan-Hartwig and Timdal 1987) or from their substrates (Medeiros *et al.* 2021).

Photobiont diversity has frequently been hypothesised to expand mycobionts’ ecological amplitude (Rolshausen *et al.* 2018; Vančurová *et al.* 2018). Photobionts do appear to vary between soil types (Škvorová *et al.* 2022), continents (Lindgren *et al.* 2020), latitudes (Vančurová *et al.* 2018) and elevation (Devkota *et al.* 2019; Medeiros *et al.* 2021). Nonetheless, supporting experimental evidence for an adaptive advantage to the mycobiont is limited (Škvorová *et al.* 2022).

Photobiont contribution to the lichen association should be associated with their abundance within the thalli. Sanger sequencing should reveal the identity of the most frequent photobiont strain (Paul *et al.* 2018) but cannot determine its absolute abundance. Measuring the photobiont layer in cross-sections (Johansson *et al.* 2011) could quantify abundance. But if co-primary chlorobionts and cyanobionts are suspected, we propose that chlorophyll *b* would be a useful marker. Cyanobacteria do not contain chlorophyll *b* (Tomitani *et al.* 1999). Higher chlorophyll *b* content should reflect more green algal cells, thus extensive acquisition of chlorobionts. This method has not previously been applied to screen cyanobacterial specimens for the presence of green algae.

In the course of a separate experiment (Worthy F.R. unpubl. data) we discovered a *Lobaria* specimen with the morphology of *Lobaria retigera* (Bory) Trevis. (cyanolichen), but with green algal cells visible in cross-sections of the thallus. This specimen was collected from 4000 m elevation, leading us to speculate that chlorobiont acquisition had been an adaptation to harsh high-elevation habitat conditions, and possibly important in extending *L. retigera’*s elevational range. We recognised a unique opportunity to test hypotheses regarding advantages of photobiont switching.

**Figure 1.**
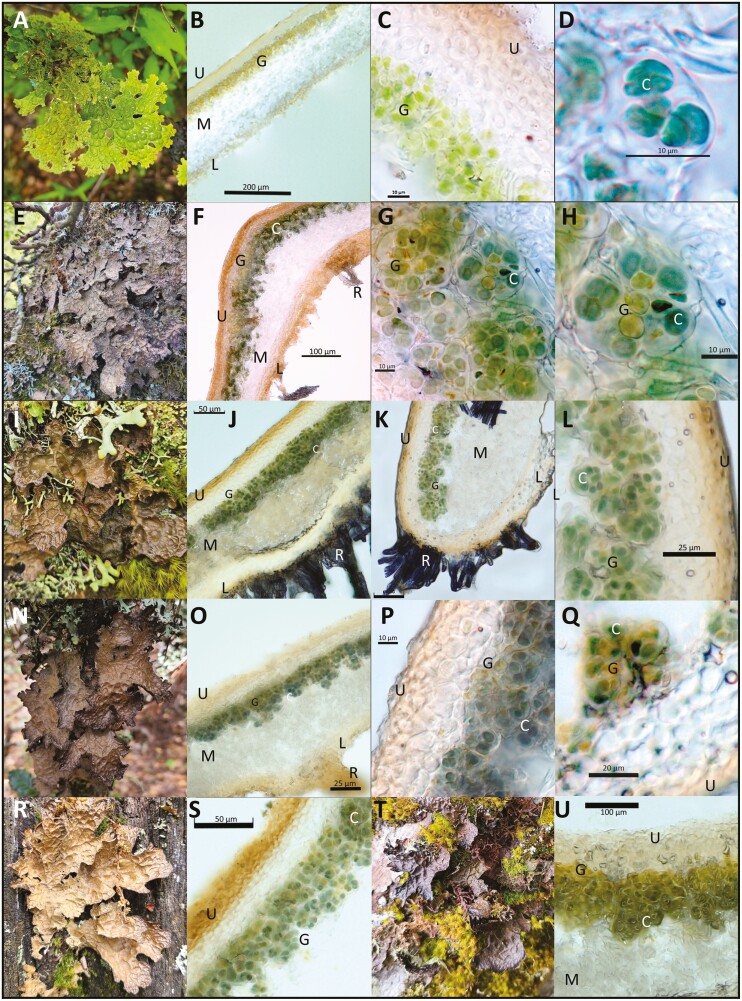
Field photographs and vertical sections through thalli of: (A, B) *Lobaria pindarensis,* (C) *L. costata* (D, T, U)*, L. latilobulata* (E, F, G, H), *L. hengduanensis,* (I, J, K, L), *L. isidiosa* and (N, O, P, Q, R, S) *L. retigera.* Sections of the thallus are labelled as ‘U’ upper cortex, ‘M’ medulla, ‘L’ lower cortex and ‘R’ rhizines. These are not species-specific traits. Within the photobiont layer, ‘G’ marks green algae and ‘C’ marks *Nostoc* cyanobacteria. Green algal gene sequences obtained from these thalli were from *Symbiochloris,* with the exceptions of: (I, J, K, L, T, U) *Parachloroidium* and (Q, R, S) *Pseudochlorella. Nostoc* are not present in plates A, B and C. Slides were hydrated with water.

We therefore collected *Lobaria* specimens down an elevational gradient, with the morphology of chlorolichens (Fig. 1A) and cyanolichens (Fig. 1E, I, N, R, T). Based on the phylogenetic analyses described in this study, the chlorolichens included *Lobaria pindarensis* Räsänen*, Lobaria perelegans* M.X. Yang and Scheid. and *Lobaria costata* M.X. Yang and Scheid. Specimens with the morphology of cyanolichens included *Lobaria retigera, Lobaria isidiosa* (Müll. Arg.) Vain.*, Lobaria latilobulata* C.C. Miao and Li S. Wang*, Lobaria kurokawae* Yoshim., and *Lobaria hengduanensis* C.C. Miao and Li S. Wang.

The specimens were used to test the following, non-mutually exclusive, hypotheses.

1. Chloro-cyano-*Lobaria* spp. have acquired green algal photobionts only at the highest elevations.
2. Chlorophyll *b* content will be greater at higher elevations.
3. Chloro-cyano-*Lobaria* spp. have acquired green algae from neighbouring populations of chloro-*Lobaria*.
4. Acquisition of green algae has been a local process: green algal photobiont species differ between regions.
5. Green algal photobionts enable chloro-cyano-*Lobaria* spp. to photosynthesise without liquid water.
6. Humidity level and duration required to activate photosynthesis will vary with both mycobiont and photobiont identity.

We also recorded local microclimate data, in order to determine the actual temperature and humidity conditions to which the study specimens were exposed in their natural habitat.

## Materials and Methods

Our study sites were in the trans-Himalayas of Yunnan Province, China (Supporting Information 1: Fig. S1). The main sites descended from elevations of 4000 to 3500 m in Diqing Prefecture, and from 3500 to 3300 m in Lijiang Prefecture. This encompassed the full elevational range of *Lobaria* spp. at these sites: from the treeline, down to a complete transition in vegetation type (e.g. terminating at any zone of bamboo or grassland meadow). We collected additional specimens in cloud forests of Honghe Prefecture, from elevations of 2200 to 2100 m, to obtain a comparison group from a geographically separated population, where cyano-*Lobaria* spp. hypothetically would not have acquired chlorobionts.

### Microclimate records

We had recorded microclimate data at the Diqing and Lijiang sites from 2018, prior to this study, until our final specimen collection in late 2020. Travel-constraints caused some gaps in this data-set; therefore, we analysed mean hourly, daily and monthly data for the three years. Extreme winter freezing and heavy summer rainfall sometimes caused logger failure, thus we transitioned from using UniT loggers (UT330) in 2018, to Hygrochron iButton loggers (DS1923) after 2019. These recorded temperature (°C), relative humidity (%) and dewpoint (°C) at half-hourly intervals.

### Specimens used in each component of the study

Lichen specimens collected in 2018 were sectioned and tested for chlorophyll content, leading us to discover the presence of green algae in lichens with the morphology of cyano-*Lobaria*. Consequently, in May and June 2020, we collected *Lobaria* spp. along an elevational gradient in Diqing, Lijiang and Honghe (Supporting Information 1: Fig. S1). Unexpected green algal diversity meant more specimens (collected in Diqing and Lijiang, November 2020) were needed to address hypotheses 4, 5 and 6. All specimens were cross-sectioned, tested for chlorophyll content and photosynthetic response to humidity. However, because lichen chlorophyll content and CO_2_ uptake rates can differ seasonally (Mackenzie *et al.* 2001), while speed and degree of recovery of photosynthetic capacity may progressively decline over prolonged periods of desiccation (Kranner *et al.* 2003; Lüttge 2013), it seemed inadvisable to pool all data for statistical analysis. Therefore, in this paper we analyse chlorophyll data from specimens collected in May and June 2020 and photosynthesis data from specimens collected in November 2020. This meant that not all species are included in all components of the results.

For the chlorophyll data, we present results for chlorolichens *L. costata* (N_s_ = 1 specimen, N_t_ = 3 thallus sub-samples), *L. pindarensis* (N_s_ = 11, N_t_ = 33), *Lobaria* sp. 1 (N_s_ = 2, N_t_ = 6) and other *Lobaria* thalli for which the fungi were not determined (N_s_ = 3, N_t_ = 9). Their green algae were *Symbiochloris* and *Trebouxia*. The chloro-cyanolichens were *L. hengduanensis* (N_s_ = 4, N_t_ = 12), *L. isidiosa* (N_s_ = 3, N_t_ = 9)*, L. kurokawae* (N_s_ = 3, N_t_ = 9)*, L. latilobulata* (N_s_ = 1, N_t_ = 3) *L. retigera* (N_s_ = 4, N_t_ = 16) and other *Lobaria* thalli for which the fungi were not determined (N_s_ = 5, N_t_ = 15). Their green algae were *Parachloroidium, Symbiochloris, Trebouxia* and *Pseudochlorella.*

For the photosynthesis data, we present results for three chlorolichen and three chloro-cyanolichen species. The chlorolichens were *L. pindarensis* (N_s_ = 20)*, L. perelegans* (N_s_ = 1) and *Lobaria* sp. 2. (N_s_ = 5). Their green algae were *Symbiochloris.* The chloro-cyanolichens were *L. isidiosa* (N_s_ = 17)*, L. latilobulata* (N_s_ = 1) and *L. retigera* (N_s_ = 3). Their green algae were *Parachloroidium.* In each case above there were also some green algae for which the genera were not determined. For each specimen there were up to eight records of CO_2_ exchange rates in the dark and 24 records of CO_2_ exchange rates in the light (eight stages × three light conditions).

### Chlorophyll testing

Specimens were air-dried then desiccated over CaCl_2_. Chlorophyll testing was conducted within two weeks of specimen collection. For each specimen, small sections were cut from three different positions on the thalli, within 5 mm of the thallus tip, where the newest, physiologically most active, growth is expected (Majumder *et al.* 2013). Samples of 20 mg were pre-washed with 2 mL CaCO_3_ saturated acetone, three times for 20 min, to remove acidic lichen substances that can otherwise cause chlorophyll to degrade to phaeophytin (Barnes *et al.* 1992). This pre-wash method could be expected to also reduce the abundance of any epibionts on the thallus surface. Samples were kept in the dark between all subsequent steps. Chlorophyll was extracted in 2 mL DMSO, following Ronen and Galun (1984) and Tait and Hik (2003), adapted to obtain the low sample turbidity and volumes required for analysis with a microplate spectrophotometer (Infinite M200 Pro KZ10924, Tecan 2013072-333). Samples were kept for 24 h at 20°C, then 400 µL was extracted and added to 800 µL fresh DMSO. After 30 min, the top 250 µL was extracted and injected into microplates, then refrigerated until insertion into the spectrophotometer. Chlorophyll content (µg per mL DMSO) was calculated using Wellburn (1994) equations for DMSO extraction and 1 nm resolution spectrophotometers, with chlorophyll *a/b*-ratio calculated following Parry *et al.* (2014).

### DNA extraction, amplification and sequencing

Small pieces were removed from the tips of freshly collected thalli, from which any remaining surface material (dust, bark and small thallus fragments) had first been removed by brushing and spraying with water. No green algal epibionts were observed on these thalli. DNA was extracted using a DNA secure Plant Kit (TIANGEN) and PCR products were sequenced using Sanger technology. DNA extraction, PCR amplification and sequencing were conducted by Tsingke Biotechnology Co., Ltd. (Kunming). PCR amplification and programs followed Yang *et al.* (2022). Gene markers and primers were selected based on published research on the phylogeny of *Lobaria* (*ITS*-*RPB2*-*EF-1α*), *Dictyochloropsis* (*18S*-*RBC-L*) and *Nostoc* (*16S*). Primer sequences are provided in Supporting Information 2: Table S1 with accompanying references listed below.

### Phylogenetic analyses

To analyse the sequences generated from different primers of the *ITS*, *EF-1α*, *RPB2*, *18S*, *RBC-L* and *16S* gene regions, we conducted BLAST searches to identify sequences with high similarity indices and find the closest matches with taxa in *Lobaria*, following Miao *et al.* (2018) and Yang *et al.* (2022). Later, we analysed them with other sequences retrieved from GenBank (Supporting Information 2: Table S2). We used MAFFT v. 7 (Katoh *et al*. 2019) to automatically generate multiple alignments of all consensus sequences and reference sequences. We manually corrected alignments using BioEdit v. 7.0.5.2 (Hall 1999) where necessary. We evaluated the single-locus datasets for topological incongruence among the loci concerning members of the analyses by comparing the phylogenetic trees derived from each locus. The resulting alignments were then concatenated into multi-locus alignments and analysed using maximum likelihood (ML) and Bayesian (BI) phylogenetic methods in the CIPRES Science Gateway (Miller *et al*. 2010). We obtained ML trees using RAxML-HPC2 on XSEDE v. 8.2.10 (Stamatakis 2014) with GTR + G + I model and calculated support values with 1000 bootstrap percentage replicates (Felsenstein 1985).

The best-fit models were selected based on Bayesian Information Criterion (BIC) scores using the IQ-TREE web application at http://iqtree.cibiv.univie.ac.at (Trifinopoulos *et al.* 2016). We performed BI with 30 M generations, using four chains in each, and retaining a tree every 1000 generations. The datasets were partitioned according to distinct genetic regions: *ITS1, 5.8S, ITS2, EF-1α* and *RPB2*. For each partition, we employed models recommended by IQ-TREE, selecting them based on their best-fit criteria. The computational analysis was configured to conclude automatically once the standard deviation of split frequencies fell below 0.01, incorporating a burn-in fraction of 25%. Finally, we visualised the phylograms using the FigTree v1.4.0 program (Rambaut 2012) and edited appearance in Microsoft PowerPoint (2016). We followed the above methods to produce separate green algae and *Nostoc* phylograms. Because multiple strains of green algae might be present within some thalli, a combined green algae tree was inappropriate. We therefore present separate *18S* and *RBC-L* trees. Bipartite networks between *Lobaria—*green algae and *Lobaria—Nostoc* were visualised using the Bipartite Network Analysis application (Greenville 2018).

### Microscopy

All specimens were sectioned by hand under a dissecting microscope (Nikon SMZ745), hydrated with water and checked under an optical microscope (Nikon Eclipse E200) for presence of a green algal layer. No green algal epibionts were observed. Photographs were taken with microscopes Olympus DSX1000 and ZEISS scope A1-AX10. Exposure and magnification were controlled with ZEN 3.9 software.

### LICOR

In November 2020, 120 freshly collected *Lobaria* specimens were given liquid water, placed inside sealed chambers, and CO_2_ exchange rates measured via infra-red gas analysis, using a LICOR 830. Water was added in a 1:2 sample mass to volume ratio. All specimens showed high re-saturation respiration immediately after water addition, so hydrated samples were kept dark at the target temperature for 1 h prior to beginning measurements. Dark respiration was recorded for 5 min, then light response was recorded for at least 5 min after specimens reached positive net photosynthetic rate (NP). For a subset of both chlorolichen (primarily *L. pindarensis* from Lijiang) and chloro-cyanolichen specimens (primarily *L. isidiosa* from Diqing), we determined temperature, CO_2_ and light response curves, then used these to select a set of conditions for further experiments which generally enabled specimens to achieve the highest positive NP. These were 15°C, 500 µmol mol^−1^ CO_2_ and 300 µmol m^−2^ s^−1^ light. In case light inhibition might be more problematic for desiccated lichens (Gauslaa *et al.* 2012), for the following experiment we also tested photosynthesis at 170 and 30 µmol m^−2^ s^−1^.

A further set of DNA extractions were performed based on material from the thallus tip of 90 of these specimens. Initial analysis of the resulting gene sequences determined for which specimens there were unambiguous sequences of acceptable quality for phylogenetic analysis. Immediately after the LICOR experiment described above, these specimens had been air dried, then desiccated over CaCl_2_. From December 2020 they were stored in the dark over silica gel until humidity exposure experiments from mid-March to May 2021. During the experiment, ambient relative humidity (RH) varied from 35% to 60%. We standardised experimental humidity conditions by circulating air through lichen chambers, that had first passed over a jar containing saturated solutions of either MgCl_2_, NaCl, KCl or K_2_SO_4_, kept refrigerated at 15°C, to give 33.3%, 75.6%, 85.9% and 97.9% RH respectively (Greenspan 1977). If necessary, a further jar of 1 M NaOH was briefly attached to reduce CO_2_ in the humidity-control chambers. The sequence of light treatments for each specimen was: dark (add humidity-control chamber 5 min, remove humidity control chamber), dark 4 min, light 4 min (or until sufficient data points were obtained to fit a linear regression) for each of the three light conditions, swap humidity chambers. The sequence of humidity conditions was: Day 1: MgCl_2_, NaCl, KCl, continue KCl ≈ 24 h; Day 2: KCl, K_2_SO_4,_ continue K_2_SO_4_ ≈ 24 h; Day 3: K_2_SO_4,_ Day 4: K_2_SO_4_ and Day 5: K_2_SO_4;_ stop.

Diagrams, photographs and additional methodological details for our experimental setup, along with examples of raw LICOR data, can be found in Supporting Information 3 (Figs. S2—S7 and Methods S1—S4). Leaks within equipment can significantly impact experiments measuring gas exchange rates. The measures taken to minimise leaks are detailed in Supporting Information 3 (Figs. S5, S6 and Methods S3).

### Data analysis

We calculated the dark respiration rate (*R*), net photosynthetic rate (NP) and gross photosynthetic rate (GP) (µmol CO_2_ min^−1^ g^−1^) (see Supporting Information 3: Methods S4). While exposed to light:


$$\begin{array}{l} GP=NP+R \end{array}$$ (1)

The additional processes of dark CO_2_ fixation by fungi and cyanobacteria mean that for lichen, such calculations can occasionally produce negative values for GP. As this is not a photosynthetic process, negative values for GP were treated as zero.

Carbon exchange rates for each desiccated specimen exposed to humidity conditions in 2021 (*R*_dry_, NP_dry_, GP_dry_) were also calculated as a percentage of the respiration (*R*_wet_) and photosynthesis (NP_wet_, GP_wet_) of the same specimen when supplied with water in 2020.


$$\begin{array}{l} R\%=\left(R_{dry}/R_{wet}\right)\times 100 \end{array}$$ (2)

$$\begin{array}{l} NP\%=\left({NP}_{dry}/{NP}_{wet}\right)\times 100 \end{array}$$ (3)

$$\begin{array}{l} GP\%=\left({GP}_{dry}/{GP}_{wet}\right)\times 100 \end{array}$$ (4)

Software R 4.1.1 (R Core Team, 2021) within RStudio 1.4.1717 was used to plot graphs and analyse interactions between mycobiont and photobiont identity, chlorophyll, humidity conditions and CO_2_ exchange rates. High variation in CO_2_ exchange rates between specimens makes it difficult to visualise small light responses when the full data set is displayed, therefore we also present figures for representative individual specimens.

Dynamic linear models ‘dynlm’ for time series regression were applied (Zeileis 2019), taking a model substitution approach. Mycobiont and photobiont identity required separate models to avoid generating singularities. The impacts of biont identity and elevation on chlorophyll content showed heteroskedasticity. Therefore, Bartlett’s *K*^2^ test was used to test for significant differences in variance.

## Results

### Microclimate data

The minimum daylight relative humidity (RH) varied between sites from 4.5% to 17.8%. The minimum temperature varied from -7.9°C to -17.7°C. Maximum daylight RH varied between sites from 91.3% to 100%. The maximum temperature varied from 20.9°C to 30.9°C. Sites were most humid during the warmer summer rainy season (Supporting Information 1: Fig. S8), which usually lasts from June to September. There was generally higher minimum RH and lower minimum temperatures at higher elevation sites (Supporting Information 1: Fig. S8A and C). Elevational trends showed seasonal variation and were less clear for maximum values (Supporting Information 1: Fig. S8B and D).

At every elevation, there were many months in which minimum daylight RH was between 30% and 70%. Many sites would only receive RH exceeding 80% during the rainy season (Supporting Information 1: Fig. S8B). During August, most sites would receive 75% to 100% RH during daylight hours (Supporting Information 1: Fig. S9A). However, by December, even overnight RH rarely exceeded 75%. During daylight hours, RH frequently stayed below 50%, but rarely dropped below 25% (Supporting Information 1: Fig. S9B). Thus, an ability to enter photosynthesis between 25% to 75% RH would greatly expand lichens’ photosynthetically active hours during the dry season.

### Phylogenetic data

**Figure 2.**
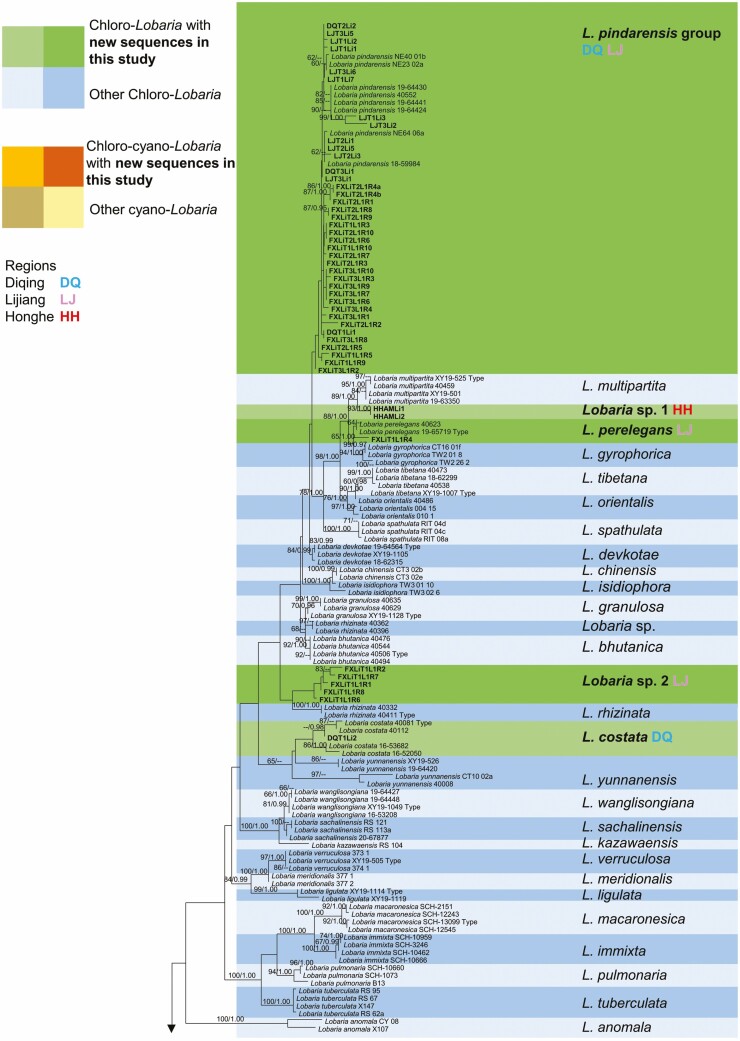

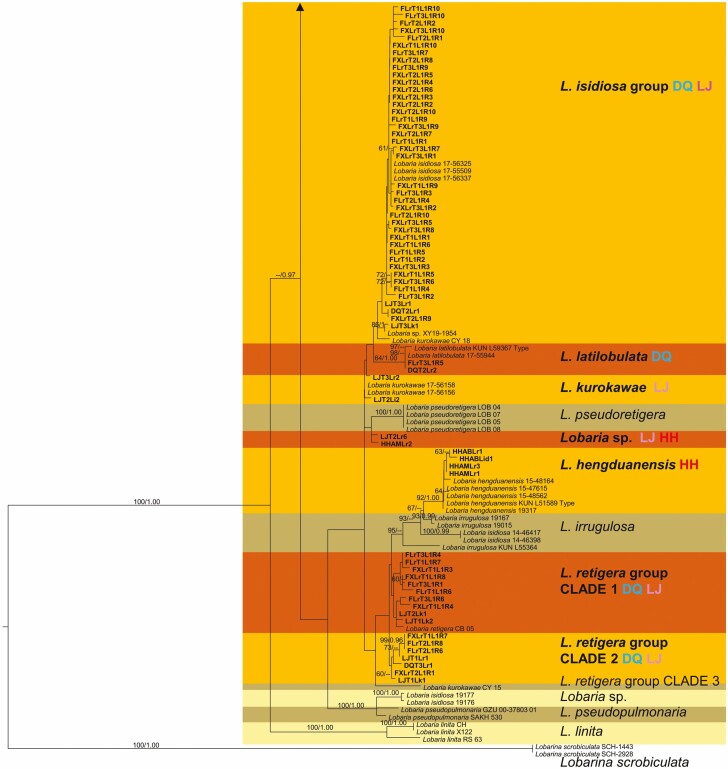
Maximum Likelihood (ML) phylogenetic tree for *Lobaria* based on *ITS*-*EF-1α*-*RPB2.* Nodes with ML bootstrap values ≥ 60% and posterior probabilities in BI (BYPP) ≥ 0.95 are indicated above each node.

Single gene trees for *Lobaria* (not shown) shared their overall topology. However, there was some incongruence because only *ITS* data has been published for several cyano-*Lobaria* species (Supporting Information 2: Table S2). We did not obtain high-quality sequences from all three gene regions for all our specimens. Hereafter we refer to results from the combined tree *ITS*-*EF-1α*-*RPB2* (Fig. 2). A list of all specimens, along with their mycobiont and associated green algae is provided in Supporting Information 2: Table S3.

Unexpectedly, we obtained sequences that differed from any previously published. To describe new species of *Lobaria* lies beyond the scope of this paper. Therefore, for two clades which might potentially comprise new species, we have simply designated them as ‘*Lobaria* sp. 1’ and ‘*Lobaria* sp. 2’. Both these clades are chlorolichens. *Lobaria* sp. 1 is most closely related to *Lobaria multipartita* M.X. Yang and Scheid. *Lobaria* sp. 2 is nearest to *Lobaria rhizinata* M.X. Yang and Scheid, but the clade’s position is poorly supported. Based on our analysis, it appears plausible that the ‘*Lobaria pindarensis* group’ of chlorolichens may comprise multiple distinct species. However, the observed divisions within the dataset fail to constitute well-supported monophyletic clades, thus we treat *L. pindarensis* as a single group. Our other chlorolichen specimens were identified as *L. perelegans* and *L. costata* (Fig. 2).

The majority of our chloro-cyanolichen specimens belonged to the ‘*Lobaria isidiosa* group’. It seems possible that some previous specimens of *Lobaria irrugulosa* C.C. Miao and Li S. Wang (no. 14-46417 and 14-46398) and *Lobaria pseudopulmonaria* Gyeln. (no. 19177 and 19176) have been incorrectly identified as *L. isidiosa.* The ‘*Lobaria retigera* group’ encompasses at least three distinct species, which we divided into clades 1, 2 and 3. Clade 1 contains the only specimen of *L. retigera* for which *ITS*, *EF-1α* and *RPB2* sequences are all published. Clade 2 might be a new species. The correct identification of *L. kurokawae* is uncertain, with possible placement either in *L. retigera* clade 3, in the *L. isidiosa* group, or next to *L. latilobulata* (marked in Fig. 2). We sequenced two specimens of *L. latilobulata* and four of *L. hengduanensis* (Fig. 2). There is poor support for the position of two sequences neighbouring *Lobaria pseudoretigera* Sipman.

*Lobaria* spp. differed in their distribution. *L. costata* and *L. latilobulata* were only found in Diqing. *L. pindarensis* clade 2, *L*. *perelegans, Lobaria* sp. 2 and *L. kurokawae* were only found in Lijiang. *Lobaria* sp. 1 and *L. hengduanensis* were only found in Honghe. Six clades were found in two regions. No clade occurred at all three regions, and none occurred in both the highest and lowest region. Two species were represented by only a single specimen each (Fig. 2).

**Figure 3.**
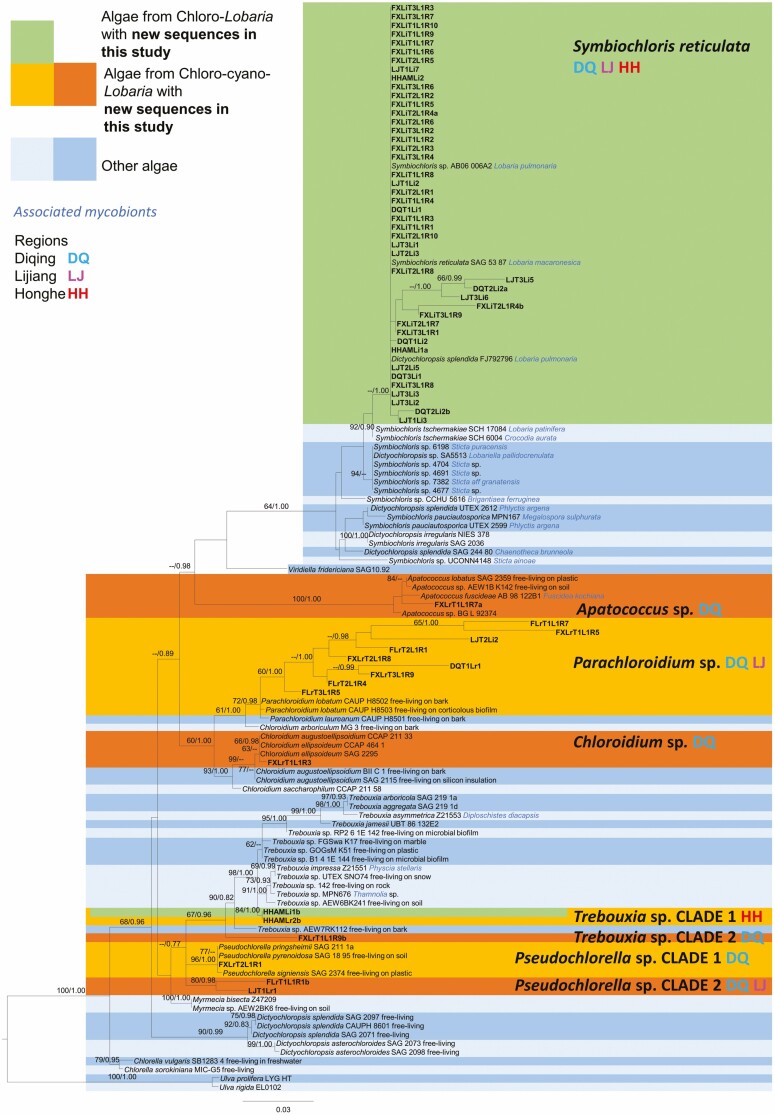
Maximum Likelihood (ML) phylogenetic tree for green algae based on *18S*. Nodes with ML bootstrap values ≥ 60% and posterior probabilities in BI (BYPP) ≥ 0.95 are indicated above each node. If green algae were obtained from lichen thalli, the mycobiont is noted (blue italic font). For free-living green algae, their substrate is stated if known.

**Figure 4.**
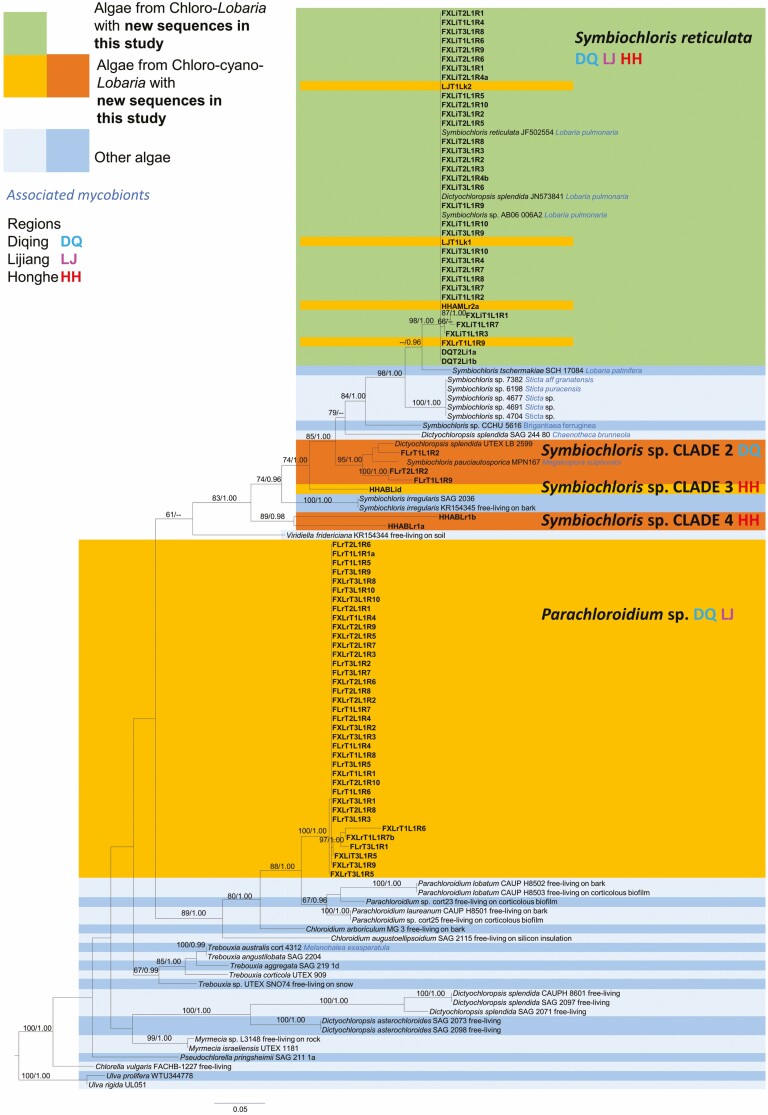
Maximum likelihood (ML) phylogenetic tree for green algae based on *RBC-L*. Nodes with ML bootstrap values ≥ 60% and posterior probabilities in BI (BYPP) ≥ 0.95 are indicated above each node. If green algae were obtained from lichen thalli, the mycobiont is noted (blue italic font). For free-living green algae, their substrate is stated if known.

For many specimens with *Symbiochloris* both *18S* and *RBC-L* regions could be sequenced. *Parachloroidium* sequences were primarily obtained with *RBC-L* primers (Figs. 3, 4). Both *18S* and *RBC-L* sequences were obtained from 38 specimens. Of these, four chloro-cyano-*Lobaria* possessed two green algal genera within a single thallus: *Apatococcus* with *Parachloroidium* from *L. retigera* clade 2; *Pseudochlorella* with *Parachloroidium* from *L. isidiosa*; *Trebouxia* with *Symbiochloris* from both *L. isidiosa* and *L. kurokawae*. For multiple samples sequenced from a single specimen, both *Trebouxia* and *Symbiochloris* were obtained from chlorolichen *Lobaria* sp. 1 (Figs. 3 and 4). Otherwise, green algae from chloro-*Lobaria* were solely *Symbiochloris reticulata* (Tschermak-Woess) Skaloud *et al.* (Figs. 3 and 4). *Dictyochloropsis splendida* Geitler would be an alternative identification (Figs. 3 and 4), but as *D. splendida* is a paraphyletic group, the identification of *S. reticulata* was preferred.

In five instances chloro-cyano-*Lobaria* had the same chlorobiont clades as their neighbouring chloro-*Lobaria. L. isidiosa, L. retigera* (Diqing) and *L. kurokawae* (Honghe) had acquired *S. reticulata.* In Honghe, *Lobaria* sp. 1 and a chloro-cyanolichen *Lobaria* sp. shared the same strains of both *Trebouxia* and *S. reticulata* (Figs. 2–4).

Otherwise, chloro-cyano-*Lobaria* chlorobionts were apparently acquired elsewhere. *Symbiochloris* clades 2, 3 and 4 in chloro-cyano-*Lobaria* were clearly distinct from *Symbiochloris* in chloro-*Lobaria* (Fig. 4). Furthermore, *Parachloroidium* was present in 74.5% of chloro-cyano-*Lobaria,* but never found in chloro-*Lobaria.* These sequences probably represent new species of *Parachloroidium.*

The identity of chlorobionts acquired by chloro-cyano-*Lobaria* showed regional differences. *Trebouxia* clade 1 and *Symbiochloris* clades 3 and 4 were only found in Honghe. *Apatococcus, Chloroidium, Trebouxia* clade 2 and *Pseudochlorella* clade 1 were each found only from a single specimen in Diqing.

All cyanobionts were *Nostoc* strains closely related to *Nostoc commune* Vaucher ex Bornet and Flahault (Supporting Information 1: Fig. S10). These were divided into strains based on their separation between monophyletic sister clades on the constructed phylogenetic tree (Supporting Information 1: Fig. S10). There was some geographic separation: *Nostoc* 7 was only found in Diqing, *Nostoc* 2 was only found in Lijiang and *Nostoc* 6 was only found in Honghe. *Nostoc* 3, 4 and 5 were found in both Diqing and Lijiang. *Nostoc* 1 was found in both Lijiang and Honghe. Three strains have been recorded in other regions, associated with both *Lobaria,* and five other genera. *Nostoc* 1 was recorded in *Pannaria* spp. and *Nephroma resupinatum* (L.) Ach.; *Nostoc* 2 was recorded in *Fuscopannaria leucosticta* (Tuck. ex E. Michener) P.M. Jørg.; *Nostoc* 5 was recorded in *Nephroma* spp., *Parmeliella triptophylla* (Ach.) Müll. Arg. and *Lobaria pulmonaria* (L.) Hoffm. (Supporting Information 1: Fig. S10).

**Figure 5.**
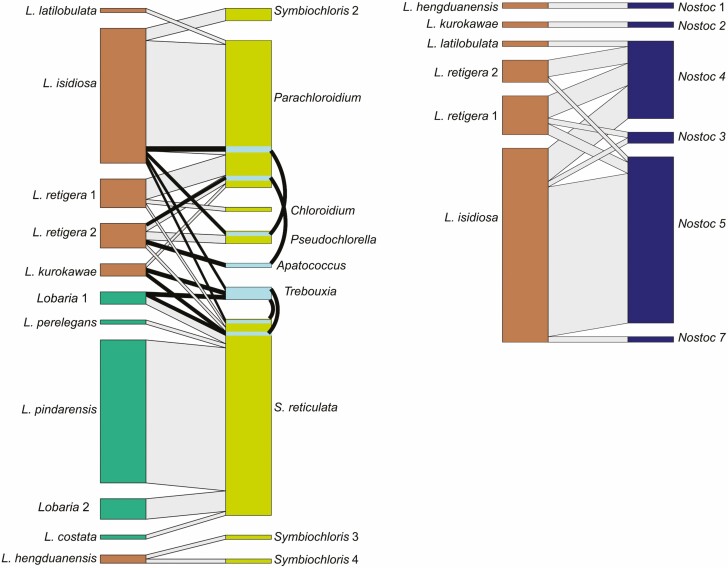
Bipartite networks between new *Lobaria* specimens from this study and their green algae or *Nostoc* photobionts. The width of rectangles is proportional to the number of specimens of chloro-cyano-*Lobaria* (brown), chloro-*Lobaria* (dark green), *Nostoc* (blue) and either a single green algal genus (bright green) or two green algal genera sequenced from a single thallus (turquoise). Links in grey indicate associations between *Lobaria* clades and photobiont clades. Links in black indicate associations with more than one green algal genus sequenced from a single thallus.

Bipartite networks (Fig. 5) showed that whereas *L. latilobulata, L. perelegans, L. pindarensis, L. costata* and *Lobaria* sp. 2 each always associated with the same chlorobiont genus, for others (*L. isidiosa, L. retigera, L. kurokawae, L. hengduanensis* and *Lobaria* sp. 1) different specimens of the same mycobiont were associated with different chlorobionts. Chloro-*Lobaria* spp. were usually associated with *S. reticulata*. Chloro-cyano-*Lobaria* were chiefly associated with *Parachloroidium* spp. Common green algal genera were associated with multiple mycobiont clades, with nine *Lobaria* clades associated with *S. reticulata* and five associated with *Parachloroidium*.

All *Lobaria* spp. were associated with closely related *Nostoc* strains. *Nostoc* strains 1, 2 and 7 were each associated with only a single mycobiont clade. *Nostoc* strains 3, 4 and 5 were associated with multiple mycobiont clades (Fig. 5).

### Chlorophyll content

**Figure 6.**
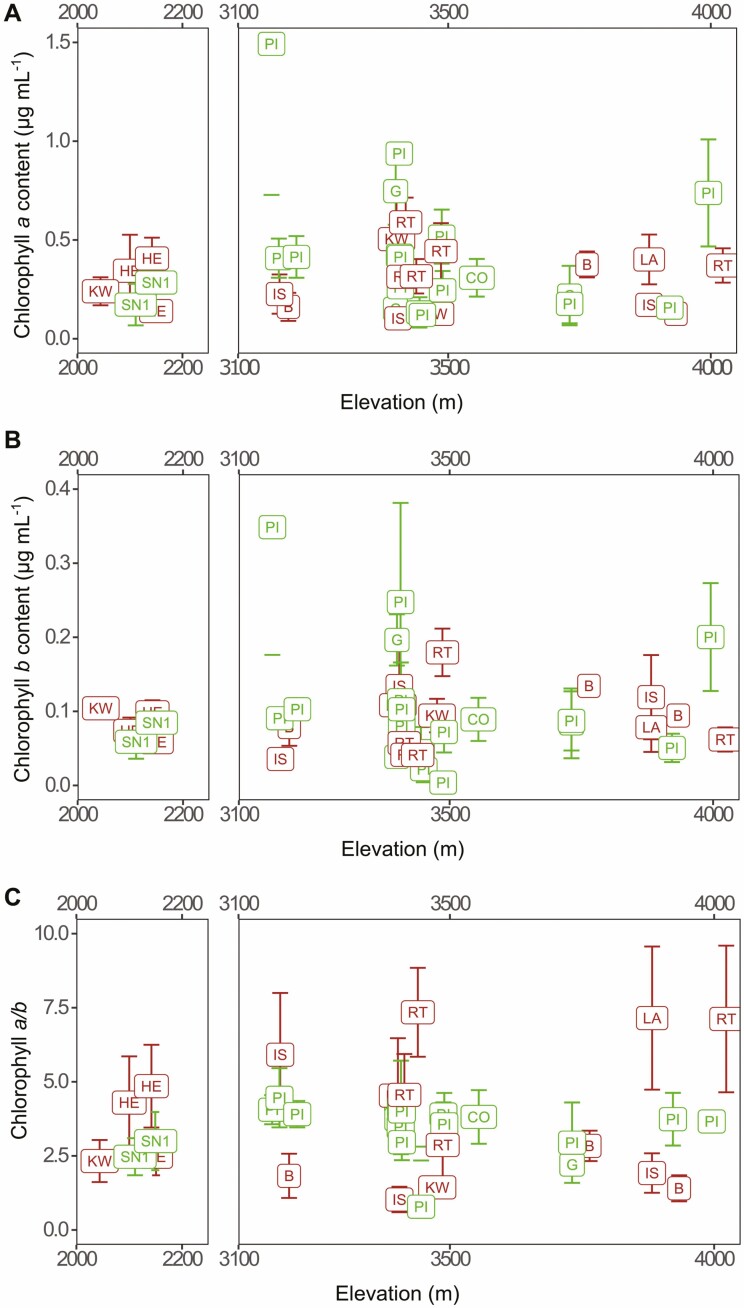
Chlorophyll content of *Lobaria* thalli collected down an elevational gradient. Letters within boxplots refer to the species of **Lobaria*.* The chloro-*Lobaria* were: *L. costata* (CO), *L. pindarensis* (PI), *Lobaria* sp. 1 (SN1) and other chloro-*Lobaria* (G). The chloro-cyano-*Lobaria* were: *L. hengduanensis* (HE), *L. isidiosa* (IS), *L. kurokawae* (KW), *L. latilobulata* (LA), *L. retigera* (RT), and other chloro-cyano-*Lobaria* (B).

All specimens tested contained both chlorophyll *a* and *b*, demonstrating the presence of green algae (Fig. 6A and B). The highest recorded values of chlorophyll *a* and *b* were for the most abundant chlorolichen, *L. pindarensis.* Surprisingly, chlorophyll *b* content was rarely lower in chloro-cyano-*Lobaria* than the lowest values recorded for chloro-*Lobaria* at the same elevation (Fig. 6A and B) indicating that these chloro-cyano-*Lobaria* had acquired a comparable number of green algal photobiont cells as their chloro-*Lobaria* neighbours.

There were significant interactions with elevation in the variance of chlorophyll content of chloro-*Lobaria* versus chloro-cyano-*Lobaria* (morphotype), as well with individual *Lobaria* spp., and green algae spp. (Supporting Information 2: Table S4). The lowest elevation sites never had high chlorophyll *a* or *b*. At all other elevations, there was higher variation in chlorophyll *a* and *b* for chloro-*Lobaria* but higher variation in chlorophyll *a/b* for chloro-cyano-*Lobaria* (Fig. 6A–C). For chloro-cyano-*Lobaria*, the highest chlorophyll contents were at ≈ 3500 m, but variance in chlorophyll *a/b* increased markedly with elevation. In contrast, for chloro-*Lobaria*, chlorophyll *a/b* was comparatively constant both between species and with elevation.

### Microscopy

Thallus cross-sections viewed under the microscope confirmed the presence of green algae in all the new *Lobaria* specimens included in this study. Neither cephalodia nor individual *Nostoc* cells were observed in any chloro-*Lobaria* thalli. Relative abundance of *Nostoc* and chlorobionts varied across each chloro-cyano*-Lobaria* thallus. In Fig. 1 we present photographs from different *Lobaria*—photobiont species associations, contrasting green algal photobiont layers in chloro-*Lobaria* thalli (Fig. 1B and C) to *Nostoc* cells (Fig. 1D) and sections of chloro-cyano-*Lobaria* in which *Nostoc* and green algae are present in a common layer (Fig. 1F, G, H, J, K, L, O, P, Q, S, U).

### Photosynthesis after addition of liquid water

Freshly collected *Lobaria* specimens immediately began dark respiration after addition of liquid water. There was a longer delay for chloro-cyano-*Lobaria* than chloro-*Lobaria* between light exposure and attaining net photosynthesis. Dark acclimation of moist thalli before placement in the main lichen chambers reduced this time-lag and avoided re-saturation respiration causing unrealistically high CO_2_ concentrations within the chambers. One hour of dark acclimation was sufficient for chloro-*Lobaria,* but two hours dark acclimation was required for chloro-cyano-*Lobaria.* Specimens showed no light inhibition at any light intensity tested. Net photosynthetic rates (NP) were always greater at CO_2_ concentrations of 500 µmol mol^−1^, than at 400 µmol mol^−1^ or 320 µmol mol^−1^.

### Re-activation of photosynthesis by desiccated lichen

**Figure 7.**
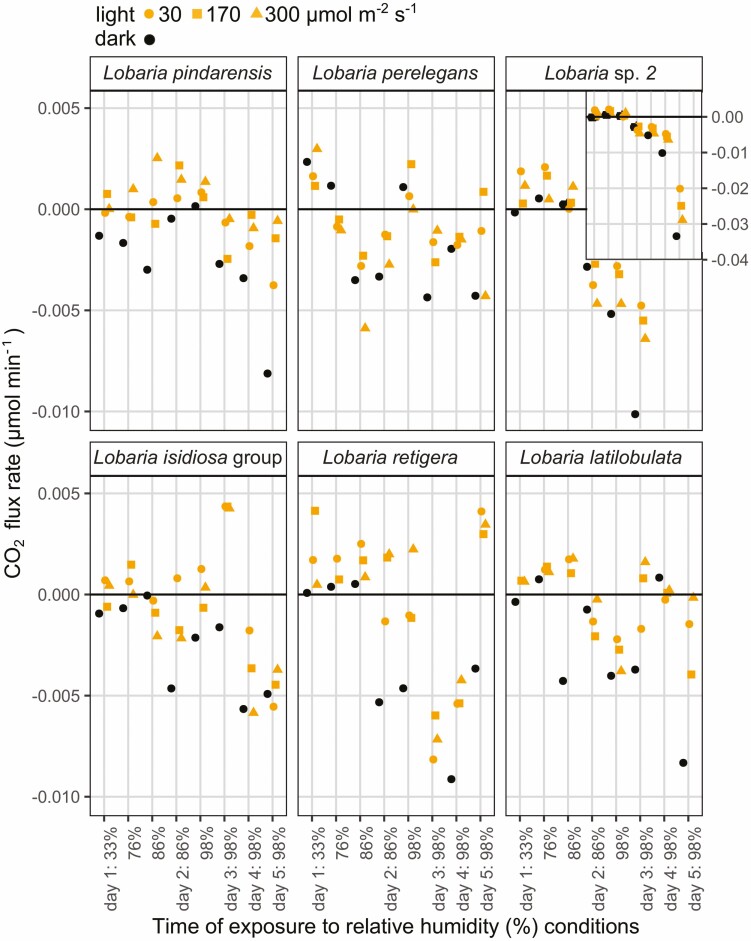
CO_2_ flux rates (µmol min^-1^) through the total system circuit, for a representative set of six individual specimens. The top row are chlorolichens and the bottom row are chloro-cyanolichens. The system circuit during these records included the LICOR, tubing and lichen chamber with a lichen specimen inside. Specimens were exposed to progressively increasing relative humidity (%) conditions. Change in CO_2_ was measured first in the dark, then with light exposure of 30, 170 and 300 µmol m^−2^ s^−1^. Mycobiont species is noted at the top of each panel.

**Figure 8.**
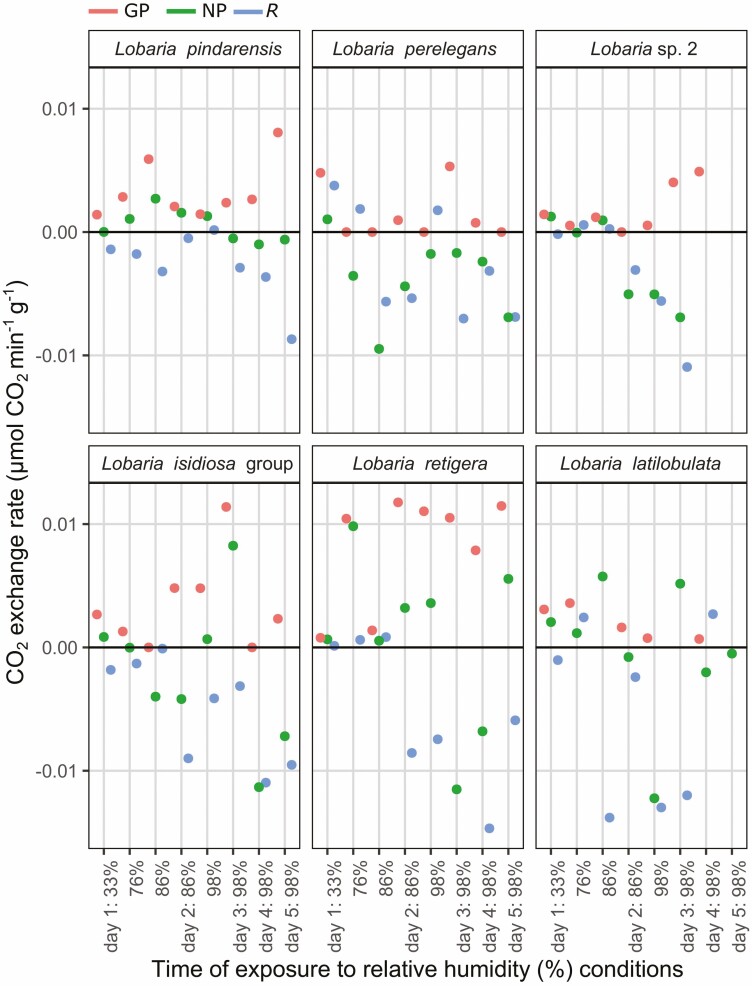
Photosynthetic response of six individual desiccated *Lobaria* specimens after exposure to 300 µmol m^−2^ s^−1^ light and progressively increased relative humidity (%), shown according to mycobiont species. The top row are chlorolichens and the bottom row are chloro-cyanolichens. Data are displayed for gross photosynthetic rate (GP), net photosynthetic rate (NP) and dark respiration (*R*). N.B. y-axes are curtailed. See Supporting Information 1: Fig. S11 for maximum values of GP and minimum values of *R.*

**Figure 9.**
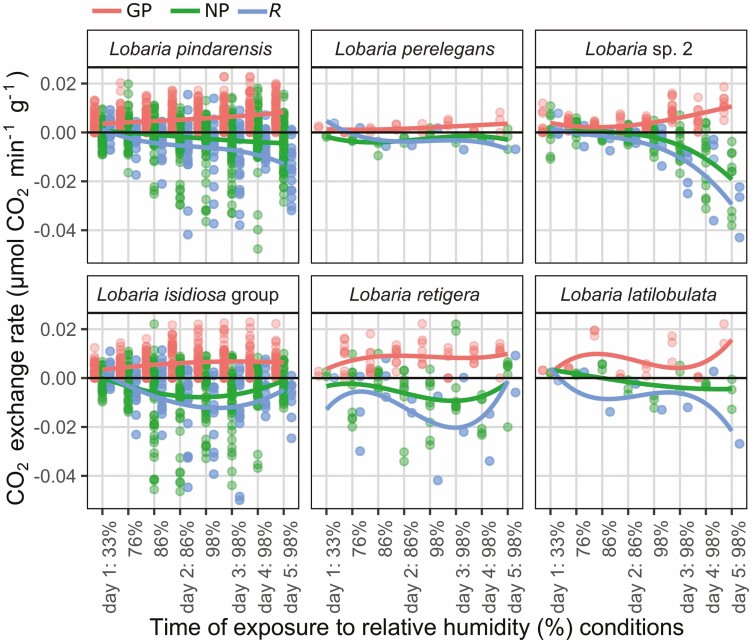
Photosynthetic response of desiccated *Lobaria* specimens after exposure to light and progressively increased relative humidity (%), shown according to mycobiont species. The top row are chlorolichens and the bottom row are chloro-cyanolichens. Data are displayed for gross photosynthetic rate (GP), net photosynthetic rate (NP) and dark respiration (*R*).

**Figure 10.**
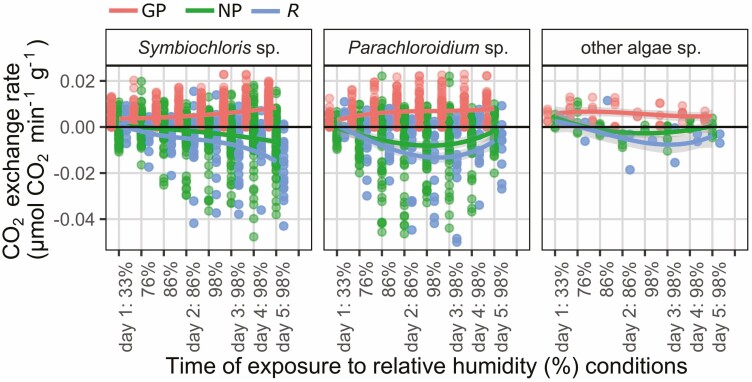
Photosynthetic response of desiccated *Lobaria* specimens after exposure to light and progressively increased relative humidity (%), shown according to green algal photobiont clades. Data are displayed for gross photosynthetic rate (GP), net photosynthetic rate (NP) and dark respiration (*R*).

**Figure 11.**
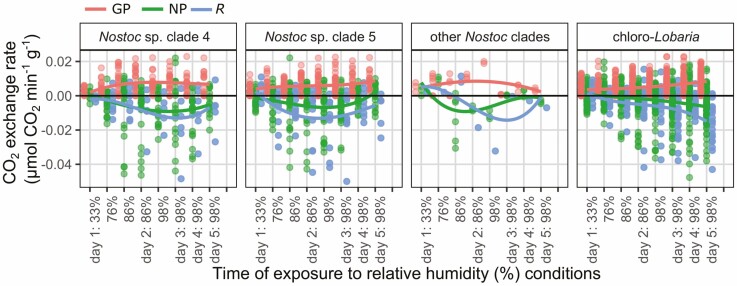
Photosynthetic response of desiccated *Lobaria* specimens after exposure to light and progressively increased relative humidity (%), shown according to *Nostoc* photobiont strains. Data are displayed for gross photosynthetic rate (GP), net photosynthetic rate (NP) and dark respiration (*R*).

The same *Lobaria* specimens for which photosynthesis had been tested after addition of liquid water, were stored dry for over four months, after which they were able to re-activate photosynthesis at 33% relative humidity (RH): conditions usually considered as desiccating (Figs. 7–11). Most specimens showed immediate negative CO_2_ flux rates (Fig. 7) attributed to dark respiration (*R*), upon placement in the lichen chamber. Conversely, some specimens showed positive CO_2_ fluxes (Fig. 7) and exchange rates (Figs. 8–11, Supporting Information 1: Fig. S11) in the dark, presumably indicating carbon fixation through nitrogenase activity of the mycobiont (Rai *et al.* 1981). The weakest response was by *L. perelegans* (Fig. 9), but there was no statistically significant difference in *R* between *Lobaria* spp. or chlorobiont genera. Unexpectedly, there was a significant effect of *Nostoc* strain on *R* (Supporting Information 2: Table S5) which also had a marginally significant effect on NP.

Once these specimens were exposed to light, CO_2_ flux rates became less negative or more positive; indicating an increase in CO_2_ uptake which we hereafter attribute to photosynthesis. In the examples of six individual specimens shown in Fig. 7, for five species all three light conditions (30, 170 or 300 µmol m^−2^ s^−1^) were sufficient to cause increased CO_2_ uptake. For *L. perelegans,* CO_2_ uptake only increased with exposure to 300 µmol m^−2^ s^−1^ (Fig. 7).

The results indicate that many specimens immediately began photosynthesis at 33% RH (Figs. 8–11). All chlorobiont clades were able to re-activate photosynthesis. The highest gross photosynthetic rate (GP) (0.0132 µmol CO_2_ min^−1^) was recorded for *L. pindarensis* with *S. reticulata*. However, overall GP was significantly higher for *Parachloroidium* and *L. retigera* and lower for *L. perelegans* and *Lobaria* sp. 2 (Supporting Information 2: Table S5). For some specimens of all mycobionts GP was sufficiently higher than *R* that they were able to achieve positive NP (Fig. 9). There was high individual variation between specimens; for some the magnitude of *R* was equal to or greater than GP (e.g. *L. pindarensis* at 98% RH in Fig. 8). There was no significant difference in NP between mycobionts, but those with *Parachloroidium* as chlorobiont had marginally significantly lower NP (Supporting Information 2: Table S5).

### Photosynthesis after exposure to humid air

The majority of specimens became more active as they were exposed to air of 76% RH. Both *R* and GP increased, but some specimens of both chlorobionts and all mycobionts (except *L. perelegans*) were able to maintain positive NP (Figs. 9 and 10). When specimens were given humid air of 86% then 98% RH, their responses began to markedly diverge (Figs. 7–11, Supporting Information 1: Fig. S11, Supporting Information 2: Table S6). In most cases, during the dark CO_2_ fluxes became progressively more negative, while during the light CO_2_ flux rates continued to become less negative or more positive (Fig. 7). This indicates that both *R* and GP increased with longer periods of time at higher RH (Figs. 8–11). Increasing *R* appeared to be the main determinant of whether positive NP could be maintained. Overall, NP was significantly lower when the chlorobionts were *Parachloroidium.* For NP, GP and *R* there were significant interactions between humidity exposure and mycobiont identity (Supporting Information 2: Table S6).

For specimens with *S. reticulata* as chlorobiont, there was significantly higher GP for those with *L. pindarensis* as the mycobiont. For specimens with *Parachloroidium* as chlorobiont, there was overall significantly higher GP for those with *L. retigera* as the mycobiont (Supporting Information 2: Table S6).

CO_2_ exchange rates differed between *Nostoc* strains (Fig. 11). When *L. isidiosa* had *Parachloroidium* as chlorobiont, having *Nostoc* 5 was associated with higher NP and *R* (Fig. 11, Supporting Information 2: Table S6). Differences in rates of *Nostoc* dark respiration or rates of dark CO_2_ fixation via nitrogenase activity might account for these apparent differences in NP.

CO_2_ exchange rates of these specimens were also calculated as a percentage of their own respiration, gross and net photosynthetic rates when they had been initially hydrated with liquid water (*R*%, GP% and NP%). After 24 h extended exposure to 86% or 98% RH, *L. isidiosa* and *L. retigera* were able to attain maxima of 43.1 NP% and 33.6 NP%, respectively. Surprisingly, these values were higher than for chloro-*Lobaria* (maximum 13.7 NP% for *L. pindarensis*). This was probably due to their chlorobiont, as across all experimental conditions *Parachloroidium* reached higher maximum and mean NP% and GP% than did *S. reticulata* (Supporting Information Table S7).

Progressively increasing humidity conditions caused greater proportional increases in *R*% (maximum = 72.3%) than for GP% or NP%, obscuring sequential changes in GP% and NP% (Supporting Information 1: Figs. S12, S13, S14, Supporting Information 2: Tables S7, S8). However, time series analysis showed significant differences in NP%, GP% and *R*% between both mycobionts and chlorobionts, with significant interactions with humidity conditions (Supporting Information 2: Table S8). When the chlorobiont was *S. reticulata,* GP% was significantly higher for *L. pindarensis.* When the chlorobiont was *Parachloroidium*, NP% was significantly higher for *L. retigera*. There was an additional effect of *Nostoc* identity on *R*% (Supporting Information 2: Table S8).

## Discussion

### All species could photosynthesise without liquid water

All lichen species in this study were recorded as attaining positive net photosynthetic rates without requiring liquid water. Given their very low carbon exchange rates, it could be questioned whether these results truly represent photosynthesis rather than leaks from the experimental circuit. It is possible that despite the steps taken to avoid this problem, in some cases leaks might have arisen during the course of an experiment, which would have reduced the accuracy of our measurements. However, such problems could not account for the light responses which were consistently observed during the course of these experiments, as no leak could repeatedly cause carbon exchange rates to change after light exposure.

An alternative explanation might be that the observed reduction in CO_2_ release in response to light results from the suppression of dark respiration by light. For plants, daytime respiratory metabolism involves changes in major pathways, which reduces CO_2_ efflux relative to that in darkness (Tcherkez *et al.* 2017). However, this effect would not account for positive net CO_2_ uptake. Despite the potential imprecision of our data, mentioned above, it does strongly suggest positive net CO_2_ uptake with light exposure. Moreover, the chamber CO_2_ concentration was typically below that in the laboratory, such that any leaks would have tended to cause overestimation of apparent respiration rate and hence underestimation of apparent net photosynthesis.

We consider that our experiment clearly showed that chloro-cyano-*Lobaria* had the ability to photosynthesise relying on air humidity alone. This is likely to be an advantageous trait. After 24 hours at 86% or 98% RH, *L. isidiosa* and *L. retigera* attained maxima of 43.1% and 33.6% respectively of their NP when hydrated with liquid water (NP%). Surprisingly, this photosynthetic re-activation exceeded that of chloro-*Lobaria* (maximum 13.7 NP% for *L. pindarensis*). For all lichen associations, mean NP% was lower than *R. capatita’*s recorded rates of 25 NP%, 60 NP% and 90 NP%, after 24 hours at RH of 85%, 90% and 95%, respectively (Pintado and Sancho 2002). Mean NP% recovery in our study was also lower than Phinney *et al.* (2019) records of ≈ 30 NP% (at 86% RH), and ≈ 50 NP% to 80 NP% (at 95% RH), chlorophyll fluorescence recovery for *L. pulmonaria* and *Lobaria virens* (With.) J.R. Laundon with photobiont *S. reticulata.* This is potentially because our specimens had been desiccated for a longer time period prior to our experiment.

Not only were lichens in our study capable of photosynthesis after tissue hydration by extended exposure to high humidity, but they were also capable of photosynthesis at unprecedentedly low RH. Attaining positive NP at 33% to 76% RH would extend their photosynthetically active period throughout daylight hours. We propose that this is likely to be especially advantageous during the dry season.

Relative humidity of 75% and below causes thallus dehydration, not hydration (Carniel *et al.* 2021), resulting in functionally dry thalli. However, photosynthesis should theoretically still be possible. Desiccation of *Flavoparmelia caperata* (L.) Hale thalli at 75% RH leaves molecules in a ‘rubbery state’, at which enzymatic activity, such as the xanthophyll cycle, persists. Xanthophyll conversion can still be detected at 55% RH (Carniel *et al.* 2021). *Peltigera polydactyla* (Neck.) Hoffm. and *Ramalina celastri* (Spreng.) A. Massal. can switch from limited triboxycarbolic acid cycle and transamination reactions to biochemical dormancy at 54%–40% RH and 45%–30% RH, respectively (Cowan *et al.* 1979). By 5% RH, cellular fluids have entered a ‘glassy state’ at which enzymatic activity ceases (Carniel *et al.* 2021).

### Microclimate records

During the dry season, the *Lobaria* spp. in our study would be exposed to extended periods with low relative humidity (RH). During daylight hours, RH would frequently remain between 25% and 50%, thus there could be considerable selective advantage to any lichens which could reach positive NP at such low RH%. Photosynthesis would become attainable throughout the day, rather than being limited to occasional, short periods of dew at dawn.

### Chloro-cyano-*Lobaria* were distributed along the elevational gradient, with chlorophyll *b* content comparable to chloro-*Lobaria*

All putative cyano-*Lobaria* specimens collected in this study had acquired chlorobionts, regardless of their elevation. Thus, these specimens should be considered chloro-cyano-*Lobaria.* We cannot draw any conclusions about whether the same species would be chloro-cyanolichens or cyanolichens elsewhere. Previous records of *Lobaria* specimens with the morphology of cyanolichens, should provisionally still be treated as cyano-*Lobaria*, and have been referred to as such in the phylogenetic trees.

The presence of chlorobionts was confirmed via: cross-sections of thalli, chlorophyll *b* content, and gene sequencing. For all specimens, cross-sections showed a photobiont layer incorporating green algae. In chloro-cyano-*Lobaria,* chlorobionts and cyanobionts were present in a common layer (resembling that found by Henskens *et al.* 2012).

All samples of chloro-cyano-*Lobaria* spp. had chlorophyll *b* content that fell within the range of values for chloro-*Lobaria*. This strongly indicates that their chlorobionts and cyanobionts are co-primary photobionts. It seems unlikely that a substantive portion of chlorophyll *b* could have derived from green algal epibionts, as these were not observed and the triple pre-wash with CaCO_3_ saturated acetone (Barnes *et al.* 1992) would be likely to remove any epibionts that were present.

Chlorophyll *b* content did not support the hypothesis that the proportion of green algal cells would be greater for chloro-cyano-*Lobaria* specimens collected from higher elevations. However, the lowest elevational limit of *Lobaria* was ≈ 2000 m, thus any especial selective pressures associated with high elevation may have been present at all study sites. Tree canopy closure differed between collection sites, so some variation in chlorophyll content could have arisen from differences in light exposure (Gauslaa *et al*. 2006). We did not record canopy cover nor sunlight exposure at each specimen’s microsite; so, we cannot determine whether differences in these factors could have masked the impact of differences in elevation.

The multiple records of chloro-cyano*-Lobaria* in this study raises the question as to why such observations have never previously been reported from the genus *Lobaria*. Possibly, researchers have simply missed this occurrence, due to not taking cross-sections, not checking chlorophyll *b* content and not using primers for photobionts expected to be absent. However, given the extensive research conducted on *Lobaria*, it seems unlikely that this is the sole reason.

We suggest two explanations. Firstly, that comparison to far lower sites would reveal that factors linked to elevation are indeed the driver for green algae acquisition by chloro-cyanolichens (Devkota *et al.* 2019). Secondly, that this is a unique region for species diversification. In Yunnan, a complex geological past has formed geographically isolated plant populations, which have undergone divergent evolution (Zhu and Tan 2022). Of China’s endemic plant species, at least 483 are in Diqing and 549 in Lijiang (Qian *et al.* 2020). Recent studies suggest that our study region has been a diversification hotspot for *Lobaria* (Miao *et al.* 2018; Yang *et al.* 2022). Phylogeny of species pairs (*L. kurokawae* versus *L. retigera* and *L. pulmonaria* versus *L. isidiosa*) also indicates that transition from asexual to sexual morphs occurred on at least one occasion within Yunnan (Cornejo *et al.* 2009).

### Chloro-cyano-*Lobaria* rarely shared green algae with chloro-*Lobaria*

There was some support for hypothesis 3: in five cases chloro-cyano-*Lobaria* potentially acquired photobionts from chloro-*Lobaria* in our study region. All chloro-*Lobaria* possessed *S. reticulata*. This accords with previous studies in the Himalayas (Devkota *et al.* 2019). Elsewhere, some chloro-*Lobaria* are associated with *Symbiochloris tschermakiae* and *S. reticulata* (Škaloud *et al.* 2016). For our chloro-cyano-*Lobaria,* there were only four specimens (of *L. isidiosa, L. kurokawae* and *L. retigera*) with *S. reticulata* as photobiont.

Our *Lobaria* sp. 1 was the only case of chloro-*Lobaria* with *Trebouxia* clade 1 and *S. reticulata* as joint photobionts. Although photobiont switches can drive speciation (Steinová *et al.* 2022), this appears unlikely as another *Lobaria* sp. 1 specimen lacked *Trebouxia*. One *L. kurokawae* specimen had *Trebouxia* clade 1 and *S. reticulata* as joint photobionts. With only one case of each association, it cannot be discerned if one species acquired *Trebouxia* from the other. The closest published *Trebouxia* sequences were free-living on soil, rock or snow, suggesting that our *Lobaria* spp. might have independently acquired *Trebouxia* from nearby substrates. Alternatively, *Trebouxia* could have been acquired from other neighbouring lichen associations, as *Trebouxia* are common lichen photobionts (Muggia *et al.* 2020; Sanders and Masumoto 2021).

In the majority of cases, chloro-cyano-*Lobaria* had acquired *Parachloroidium*. Our *Parachloroidium* clade has no closely related sequences in GenBank. It could be speculated that a single initial green algal acquisition event had been followed by diversification of *Parachloroidium*. But this appears unlikely, as *Parachloroidium* occurred in multiple chloro-cyano-*Lobaria* spp. Furthermore, there were certainly at least three other independent instances of green algal acquisition: of *Chloroidium, Apatococcus* and *Pseudochlorella.* All these genera can occur as free-living algae (Neustupa *et al.* 2013; Darienko *et al.* 2016, 2018; Gustavs *et al.* 2016).

Muggia *et al.* (2013) demonstrated that from unwashed thalli, sequences were recovered of four genera of epibiontic green algae, that were absent from samples that had been rigorously cleaned (brushed and repeatedly washed with sterile water and Tween80). This has raised the concern that supposed photobiont sequences obtained from lichen samples might actually be from epibionts (Sanders and Masumoto 2021). However, it should be noted that in Muggia *et al.* (2013), the epibiontic green algae were clearly visible and their method (single-strand conformation polymorphism) would have been deliberately chosen to enable simultaneous detection of multiple genomic variants.

We cannot completely exclude the possibility that some sequences were derived from scarce epibiontic green algae which were not observed, rather than a true photobiont. However, it would be improbable for this to have occurred on multiple occasions, as Sanger sequencing is only expected to identify the most abundant photobiont (Paul *et al.* 2018). Rather, photobiont diversity within thalli might exceed that demonstrated in this study, with non-primary chlorobionts also present. Techniques such as high-throughput sequencing would be needed to reveal green algal diversity (Paul *et al.* 2018). For these reasons we hold to the position that green algae sequenced from thalli in this study represent abundant internal chlorobionts.

### Acquisition of green algae has been a local process: green algal photobiont species differ between regions

The highest chlorobiont diversity was found in Diqing, with sequences belonging to eight clades of green algae obtained from these specimens. Geographical isolation of Honghe cloud forests also corresponded to differing mycobiont-photobiont associations. Two *Lobaria* spp. and three green algal clades were only found in Honghe. Such differences in photobionts between regions indicates that acquisition of green algae could have been a local process. However, sampling across more geographically separated regions would be needed for more rigorous testing of this hypothesis.

Cyanobiont associations also showed some regional differences. Those in Honghe were *Nostoc* 1 or *Nostoc* 6, rather than the *Nostoc* 4 and 5 strains which were most commonly found elsewhere. Most *Nostoc* strains shown in our phylogeny have previously been recorded in other lichen genera. Elsewhere, *Nostoc* strains vary between being present in many differing mycobiont species, to having high reciprocal selectivity towards specialist mycobionts (Magain *et al.* 2017; Kaasalainen *et al.* 2021). Our strains *Nostoc* 3, 4, 6 and 7 have no closely matching published sequences. We hypothesise that they might have genetically diverged locally, and be limited to *Lobaria* in the study region. Sequencing of *Nostoc* from other cyanolichen genera in our region would be required to test this conjecture.

### Mycobiont and photobiont identity both impacted photosynthetic rates

Photosynthetic rates differed between both mycobionts and photobionts, according to humidity conditions and with time period of exposure to each humidity condition. Overall, the chloro-cyanolichens *L. isidiosa* and *L. retigera* (containing *Parachloroidium*) were able to attain NP and GP comparable to, or in some instances even higher, than that of *L. pindarensis* with *S. reticulata*.

In the specific cases of *S. reticulata* and *Parachloroidium*, the responses of mycobionts differed even when they had the same photobiont. For *Parachloroidium*; GP and *R* were higher in *L. retigera* than other *Lobaria* spp. For *S. reticulata*; GP was highest for *L. pindarensis*. This demonstrated that photobiont identity is probably not the sole determinant of NP and GP, despite the photobiont conducting all photosynthesis.

Mycobionts can modulate photosynthetic rates during water stress by influencing water status; via impacting thallus turgor and osmotic potential. They also contribute to the degree of dehydration-induced decrease in photosystem functionality (*F*_v_/*F*_m_) (Petruzzellis *et al.* 2018) and mechanisms quenching PSII fluorescence during desiccation (Kosugi *et al.* 2009). Additional mycobiont impacts on photosynthesis include: generating CO_2_ which is transferred to the photobiont (Ten Veldhuis *et al.* 2020) and enhanced photoprotection via melanisation (Kosugi *et al.* 2009; Beckett *et al.* 2019).

In our analysis, we identified distinct monophyletic sister clades within *Nostoc* based on the constructed phylogenetic tree, which were indicative of strain-level differentiation. This division was not predicated on geographical separation but was instead based on observable phylogenetic distinctions that correspond to variations in functional traits, specifically dark respiration rates among the strains. While we acknowledge the limitations inherent in using 16S rRNA sequences for precise strain delimitation, the identified monophyletic sister clades suggest the presence of functional diversity within *Nostoc*. These findings highlight the ecological significance of recognising strain-level diversity, even in closely related bacterial groups. Given the complexities of bacterial strain differentiation, future research employing more comprehensive genetic analyses could provide further clarity on the relationships and functional roles of *Nostoc* strains.

Intriguingly, *Nostoc* 5 appeared to contribute to recovery of photosynthesis. This directly contradicts all previous studies showing that cyanobacteria require liquid water (Lange *et al.* 1986, 1993; Budel and Lange 1991; [Bibr CIT0018][Bibr CIT0019]). Possibly this was an artefact of differing respiration rates, or of a comparatively low sample size after removing variance due to mycobiont and chlorobiont. Alternatively, *Nostoc* reduced chamber CO_2_ concentration via nitrogenase activity (Rai *et al.* 1981). This deserves further investigation. Scanning electron-microscopy studies such as that of Budel and Lange (1991) could be repeated to explore whether *Nostoc* strains might differ in the degree of cell compression after desiccation or in attaining turgidity after rehydration with water vapour.

### Conclusions

We present the first cases in which *Lobaria* spp. previously described as cyanolichens had acquired co-primary chlorobionts. The switch to a tripartite association with both cyanobacteria and green algae acquisition had occurred for seven clades of chloro-cyano-*Lobaria,* across three regions. Chloro-*Lobaria* primarily associated with *S. reticulata,* whereas chloro-cyano-*Lobaria* associated with *Parachloroidium, Symbiochloris, Trebouxia, Apatococcus, Pseudochlorella* and *Chloroidium.* Rarely, two green algal genera had been acquired by a single thallus. We propose that at least one advantage of acquiring chlorobionts is the ability to photosynthesise without liquid water. Notably, most chloro- and chloro-cyano-*Lobaria* spp. were able to recover net photosynthesis at unprecedentedly low relative humidity. This would greatly increase the time spent in a state of net carbon gain.

## Supporting Information

The following additional information is available in the online version of this article –

Supporting Information for this study, comprising Methods, Figures and Tables are available in three separate files in the online version of this article. These files consist of the following:

Supporting Information 1: Figures. Containing: Fig. S1: Study site map. Fig. S8: Average daily humidity and temperature. Fig. S9: Average half-hourly humidity and temperature. Fig. S10: Nostoc *16S* phylogenetic tree. Fig. S11: Photosynthetic response of individual specimens. Fig. S12: Photosynthetic response (%) according to mycobiont species. Fig. S13: Photosynthetic response (%) according to chlorobiont species. Fig. S14: Photosynthetic response (%) according to *Nostoc* species.

Supporting Information 2: Tables. Containing: Table S1: Gene markers and primer pairs used in this study. Table S2: Specimens with herbarium ID and GenBank accession numbers. Table S3: List of *Lobaria* mycobionts and their associated green algal and cyanobacterial photobionts. Table S4: Bartlett’s K^2^ test for difference in the variance of chlorophyll content. Table S5: Linear model for CO_2_ exchange rate at 33% humidity conditions. Table S6: Dynamic linear model for CO_2_ exchange rate at progressively increasing humidity conditions. Table S7: Summary statistics for CO_2_ exchange rate (reactivated as % fresh response). Table S8: Dynamic linear model for CO_2_ exchange rate (reactivated as % fresh response).

Supporting Information 3: Methods. Containing: Methods S1: Lichen dry mass. Methods S2: LICOR experiment—humidity control. Methods S3: LICOR experiment—Minimising leaks from the circuit. Methods S4: LICOR experiment—CO_2_ fluxes. This file also contains: Fig. S2: Thallus dry mass change. Fig. S3: Schematic diagram of LICOR photosynthesis experiment set-up. Fig. S4: Photographs of LICOR photosynthesis experiment set-up. Fig. S5: Construction of lichen chambers. Fig. S6: Checking circuits for leaks. Fig. S7: Examples of raw LICOR data.

plae025_suppl_Supplementary_Information_S1

plae025_suppl_Supplementary_Information_S2

plae025_suppl_Supplementary_Information_S3

## Data Availability

All gene sequences were deposited in GenBank. Lichen specimens were deposited in the KUN-L herbarium. The raw meteorological, chlorophyll and LICOR data that support the findings of this study, along with ‘R’ script for chlorophyll content calculations, are openly available in Mendeley data repository [DOI:10.17632/7c69d4ddr6.2].
