# Supplementary material for: Acquisition of green algal photobionts enables both chlorolichens and chloro-cyanolichens to activate photosynthesis at low humidity without liquid water
Source: AoB Plants. 2024 Apr 29;16(3):plae025. doi: 10.1093/aobpla/plae025 (PMC11102867; doi:10.1093/aobpla/plae025)
Supplement: plae025_suppl_Supplementary_Information_S1 [file plae025_suppl_supplementary_information_s1.pdf]

Acquisition of green algal photobionts enables both chlorolichens and chloro-cyanolichens to activate photosynthesis at low humidity without liquid water

Fiona Ruth Worthy, Douglas Allen Shaefer, Dhanushka Wanasinghe, Jian Chu Xu, Li Song Wang and Xin Yu Wang

Supporting Information 1: Figures

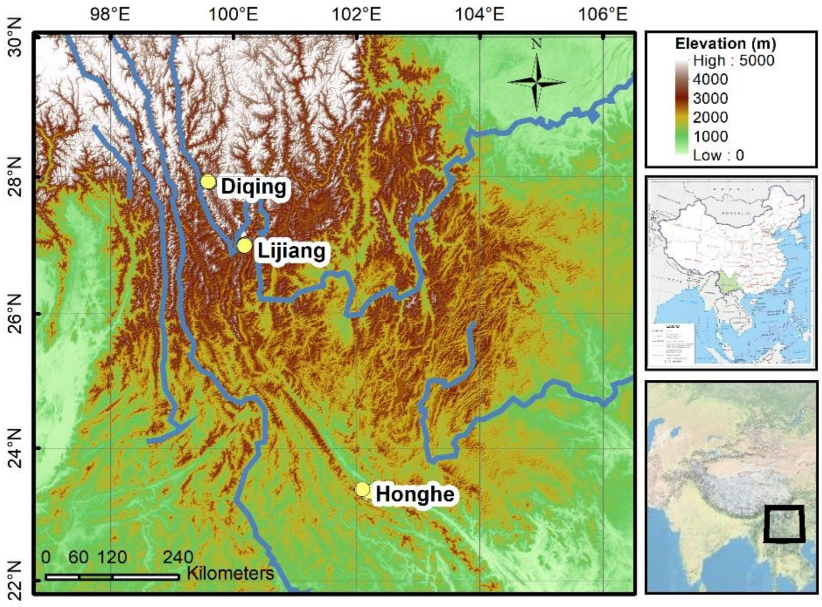

Figure S1. Study site location (left) within the trans-Himalayan region (bottom right). Yunnan Province (green shaded) within China (middle right).

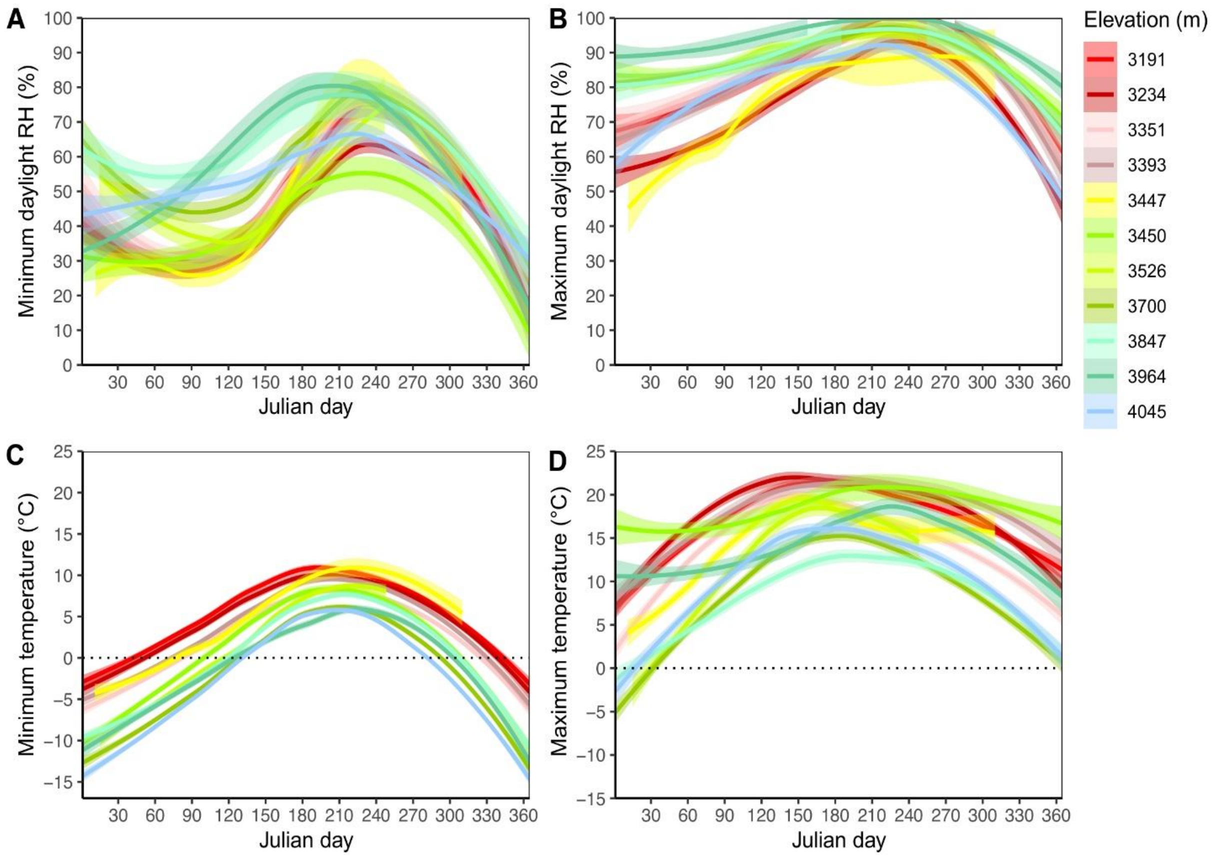

Figure S8. Average daily minimum and maximum relative humidity (RH %) and temperature (°C) along an elevational gradient.

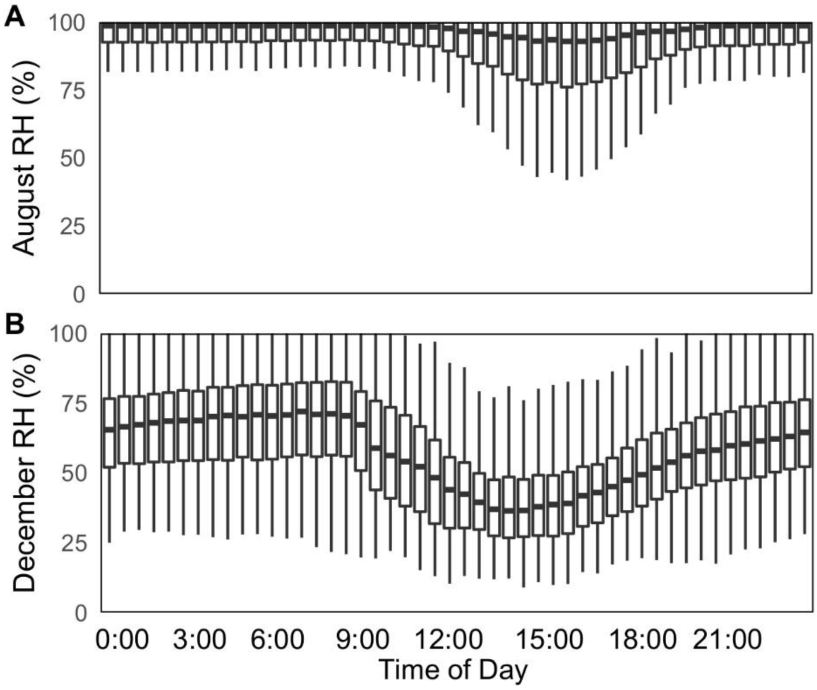

Figure S9. Average half-hourly relative humidity (RH %) for all study sites during (A) August and (B) December.

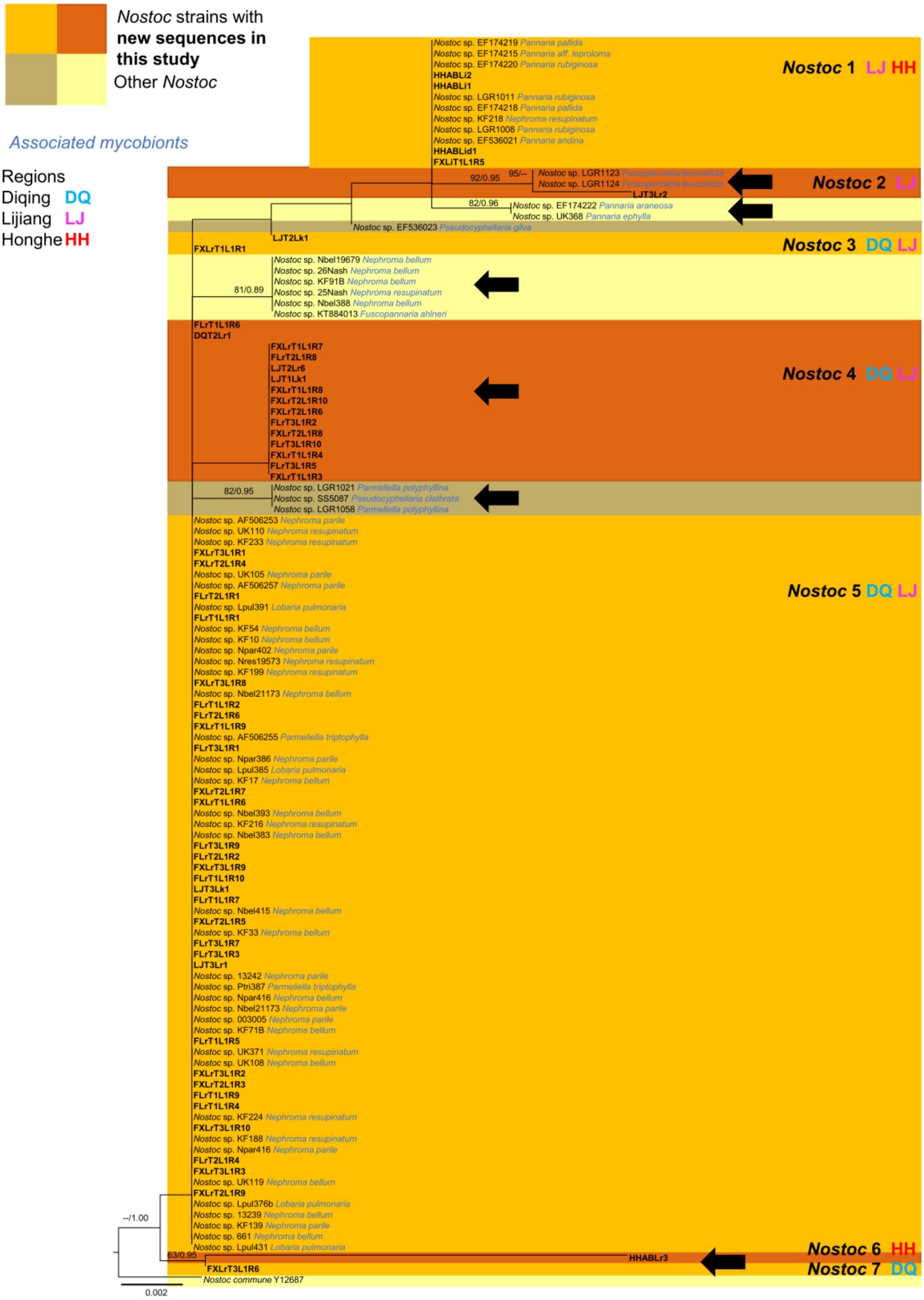

Figure S10. Maximum Likelihood (ML) phylogenetic tree for *Nostoc* based on *16S*. Nodes with ML bootstrap values ≥ 60% and posterior probabilities in BI (BYPP) ≥ 0.95 are indicated above each node. Their mycobiont is noted in blue italic font. Black arrows indicate monophyletic sister clades.

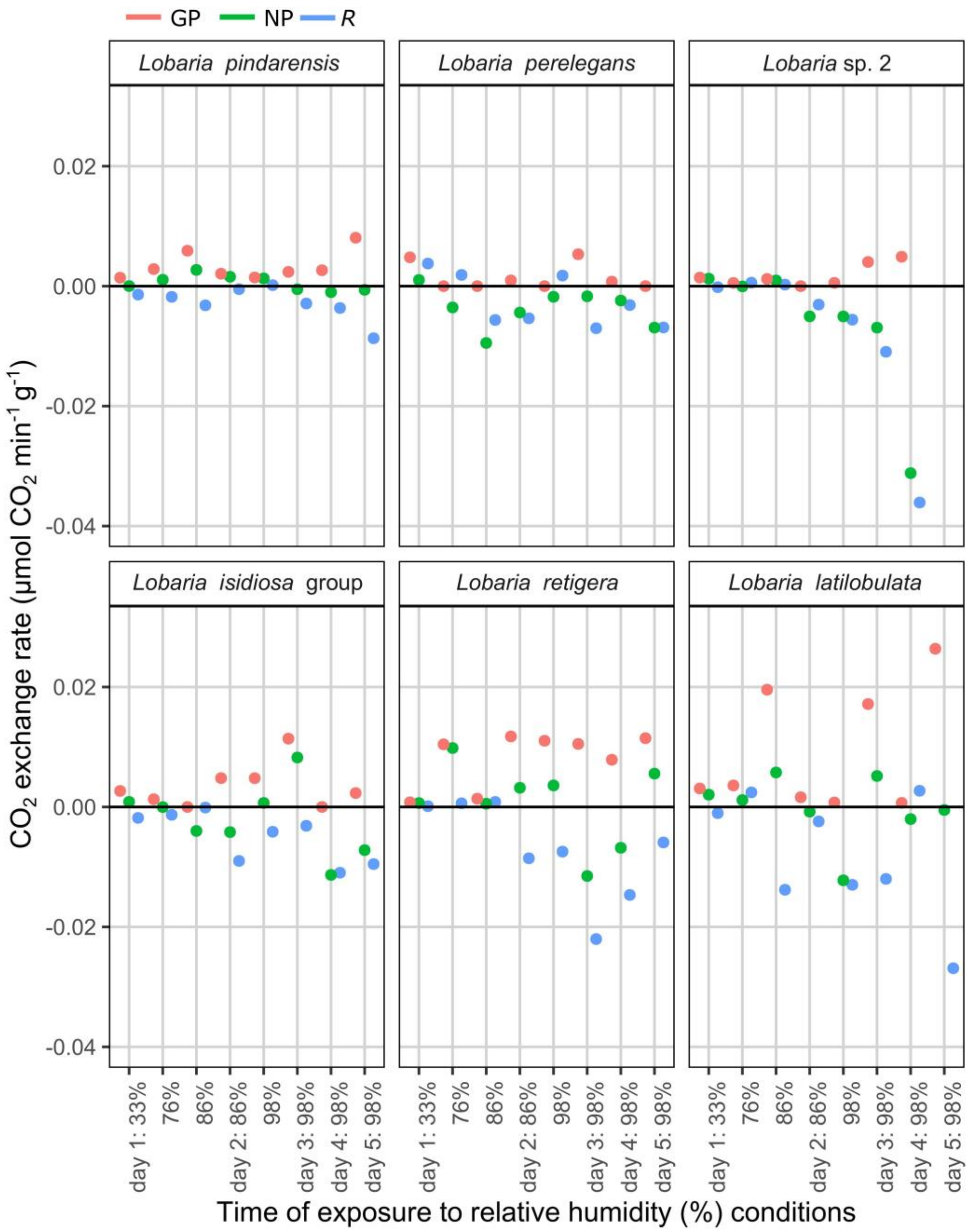

Figure S11. Photosynthetic response of six desiccated *Lobaria* specimens after exposure to 300 μmol m^{-2} s^{-1} light and progressively increased relative humidity (%), displayed as gross photosynthetic rate (GP), net photosynthetic rate (NP) and dark respiration (*R*). Mycobiont species is noted at the top of each panel. The top row are chlorolichens and the bottom row are chloro-cyanolichens.

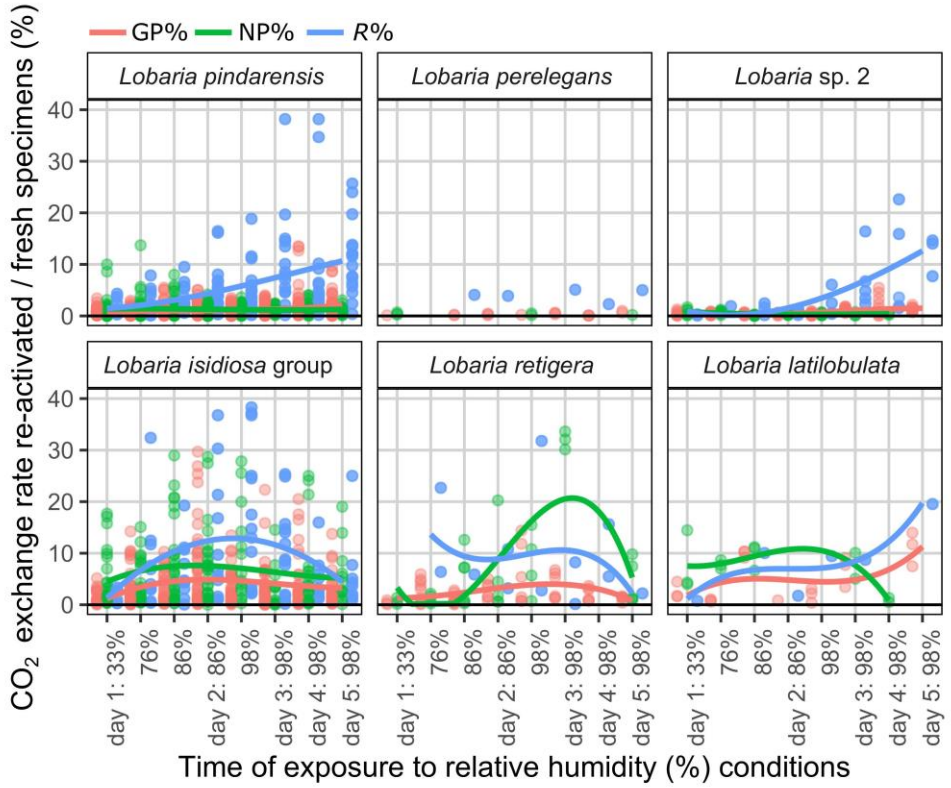

Figure S12. Photosynthetic response of *Lobaria* specimens shown according to mycobiont species. The top row are chlorolichens and the bottom row are chloro-cyanolichens. CO_2 exchange rates of desiccated *Lobaria* specimens after exposure to light and progressively increased relative humidity (re-activated) are shown as a percentage of the rates when the same specimen had been hydrated with liquid water at the time of its original collection. These are percentages of: gross photosynthetic rate (GP%), net photosynthetic rate (NP%) and dark respiration (*R*%). N.B. y-axis curtailed at 40%, see text for maximum values of *R*.

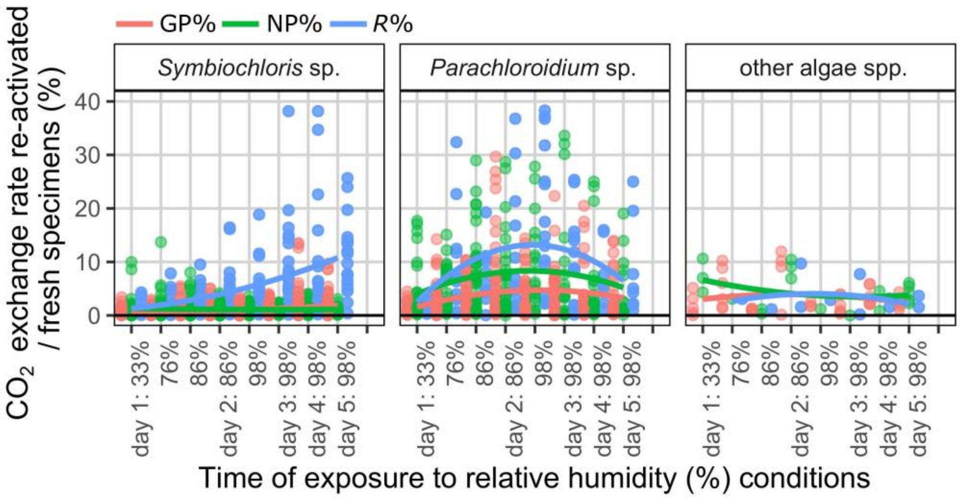

Figure S13. Photosynthetic response of *Lobaria* specimens shown according to green algal photobiont clades. CO_2 exchange rates of desiccated *Lobaria* specimens after exposure to light and progressively increased relative humidity (re-activated) are shown as a percentage of the rates when the same specimen had been hydrated with liquid water at the time of its original collection. These are percentages of: gross photosynthetic rate (GP%), net photosynthetic rate (NP%) and dark respiration (*R*%). N.B. y-axis curtailed at 40%, see text for maximum values of *R*.

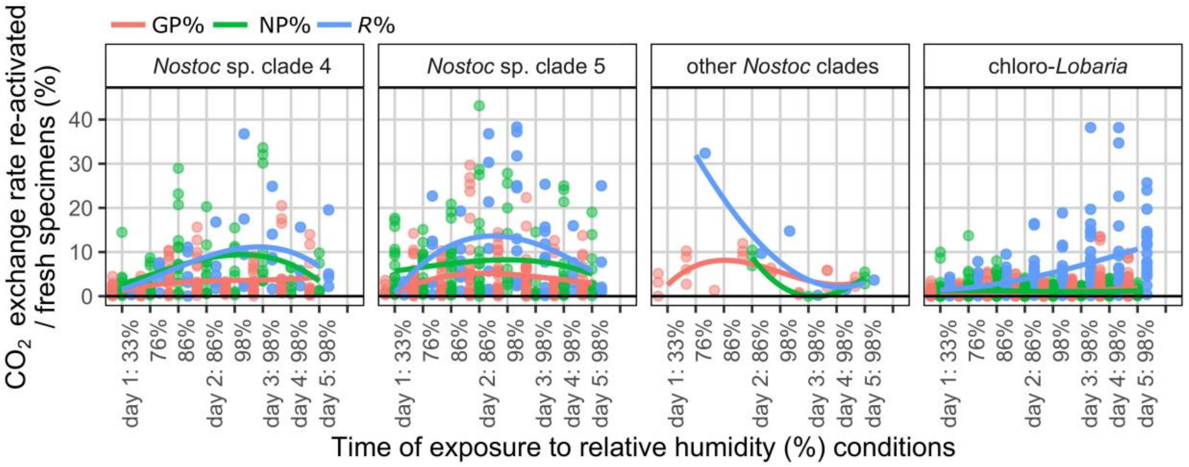

Figure S14. Photosynthetic response of *Lobaria* specimens shown according to *Nostoc* photobiont strains. CO_2 exchange rates of desiccated *Lobaria* specimens after exposure to light and progressively increased relative humidity (re-activated) are shown as a percentage of the rates when the same specimen had been hydrated with liquid water at the time of its original collection. These are percentages of: gross photosynthetic rate (GP%), net photosynthetic rate (NP%) and dark respiration (*R*%). N.B. y-axis curtailed at 50%, see text for maximum values of *R*.
