# Supplementary material for: Acquisition of green algal photobionts enables both chlorolichens and chloro-cyanolichens to activate photosynthesis at low humidity without liquid water
Source: AoB Plants. 2024 Apr 29;16(3):plae025. doi: 10.1093/aobpla/plae025 (PMC11102867; doi:10.1093/aobpla/plae025)
Supplement: plae025_suppl_Supplementary_Information_S2 [file plae025_suppl_supplementary_information_s2.pdf]

Acquisition of green algal photobionts enables both chlorolichens and chloro-cyanolichens to activate photosynthesis at low humidity without liquid water

Fiona Ruth Worthy, Douglas Allen Shaefer, Dhanushka Wanasinghe, Jian Chu Xu, Li Song Wang and Xin Yu Wang

Supporting Information 2: Tables

Table S1. Gene markers and primer pairs used in this study.

Gene markers	Primers	Primer sequences	References
ITS	ITS1F	CTTGGTCATTTAGAGGAAGTAA	(White *et al.* 1990)
	ITS4	TCCTCCGCTTATTGATATGC	(Gardes and Bruns 1993)
EF-1α	Lp-EF-1α-F	RGACAAGRCTCACATCAACGTGGT	(Johannesson *et al.* 2000)
	Lp-EF-1α -R	CCAGTGATCATGTTCTTGATGAAGT	(Johannesson *et al.* 2000)
RPB2	Lp-RPB-F	CAAACCGCGTCAACTGCATAAT	(Cornejo and Scheidegger 2010)
	Lp-RPB-R	CCCATGGCTTGCTTACCCAT	(Cornejo and Scheidegger 2010)
18S	a(treb)-nu-SSU-0078-5′-mpn	CATGTCTAAGTATAAACTGCT	(Dal Grande *et al.* 2014)
	a(treb)-nu-SSU-0803-3′-mpn	TAGGCCAGAGTCCTATCGTGTTAT	(Dal Grande *et al.* 2014)
RBC-L	rbcLAV	TGCAGCTGAATCGTCTACAG	(Dal Grande *et al.* 2014)
	rbcLIN	GTTGCGATCTTTTTCGATGT	(Dal Grande *et al.* 2014)
16S	fD1	AGA GTT TGA TCC TGG CTC AG	(Weisburg *et al.* 1991)
	rD1	AAG GAG GTG ATC CAG CC	(Weisburg *et al.* 1991)

Supplemental Literature cited in Table S1

Cornejo C, Scheidegger C. 2010. *Lobaria macaronesica* sp. nov., and the phylogeny of *Lobaria* sect. *Lobaria* (Lobariaceae) in Macaronesia. *The Bryologist* 113: 590-604. DOI:10.1639/0007-2745-113.3.590

Dal Grande F, Beck A, Cornejo C, Singh G, Cheenacharoen S, Nelsen MP, Scheidegger C. 2014. Molecular phylogeny and symbiotic selectivity of the green algal genus *Dictyochloropsis* s.l. (Trebouxiophyceae): a polyphyletic and widespread group forming photobiont-mediated guilds in the lichen family Lobariaceae. *New Phytologist* 202: 455-470. DOI:10.1111/nph.12678

Gardes M, Bruns TD. 1993. ITS primers with enhanced specificity for basidiomycetes - application to the identification of mycorrhizae and rusts. *Molecular Ecology* 2: 113-118. DOI:10.1111/j.1365-294x.1993.tb00005.x

Johannesson HS, Johannesson KHP, Stenlid J. 2000. Development of primer sets to amplify fragments of conserved genes for use in population studies of the fungus *Daldinia loculata*. *Molecular Ecology* 9: 375-377. DOI:10.1046/j.1365-294x.2000.00874-6.x

Weisburg WG, Barns SM, Pelletier DA, Lane DJ. 1991. 16S Ribosomal DNA amplification for phylogenetic study. *Journal of Bacteriology* 173: 697-703. DOI:10.1128%2Fjb.173.2.697-703.1991

White T, Bruns T, Lee S, Taylor J. 1990. Amplification and direct sequencing of fungal ribosomal DNA genes for phylogenetics. In: Innis MA, Gelfand DH, Sninsky JJ, White TJ, eds. *PCR protocols: a guide to methods and applications.* San Diego: Academic Press, 315–321. DOI:10.1016/B978-0-12-372180-8.50042-1

Table S2

List of specimens and GenBank accession numbers for sequences used in this study. New sequences in this study are in bold font. Additional information is provided regarding country, region, collector and mycobiont. The substrate is noted for free-living cyanobacteria. If more than one sample was sequenced from a single specimen, these are noted as samples a or b. (a) *Lobaria* sequences *ITS-EF-1α-RPB* (b) green algae sequences *18S* & *RBC-L* (c) *Nostoc* sequences *16S.*

(a)

**Taxon**	**Specimen ID**	**GenBank number** *ITS*	*EF-1α*	*RPB2*	**Country**	**Region**	**Herbarium**	**Collector**	**Type specimen**
Lobaria anomala	CY/08	KC522962	KC602633	KC602559	Canada	British Columbia		Scheidegger	
L. anomala	X107	KC522963	KC602634	KC602591	USA	Alaska		Dillman	
L. bhutanica	40476	MW343627	MW393949	MW393984	Bhutan	Parao District	Scheidegger	C. Scheidegger	
L. bhutanica	40494	MW343629	MW393951	MW393986	Bhutan	Parao District	Scheidegger	C. Scheidegger	
L. bhutanica	40506	MW343630	MW393952	MW393987	Bhutan	Parao District	Scheidegger	C. Scheidegger	Type
L. bhutanica	40544	MW343628	MW393954	MW393989	Bhutan	Parao District	Scheidegger	C. Scheidegger	
L. chinensis	CT3/02b	MF151238	MF151190	MF151717	China	Taiwan	KUN-L	J.-T. Yang, F. Dal Grande, C. Scheidegger	
L. chinensis	CT3/02e	MF151237	MF151191	MF151732	China	Taiwan	KUN-L	J.-T. Yang, F. Dal Grande, C. Scheidegger	
L. costata	40081	MW343642	MW393941	MW393976	China	Yunnan	Scheidegger	C. Scheidegger, L.S. Wang, M.X. Yang	Type
L. costata	40112	MW343643	MW393942	MW393977	China	Yunnan	Scheidegger	C. Scheidegger, L.S. Wang, M.X. Yang	
L. costata	16-52050	MW343645			China	Xizang	KUN-L	L.S. Wang, M.X. Yang *et al.*	
L. costata	16-53682	MW343644			China	Xizang	KUN-L	L.S. Wang, M.X. Yang *et al.*	
L. costata	**DQT1Li2**	**OR335101**	**OR362941**		China	Yunnan,	KUN-L	F.R. Worthy	

						Diqing			
L. devkotae	18-62315	MW343617	MW393966	MW394002	China	Xizang	KUN-L	L.S. Wang, M.X. Yang *et al.*	
L. devkotae	19-64564	MZ724567	MW393963	MW393999	China	Xizang	KUN-L	L.S. Wang, M.X. Yang *et al.*	Type
L. devkotae	XY19-1105	MZ724618	MW393969	MW394005	China	Xizang	KUN-L	L.S. Wang *et al.*	
L. granulosa	40629	MW343625			Bhutan	Parao District	Scheidegger	C. Scheidegger	
L. granulosa	40635	MW343626			Bhutan	Parao District	Scheidegger	C. Scheidegger	
L. granulosa	XY19-1128	MW343624	MW393972	MW394008	China	Xizang	KUN-L	L.S. Wang, M.X. Yang *et al.*	Type
L. gyrophorica	CT16/01f	MF151248	MF151178	MF151707	Taiwan		KUN-L	J.-T. Yang, F. Dal Grande, C. Scheidegger	
L. gyrophorica	TW2/01_8	MF151247	MF151180	MF151724	Taiwan		KUN-L	J.-T. Yang, F. Dal Grande, C. Scheidegger	
L. gyrophorica	TW2/26_2	MF151249	MF151181	MF151733	Taiwan		KUN-L	J.-T. Yang, F. Dal Grande, C. Scheidegger	
L. hengduanensis	19317	MW135388	MW654116	MW654145	China	Yunnan	KUN-L	Miao *et al.*	
L. hengduanensis	15-47615	MG653585			China	Yunnan	KUN-L	Miao *et al.*	
L. hengduanensis	15-48164	MG653584			China	Yunnan	KUN-L	Miao *et al.*	
L. hengduanensis	15-48562	MG653587			China	Yunnan	KUN-L	Miao *et al.*	
L. hengduanensis	**HHABLid1**	**OR335123**	**OR363023**		China	Yunnan, Honghe	KUN-L	F.R. Worthy	
L. hengduanensis	**HHABLr1**	**OR335124**	**OR363024**		China	Yunnan, Honghe	KUN-L	F.R. Worthy	
L. hengduanensis	**HHAMLr1**	**OR335126**	**OR363027**		China	Yunnan, Honghe	KUN-L	F.R. Worthy	
L. hengduanensis	**HHAMLr3**	**OR335128**	**OR363029**		China	Yunnan, Honghe	KUN-L	F.R. Worthy	
L. hengduanensis	KUN L51589	NR_171229			China	Yunnan	KUN-L	Miao *et al.*	Type
L. immixta	SCH-10462	GU072762	GU072876	GU072850	Spain	La Gomera	WSL	A. Werth	
L. immixta	SCH-10666	GU072767	GU072877	GU072853	Spain	Canary islands El Hierro	WSL	Werth	
L. immixta	SCH-10959	GU072763	GU072880	GU072845	Spain	Canary islands El Hierro	WSL	Werth	
L. immixta	SCH-3246	GU072764	GU072881	GU072839	Spain	La Palma	WSL	Windschuh	
L. irrugulosa	19015	MW135389	MW654110	MW654152	China	Yunnan	KUN-L	Miao *et al.*	

L. irrugulosa	19167	MW135390	MW654111	MW654151	China	Yunnan	KUN-L	Miao *et al.*
L. irrugulosa	KUN L55364	NR_171228			China	Yunnan	KUN-L	C.C. Miao, L.S. Wang
L. isidiophora	TW3/01_10	KC494095	KC602602	KC602542	China	Taiwan	KUN-L	J.-T. Yang, F. Dal Grande, C. Scheidegger
L. isidiophora	TW3/02_6	KC494091	KR476673	KR476701	China	Taiwan		Dal Grande & Scheidegger
L. isidiosa group	17-55509	MG653577			China	Yunnan	KUN-L	Miao *et al.*
L. isidiosa group	17-56325	MG653576			China	Yunnan	KUN-L	Miao *et al.*
L. isidiosa group	17-56337	MG653574			China	Yunnan	KUN-L	Miao *et al.*
L. isidiosa group	**DQT2Lr1**	**OR335103**	**OR362942**		China	Yunnan, Diqing	KUN-L	F.R. Worthy
L. isidiosa group	**FLrT1L1R1**	**OR335106**	**OR362945**	**OR363043**	China	Yunnan, Diqing	KUN-L	F.R. Worthy
L. isidiosa group	**FLrT1L1R10**	**OR335105**	**OR362946**	**OR363044**	China	Yunnan, Diqing	KUN-L	F.R. Worthy
L. isidiosa group	**FLrT1L1R2**	**OR335107**	**OR362947**	**OR363045**	China	Yunnan, Diqing	KUN-L	F.R. Worthy
L. isidiosa group	**FLrT1L1R4**	**OR335108**	**OR362948**	**OR363046**	China	Yunnan, Diqing	KUN-L	F.R. Worthy
L. isidiosa group	**FLrT1L1R5**	**OR335109**	**OR362949**		China	Yunnan, Diqing	KUN-L	F.R. Worthy
L. isidiosa group	**FLrT1L1R9**	**OR335112**	**OR362952**	**OR363048**	China	Yunnan, Diqing	KUN-L	F.R. Worthy
L. isidiosa group	**FLrT2L1R1**	**OR335113**	**OR362953**	**OR363049**	China	Yunnan, Diqing	KUN-L	F.R. Worthy
L. isidiosa group	**FLrT2L1R10**			**OR363050**	China	Yunnan, Diqing	KUN-L	F.R. Worthy
L. isidiosa group	**FLrT2L1R2**		**OR362954**	**OR363051**	China	Yunnan, Diqing	KUN-L	F.R. Worthy
L. isidiosa group	**FLrT2L1R4**	**OR335114**	**OR362955**	**OR363052**	China	Yunnan, Diqing	KUN-L	F.R. Worthy
L. isidiosa group	**FLrT3L1R10**	**OR335117**	**OR362959**	**OR363056**	China	Yunnan, Diqing	KUN-L	F.R. Worthy
L. isidiosa group	**FLrT3L1R2**	**OR335119**	**OR362960**	**OR363057**	China	Yunnan, Diqing	KUN-L	F.R. Worthy
L. isidiosa group	**FLrT3L1R3**	**OR335120**	**OR362961**	**OR363058**	China	Yunnan, Diqing	KUN-L	F.R. Worthy
L. isidiosa group	**FLrT3L1R7**		**OR362963**		China	Yunnan,	KUN-L	F.R. Worthy

						Diqing		
L. isidiosa group	**FLrT3L1R9**	**OR335122**	**OR362964**		China	Yunnan, Diqing	KUN-L	F.R. Worthy
L. isidiosa group	**FXLrT1L1R1**	**OR335178**	**OR362995**	**OR363085**	China	Yunnan, Diqing	KUN-L	F.R. Worthy
L. isidiosa group	**FXLrT1L1R10**		**OR362996**		China	Yunnan, Diqing	KUN-L	F.R. Worthy
L. isidiosa group	**FXLrT1L1R5**	**OR335181**	**OR362999**	**OR363087**	China	Yunnan, Diqing	KUN-L	F.R. Worthy
L. isidiosa group	**FXLrT1L1R6**	**OR335182**	**OR363000**	**OR363088**	China	Yunnan, Diqing	KUN-L	F.R. Worthy
L. isidiosa group	**FXLrT1L1R9**	**OR335185**	**OR363003**		China	Yunnan, Diqing	KUN-L	F.R. Worthy
L. isidiosa group	**FXLrT2L1R10**	**OR335186**	**OR363005**	**OR363090**	China	Yunnan, Diqing	KUN-L	F.R. Worthy
L. isidiosa group	**FXLrT2L1R2**	**OR335188**	**OR363006**		China	Yunnan, Diqing	KUN-L	F.R. Worthy
L. isidiosa group	**FXLrT2L1R3**	**OR335189**	**OR363007**		China	Yunnan, Diqing	KUN-L	F.R. Worthy
L. isidiosa group	**FXLrT2L1R4**	**OR335190**	**OR363008**		China	Yunnan, Diqing	KUN-L	F.R. Worthy
L. isidiosa group	**FXLrT2L1R5**	**OR335191**	**OR363009**		China	Yunnan, Diqing	KUN-L	F.R. Worthy
L. isidiosa group	**FXLrT2L1R6**	**OR335192**	**OR363010**		China	Yunnan, Diqing	KUN-L	F.R. Worthy
L. isidiosa group	**FXLrT2L1R7**	**OR335193**	**OR363011**	**OR363091**	China	Yunnan, Diqing	KUN-L	F.R. Worthy
L. isidiosa group	**FXLrT2L1R8**	**OR335194**	**OR363012**		China	Yunnan, Diqing	KUN-L	F.R. Worthy
L. isidiosa group	**FXLrT2L1R9**	**OR335195**	**OR363013**	**OR363092**	China	Yunnan, Diqing	KUN-L	F.R. Worthy
L. isidiosa group	**FXLrT3L1R1**	**OR335197**	**OR363014**	**OR363093**	China	Yunnan, Diqing	KUN-L	F.R. Worthy
L. isidiosa group	**FXLrT3L1R10**	**OR335196**	**OR363015**	**OR363094**	China	Yunnan, Diqing	KUN-L	F.R. Worthy
L. isidiosa group	**FXLrT3L1R2**	**OR335198**	**OR363016**	**OR363095**	China	Yunnan, Diqing	KUN-L	F.R. Worthy
L. isidiosa group	**FXLrT3L1R3**	**OR335199**	**OR363017**	**OR363096**	China	Yunnan,	KUN-L	F.R. Worthy

						Diqing			
L. isidiosa group	**FXLrT3L1R5**	**OR335200**	**OR363018**	**OR363097**	China	Yunnan, Diqing	KUN-L	F.R. Worthy	
L. isidiosa group	**FXLrT3L1R6**	**OR335201**	**OR363019**	**OR363098**	China	Yunnan, Diqing	KUN-L	F.R. Worthy	
L. isidiosa group	**FXLrT3L1R7**		**OR363020**	**OR363099**	China	Yunnan, Diqing	KUN-L	F.R. Worthy	
L. isidiosa group	**FXLrT3L1R8**	**OR335202**	**OR363021**	**OR363100**	China	Yunnan, Diqing	KUN-L	F.R. Worthy	
L. isidiosa group	**FXLrT3L1R9**	**OR335203**	**OR363022**	**OR363101**	China	Yunnan, Diqing	KUN-L	F.R. Worthy	
L. isidiosa group	**LJT3Lk1**	**OR335145**	**OR363039**		China	Yunnan, Lijiang	KUN-L	F.R. Worthy	
L. isidiosa group	**LJT3Lr1**	**OR335146**	**OR363040**		China	Yunnan, Lijiang	KUN-L	F.R. Worthy	
L. kazawaensis	RS/104	KC522950	KC602604	KC602545	Russia	Sakhalin Island	Scheidegger	C. Scheidegger, S. Chabanenko, Taran	
L. kurokawae 3	CY/15	DQ419937		KC602543	China	Yunnan	WSL	C. Scheidegger	
L. kurokawae	17-56156	MG653591			China	Yunnan	KUN-L	Miao *et al.*	
L. kurokawae	17-56158	MG653590			China	Yunnan	KUN-L	Miao *et al.*	
L. kurokawae	CY/18	DQ419938	KC602603	KC602544	Russia	Sakhalin Oblast		Chabanenko	
L. kurokawae	**HHAMLr2**	**OR335127**	**OR363028**		China	Yunnan, Honghe	KUN-L	F.R. Worthy	
L. kurokawae	**LJT2Li2**	**OR335136**			China	Yunnan, Lijiang	KUN-L	F.R. Worthy	
L. kurokawae	**LJT3Lr2**	**OR335147**	**OR363041**		China	Yunnan, Lijiang	KUN-L	F.R. Worthy	
L. latilobulata	17-55944	MG653578			China	Yunnan	KUN-L	Miao *et al.*	
L. latilobulata	**DQT2Lr2**		**OR362943**		China	Yunnan, Diqing	KUN-L	F.R. Worthy	
L. latilobulata	**FLrT3L1R5**	**OR335121**	**OR362962**		China	Yunnan, Diqing	KUN-L	F.R. Worthy	
L. latilobulata	KUN L59367	NR_171227			China	Yunnan	KUN-L	C.C. Miao, L.S. Wang	Type
L. ligulata	XY19-1114	MW343646	MW393970	MW394006	China	Xizang	KUN-L	L.S. Wang, M.X. Yang *et al.*	Type
L. ligulata	XY19-1119	MW343647	MW393971	MW394007	China	Xizang	KUN-L	L.S. Wang, M.X. Yang *et*	

								al.	
L. linita	CH	KC494125	KC602605	KC602546	Switzerland			Scheidegger	
L. linita	RS_63	KC494123	KC602606	KC602547	Russia	Sakhalin Oblast		Scheidegger, Chabanenko, Taran	
L. linita	X122	KC494124	KC602607	KC602548	Switzerland	Julier Pass		Werth, Cornejo	
L. macaronesica	SCH-12243	GU072741	GU072886	GU072844	Portugal	Sintra	WSL	Scheidegger	
L. macaronesica	SCH-12545	GU072746	GU072884	GU072863	Portugal	Madeira	WSL	Werth, Scheidegger	
L. macaronesica	SCH-13099	GU072747	GU072885	GU072838	Portugal	Madeira	WSL	Werth, Scheidegger	Type
L. macaronesica	SCH-2151	GU072740	GU072883	GU072864	Spain	La Gomera	WSL	Nittinger	
L. meridionalis	377/1	MF151257	MF151207	MF151734	Russia	Primorsky Krai	Scheidegger	S. Chabanenko	
L. meridionalis	377/2	KC522960	KC602631	KC602588	Russia	Primorsky Krai	Scheidegger	S. Chabanenko	
L. multipartita	40459	MW343638	MW393947	MW393982	Bhutan	Parao District	Scheidegger	C. Scheidegger	
L. multipartita	19-63350	MW343637	MW393956	MW393991	China	Xizang	KUN-L	L.S. Wang, M.X. Yang *et al.*	
L. multipartita	XY19-501	MW343639		MW394009	China	Xizang	KUN-L	L.S. Wang, M.X. Yang *et al.*	
L. multipartita	XY19-525	MW343636	MW393974	MW394011	China	Xizang	KUN-L	L.S. Wang, M.X. Yang *et al.*	Type
L. orientalis	40486	MW343635	MW393950	MW393985	Bhutan	Parao District	Scheidegger	C. Scheidegger	
L. orientalis	004/15	MF151223	MF151172	MF151695	Russia	Primorsky Krai	Scheidegger	S. Chabanenko	
L. orientalis	010/1	MF151230	MF151184	MF151698	Russia	Primorsky Krai	Scheidegger	S. Chabanenko	
L. perelegans	40623	MW343641			Bhutan	Parao District	Scheidegger	C. Scheidegger	
L. perelegans	19-65719	MW343640	MW393964	MW394000	China	Xizang	KUN-L	L.S. Wang, M.X. Yang *et al.*	Type
L. perelegans	**FXLiT1L1R4**	**OR335152**	**OR362969**		China	Yunnan, Lijiang	KUN-L	F.R. Worthy	
L. pindarensis	**DQT2Li2**	**OR335102**			China	Yunnan, Diqing	KUN-L	F.R. Worthy	
L. pindarensis	**LJT1Li1**	**OR335129**	**OR363030**		China	Yunnan, Lijiang	KUN-L	F.R. Worthy	
L. pindarensis	**LJT1Li2**	**OR335130**	**OR363031**		China	Yunnan, Lijiang	KUN-L	F.R. Worthy	

L. pindarensis	**LJT1Li7**		**OR363033**		China	Yunnan, Lijiang	KUN-L	F.R. Worthy
L. pindarensis	**LJT3Li5**	**OR335143**	**OR363037**		China	Yunnan, Lijiang	KUN-L	F.R. Worthy
L. pindarensis	**LJT3Li6**	**OR335144**	**OR363038**		China	Yunnan, Lijiang	KUN-L	F.R. Worthy
L. pindarensis	NE23/02a	KC494086	KC602010	KC602556	Nepal		Scheidegger	C. Scheidegger, S. Devkota
L. pindarensis	NE40/01b	MF151243	MF151196	MF151701	Nepal		Scheidegger	C. Scheidegger, S. Devkota
L. pindarensis	40552	MW343616	MW393955	MW393990	Bhutan	Paro District	Scheidegger	C. Scheidegger
L. pindarensis	19-64424	MW343613	MW393958	MW393994	China	Xizang	KUN-L	L.S. Wang, M.X. Yang *et al.*
L. pindarensis	19-64430	MW343615	MW393960	MW393996	China	Xizang	KUN-L	L.S. Wang, M.X. Yang *et al.*
L. pindarensis	19-64441	MW343614	MW393961	MW393997	China	Xizang	KUN-L	L.S. Wang, M.X. Yang *et al.*
L. pindarensis	**LJT1Li3**	**OR335131**	**OR363032**		China	Yunnan, Lijiang	KUN-L	F.R. Worthy
L. pindarensis	**LJT3Li2**	**OR335142**			China	Yunnan, Lijiang	KUN-L	F.R. Worthy
L. pindarensis	18-59984	MW269755	MW393957	MW393992	China	Xizang	KUN-L	L.S. Wang, M.X. Yang *et al.*
L. pindarensis	**DQT1Li1**	**OR335100**			China	Yunnan, Diqing	KUN-L	F.R. Worthy
L. pindarensis	**DQT3Li1**	**OR335104**			China	Yunnan, Diqing	KUN-L	F.R. Worthy
L. pindarensis	**FXLiT1L1R10**	**OR335148**	**OR362966**	**OR363062**	China	Yunnan, Lijiang	KUN-L	F.R. Worthy
L. pindarensis	**FXLiT1L1R3**	**OR335151**	**OR362968**		China	Yunnan, Lijiang	KUN-L	F.R. Worthy
L. pindarensis	**FXLiT1L1R5**	**OR335153**	**OR362970**		China	Yunnan, Lijiang	KUN-L	F.R. Worthy
L. pindarensis	**FXLiT1L1R9**	**OR335157**	**OR362974**	**OR363067**	China	Yunnan, Lijiang	KUN-L	F.R. Worthy
L. pindarensis	**FXLiT2L1R1**	**OR335159**	**OR362975**	**OR363068**	China	Yunnan, Lijiang	KUN-L	F.R. Worthy
L. pindarensis	**FXLiT2L1R10**	**OR335158**	**OR362976**		China	Yunnan, Lijiang	KUN-L	F.R. Worthy
L. pindarensis	**FXLiT2L1R2**	**OR335160**	**OR362977**	**OR363069**	China	Yunnan,	KUN-L	F.R. Worthy

						Lijiang		
L. pindarensis	**FXLiT2L1R3**	**OR335161**	**OR362978**	**OR363070**	China	Yunnan, Lijiang	KUN-L	F.R. Worthy
L. pindarensis	**FXLiT2L1R4**	**OR335162**	**OR362979**	**OR363071**	China	Yunnan, Lijiang	KUN-L	F.R. Worthy
L. pindarensis	**FXLiT2L1R4**	**OR335163**	**OR362980**	**OR363072**	China	Yunnan, Lijiang	KUN-L	F.R. Worthy
L. pindarensis	**FXLiT2L1R5**	**OR335164**	**OR362981**	**OR363073**	China	Yunnan, Lijiang	KUN-L	F.R. Worthy
L. pindarensis	**FXLiT2L1R6**	**OR335165**	**OR362982**	**OR363074**	China	Yunnan, Lijiang	KUN-L	F.R. Worthy
L. pindarensis	**FXLiT2L1R7**	**OR335166**	**OR362983**	**OR363075**	China	Yunnan, Lijiang	KUN-L	F.R. Worthy
L. pindarensis	**FXLiT2L1R8**	**OR335167**	**OR362984**	**OR363076**	China	Yunnan, Lijiang	KUN-L	F.R. Worthy
L. pindarensis	**FXLiT2L1R9**	**OR335168**	**OR362985**	**OR363077**	China	Yunnan, Lijiang	KUN-L	F.R. Worthy
L. pindarensis	**FXLiT3L1R1**	**OR335170**	**OR362986**	**OR363078**	China	Yunnan, Lijiang	KUN-L	F.R. Worthy
L. pindarensis	**FXLiT3L1R10**	**OR335169**	**OR362987**		China	Yunnan, Lijiang	KUN-L	F.R. Worthy
L. pindarensis	**FXLiT3L1R2**	**OR335171**	**OR362988**	**OR363079**	China	Yunnan, Lijiang	KUN-L	F.R. Worthy
L. pindarensis	**FXLiT3L1R3**	**OR335172**	**OR362989**	**OR363080**	China	Yunnan, Lijiang	KUN-L	F.R. Worthy
L. pindarensis	**FXLiT3L1R4**	**OR335173**	**OR362990**	**OR363081**	China	Yunnan, Lijiang	KUN-L	F.R. Worthy
L. pindarensis	**FXLiT3L1R6**	**OR335174**	**OR362991**	**OR363082**	China	Yunnan, Lijiang	KUN-L	F.R. Worthy
L. pindarensis	**FXLiT3L1R7**	**OR335175**	**OR362992**	**OR363083**	China	Yunnan, Lijiang	KUN-L	F.R. Worthy
L. pindarensis	**FXLiT3L1R8**	**OR335176**	**OR362993**	**OR363084**	China	Yunnan, Lijiang	KUN-L	F.R. Worthy
L. pindarensis	**FXLiT3L1R9**	**OR335177**	**OR362994**		China	Yunnan, Lijiang	KUN-L	F.R. Worthy
L. pindarensis	**LJT2Li1**	**OR335135**			China	Yunnan, Lijiang	KUN-L	F.R. Worthy
L. pindarensis	**LJT2Li3**	**OR335137**			China	Yunnan,	KUN-L	F.R. Worthy

						Lijiang		
L. pindarensis	**LJT2Li5**	**OR335138**			China	Yunnan, Lijiang	KUN-L	F.R. Worthy
L. pindarensis	**LJT3Li1**	**OR335141**	**OR363036**		China	Yunnan, Lijiang	KUN-L	F.R. Worthy
L. pindarensis	NE64/06a	MF151246	MF151198	MF151704	Nepal		Scheidegger	C. Scheidegger, S. Devkota
L. pseudopulmonaria	DQ419935	GU072900		Bhutan	Flor		Miehe	
L. pseudopulmonaria	SAKH 530	DQ419925	GU072901	GU072872	China	Yunnan		Chabanenko
L. pseudoretigera	LOB 07	KX679361			Malaysia	Sabah		N.H. Safie
L. pseudoretigera	LOB 08	KX679360			Malaysia	Sabah		N.H. Safie, C.S. Vairappan
L. pseudoretigera	LOB_04	KX679363			Malaysia	Sabah		N.H. Safie
L. pseudoretigera	LOB_05	KX679362			Malaysia	Sabah		N.H. Safie
L. pulmonaria	B13	MF151265	MF151214	MF151740	Switzerland			S. Scheidegger
L. pulmonaria	SCH-10660	GU072770	GU072896	GU072867	Spain	Canary Islands. El Hierro	WSL	Werth
L. pulmonaria	SCH-1073	GU072754	GU072894	GU072841	Spain	Canary Islands. El Hierro	WSL	Werth
L. retigera 1	CB/05	KC522952	KC602614	KC602562	Canada	British Columbia		Scheidegger
L. retigera 1	**FLrT1L1R6**	**OR335110**	**OR362950**		China	Yunnan, Diqing	KUN-L	F.R. Worthy
L. retigera 1	**FLrT1L1R7**	**OR335111**	**OR362951**	**OR363047**	China	Yunnan, Diqing	KUN-L	F.R. Worthy
L. retigera 1	**FLrT3L1R1**	**OR335118**	**OR362958**	**OR363055**	China	Yunnan, Diqing	KUN-L	F.R. Worthy
L. retigera 1	**FLrT3L1R4**			**OR363059**	China	Yunnan, Diqing	KUN-L	F.R. Worthy
L. retigera 1	**FLrT3L1R6**			**OR363060**	China	Yunnan, Diqing	KUN-L	F.R. Worthy
L. retigera 1	**FXLrT1L1R3**	**OR335179**	**OR362997**		China	Yunnan, Diqing	KUN-L	F.R. Worthy
L. retigera 1	**FXLrT1L1R4**	**OR335180**	**OR362998**	**OR363086**	China	Yunnan, Diqing	KUN-L	F.R. Worthy

L. retigera 1	**FXLrT1L1R8**	**OR335184**	**OR363002**		China	Yunnan, Diqing	KUN-L	F.R. Worthy	
L. retigera 1	**LJT1Lk2**	**OR335134**			China	Yunnan, Lijiang	KUN-L	F.R. Worthy	
L. retigera 1	**LJT2Lk1**	**OR335139**			China	Yunnan, Lijiang	KUN-L	F.R. Worthy	
L. retigera 2	**DQT3Lr1**		**OR362944**		China	Yunnan, Diqing	KUN-L	F.R. Worthy	
L. retigera 2	**FLrT2L1R6**	**OR335115**	**OR362956**	**OR363053**	China	Yunnan, Diqing	KUN-L	F.R. Worthy	
L. retigera 2	**FLrT2L1R8**	**OR335116**	**OR362957**	**OR363054**	China	Yunnan, Diqing	KUN-L	F.R. Worthy	
L. retigera 2	**FXLrT1L1R7**	**OR335183**	**OR363001**	**OR363089**	China	Yunnan, Diqing	KUN-L	F.R. Worthy	
L. retigera 2	**FXLrT2L1R1**	**OR335187**	**OR363004**		China	Yunnan, Diqing	KUN-L	F.R. Worthy	
L. retigera 2	**LJT1Lk1**	**OR335132**	**OR363034**		China	Yunnan, Lijiang	KUN-L	F.R. Worthy	
L. retigera 2	**LJT1Lr1**	**OR335133**	**OR363035**		China	Yunnan, Lijiang	KUN-L	F.R. Worthy	
L. rhizinata	40332	MW343620	MW393943	MW393978	China	Yunnan	Scheidegger	C. Scheidegger, L.S. Wang, M.X. Yang	
L. rhizinata	40411	MW343623	MW393946	MW393981	China	Yunnan	Scheidegger	C. Scheidegger, L.S. Wang, M.X. Yang	Type
L. sachalinensis	20-67877	MW534714			China	Qinghai	KUN-L	L.S. Wang *et al.*	
L. sachalinensis	RS/113a	KC522954	KC602616	KC602565	Russia	Sakhalin Oblast	Scheidegger	C. Scheidegger, S. Chabanenko, Taran	
L. sachalinensis	RS/121	KC522955	KC602617	KC602566	Russia	Sakhalin Oblast	Scheidegger	C. Scheidegger, S. Chabanenko, Taran	
Lobaria sp.	19176	MW135405	MW654112	MW654149	China		KUN-L	Miao *et al.*	
Lobaria sp.	19177	MW135406	MW654113	MW654148	China		KUN-L	Miao *et al.*	
Lobaria sp.	40362	MW343621	MW393944	MW393979	China	Yunnan	Scheidegger	C. Scheidegger, L.S. Wang, M.X. Yang	
Lobaria sp.	40396	MW343622	MW393945	MW393980	China	Yunnan	Scheidegger	C. Scheidegger, L.S. Wang, M.X. Yang	
Lobaria sp.	14-46398	MT110077			China		KUN-L	L.S. Wang, H. X. Shi	
Lobaria sp.	14-46417	MT110078			China		KUN-L	L.S. Wang, H. X. Shi	

Lobaria sp.	**LJT2Lr6**	**OR335140**			China	Yunnan, Lijiang	KUN-L	F.R. Worthy	
Lobaria sp.	XY19-1954	MW135415		MW654133	China		KUN-L	Miao *et al.*	
Lobaria sp. 1	**HHAMLi1**		**OR363025**		China	Yunnan, Honghe	KUN-L	F.R. Worthy	
Lobaria sp. 1	**HHAMLi2**	**OR335125**	**OR363026**		China	Yunnan, Honghe	KUN-L	F.R. Worthy	
Lobaria sp. 2	**FXLiT1L1R1**	**OR335149**	**OR362965**	**OR363061**	China	Yunnan, Lijiang	KUN-L	F.R. Worthy	
Lobaria sp. 2	**FXLiT1L1R2**	**OR335150**	**OR362967**	**OR363063**	China	Yunnan, Lijiang	KUN-L	F.R. Worthy	
Lobaria sp. 2	**FXLiT1L1R6**	**OR335154**	**OR362971**	**OR363064**	China	Yunnan, Lijiang	KUN-L	F.R. Worthy	
Lobaria sp. 2	**FXLiT1L1R7**	**OR335155**	**OR362972**	**OR363065**	China	Yunnan, Lijiang	KUN-L	F.R. Worthy	
Lobaria sp. 2	**FXLiT1L1R8**	**OR335156**	**OR362973**	**OR363066**	China	Yunnan, Lijiang	KUN-L	F.R. Worthy	
L. spathulata	RIT/04c	MF151252	MF151202	MF151723	Russia	Kurile Islands	Scheidegger	S. Chabanenko	
L. spathulata	RIT/04d	MF151253	MF151205	MF151719	Russia	Kurile Islands	Scheidegger	S. Chabanenko	
L. spathulata	RIT/08a	MF151254	MF151206	MF151709	Russia	Kurile Islands	Scheidegger	S. Chabanenko	
L. tibetana	40473	MW343633	MW393948	MW393983	Bhutan	Parao District	Scheidegger	C. Scheidegger	
L. tibetana	40538	MW343632	MW393953	MW393988	Bhutan	Parao District	Scheidegger	C. Scheidegger	
L. tibetana	18-62299	MW343631	MW393965	MW394001	China	Xizang	KUN-L	L.S. Wang, M.X. Yang *et al.*	
L. tibetana	XY19-1007	MW343634	MW393967	MW394003	China	Xizang	KUN-L	L.S. Wang, M.X. Yang *et al.*	Type
L. tuberculata	RS/95	KC522958	KC602628	KC602582	Russia	Sakhalin Oblast		C. Scheidegger, S. Chabanenko, Taran	
L. tuberculata	RS_62a	KC522956	KC602626	KC602580	Russia	Sakhalin Oblast		C. Scheidegger, S. Chabanenko, Taran	
L. tuberculata	RS_67	KC522957	KC602627	KC602581	Russia	Sakhalin Oblast		C. Scheidegger, S. Chabanenko, Taran	
L. tuberculata	X147	MF151262	MF151217	MF151727	Russia	Khabarovsk Krai		S. Chabanenko	
L. verruculosa	373/1	MF151255	MF151212	MF151737	Russia	Primorsky Krai	Scheidegger	S. Chabanenko	
L. verruculosa	374/1	MF151256	MF151209	MF151738	Russia	Primorsky	Scheidegger	S. Chabanenko	

						Krai			
L. verruculosa	XY19-505	MW343648	MW393973	MW394010	China	Xizang	KUN-L	L.S. Wang, M.X. Yang *et al.*	Type
L. wanglisongiana	16-53208	MW343649			China	Xizang	KUN-L	L.S. Wang, M.X. Yang *et al.*	
L. wanglisongiana	19-64427	MW343650	MW393959	MW393995	China	Xizang	KUN-L	L.S. Wang, M.X. Yang *et al.*	
L. wanglisongiana	19-64448	MW343651	MW393962	MW393998	China	Xizang	KUN-L	L.S. Wang, M.X. Yang *et al.*	
L. wanglisongiana	XY19-1049	MW343652	MW393968	MW394004	China	Xizang	KUN-L	L.S. Wang, M.X. Yang *et al.*	Type
L. yunnanensis	40008	MW343653	MW393940	MW393975	China	Yunnan	Scheidegger	C. Scheidegger, L.S. Wang, M.X. Yang	
L. yunnanensis	19-64420	MW343654		MW393993	China	Xizang	KUN-L	L.S. Wang, M.X. Yang *et al.*	
L. yunnanensis	CT10/02a	KC522959	KC602629	KC602586	China	Yunnan	Scheidegger	C. Scheidegger, L.S. Wang, M.X. Yang	
L. yunnanensis	XY19-526	MW343655			China	Xizang	KUN-L	L.S. Wang, M.X. Yang *et al.*	
**Outgroup**									
Lobarina scrobiculata	SCH-1443	GU072772	GU072903	GU072873	Russia	Sakhalin Oblast	WSL	Scheidegger, Chabanenko, Taran	
Lobarina scrobiculata	SCH-2928	GU072773	GU072902	GU072875	Canada	British Columbia	WSL	Scheidegger	

(b)

**Taxon**	**Specimen ID**	**GenBank number**		**Country**	**Region**	**Herbarium**	**Mycobiont**	**Substrate**
		18S	***RBC-L***					
Apatococcus fuscideae	AB 98_122B1	KY587795		Austria			*Fuscidea kochiana*	
Apatococcus lobatus	SAG 2359	JX169826		Germany			free-living	plastic waste bin
Apatococcus sp.	AEW1B_K142	KP081324					free-living	soil
Apatococcus sp.	BG-L-92374	KY587797		Norway				
Apatococcus sp.	**FXLrT1L1R7a**	**OR343858**		China	Lijiang, Yunnan	KUN-L	*Lobaria retigera*	
Chlorella sorokiniana	MIC-G5	JF834706		India				
Chlorella vulgaris	FACHB-1227		MK295221	China			free-living	
Chlorella vulgaris	SB1283-4	KX495036		China			free-living	fresh water
Chloroidium arboriculum	MG 3	MH551521	MH545954	Austria	Vienna		free-living	bark of *Fagus sylvatica*
Chloroidium augustoellipsoidium	BII/C-1	MH551507		Austria			free-living	bark
Chloroidium augustoellipsoidium	CCAP 211/33	MH551518		Italy				
Chloroidium augustoellipsoidium	SAG 2115	FM946019	MH545950	Germany	Gottingen		free-living	silicon insulation
Chloroidium ellipsoideum	CCAP 464/1	MH551516		Switzerland	Geneve			
Chloroidium ellipsoideum	SAG 2295	MH551517		USA	Hawaii			
Chloroidium saccharophilum	CCAP 211/58	MH551494		Antarctica	Mt Erebus			
Chloroidium sp.	**FXLrT1L1R3**	**OR343856**		China	Diqing, Yunnan	KUN-L	*Lobaria retigera*	
Dictyochloropsis asterochloroides	SAG 2073	KC333459	KC333605	Japan	Obayashi, Hiroshima		free-living	
Dictyochloropsis asterochloroides	SAG 2098	KC333460	KC333606	Japan	Yokogawa, Hiroshima		free-living	
Dictyochloropsis irregularis	NIES-378	GU017670						
Dictyochloropsis sp.	SA5513	KC333503		Columbia			*Lobariella pallidocrenulata*	
Dictyochloropsis splendida	CAUPH 8601	KC333457	KC333607	Czech Republic	Ceske		free-living	

					Stredohori Mts			
Dictyochloropsis splendida	FJ792796	FJ792796					*Lobaria pulmonaria*	
Dictyochloropsis splendida	JN573841		JN573841				*Lobaria pulmonaria*	
Dictyochloropsis splendida	SAG 2071	KC333456	KC333609	Japan	Nishi-kawaguchi-cho, Hiroshima		free-living	
Dictyochloropsis splendida	SAG 2097	KC333458	KC333608	Japan	Teramachi, Hiroshima		free-living	
Dictyochloropsis splendida	SAG 244.80	KC333474	KC333599	Austria	Graz		*Chaenotheca brunneola*	
Dictyochloropsis splendida	UTEX 2612	GU017660					*Phlyctis argena*	
Dictyochloropsis splendida	UTEX LB 2599		EF113435					
Myrmecia bisecta	SAG 2043		LC366921.1	Italy			free-living	soil
Myrmecia bisecta	Z47209	Z47209						
Myrmecia israeliensis	UTEX 1181		EF113453					
Myrmecia sp.	AEW2BK6	KP081351		Germany			free-living	soil
Myrmecia sp.	L3148		ON603533	Argentina			free-living	rock
Parachloroidium laureanum	CAUP H8501	HF586459	HF586462	Slovenia	Ankaran		free-living	bark sample
Parachloroidium lobatum	CAUP H8502	HF586460	HF586463	Slovenia	Pacug		free-living	bark sample of *Fraxinus ornus*
Parachloroidium lobatum	CAUP H8503	HF586461	HF586464	Italy	Policoro		free-living	corticolous biofilm on *Ficus carica* var. caprificus
Parachloroidium sp.	cort23		HG793079				free-living	corticolous biofilm
Parachloroidium sp.	cort25		HG793080				free-living	corticolous biofilm
Parachloroidium sp.	**DQT1Lr1**	**OR343819**		China	Diqing, Yunnan	KUN-L	*Lobaria* sp.	
Parachloroidium sp.	**FLrT1L1R1a**		**OR362864**	China	Diqing, Yunnan	KUN-L	*Lobaria isidiosa* group	
Parachloroidium sp.	**FLrT1L1R4**		**OR362866**	China	Diqing, Yunnan	KUN-L	*Lobaria isidiosa* group	
Parachloroidium sp.	**FLrT1L1R5**		**OR362867**	China	Diqing, Yunnan	KUN-L	*Lobaria isidiosa* group	

Parachloroidium sp.	**FLrT1L1R6**		**OR362868**	China	Diqing, Yunnan	KUN-L	*Lobaria retigera*
Parachloroidium sp.	**FLrT1L1R7**	**OR343824**	**OR362869**	China	Diqing, Yunnan	KUN-L	*Lobaria retigera*
Parachloroidium sp.	**FLrT2L1R1**	**OR343825**	**OR362871**	China	Diqing, Yunnan	KUN-L	*Lobaria isidiosa* group
Parachloroidium sp.	**FLrT2L1R4**	**OR343826**	**OR362873**	China	Diqing, Yunnan	KUN-L	*Lobaria isidiosa* group
Parachloroidium sp.	**FLrT2L1R6**		**OR362874**	China	Diqing, Yunnan	KUN-L	*Lobaria retigera*
Parachloroidium sp.	**FLrT2L1R8**		**OR362875**	China	Diqing, Yunnan	KUN-L	*Lobaria retigera*
Parachloroidium sp.	**FLrT3L1R1**		**OR362876**	China	Diqing, Yunnan	KUN-L	*Lobaria retigera*
Parachloroidium sp.	**FLrT3L1R10**		**OR362877**	China	Diqing, Yunnan	KUN-L	*Lobaria isidiosa* group
Parachloroidium sp.	**FLrT3L1R2**		**OR362878**	China	Diqing, Yunnan	KUN-L	*Lobaria isidiosa* group
Parachloroidium sp.	**FLrT3L1R3**		**OR362879**	China	Diqing, Yunnan	KUN-L	*Lobaria isidiosa* group
Parachloroidium sp.	**FLrT3L1R5**	**OR343827**	**OR362880**	China	Diqing, Yunnan	KUN-L	*Lobaria latilobulata*
Parachloroidium sp.	**FLrT3L1R7**		**OR362881**	China	Diqing, Yunnan	KUN-L	*Lobaria isidiosa* group
Parachloroidium sp.	**FLrT3L1R9**		**OR362882**	China	Diqing, Yunnan	KUN-L	*Lobaria isidiosa* group
Parachloroidium sp.	**FXLiT3L1R5**		**OR362909**	China	Lijiang, Yunnan	KUN-L	*Lobaria* sp.
Parachloroidium sp.	**FXLrT1L1R1**		**OR362914**	China	Diqing, Yunnan	KUN-L	*Lobaria isidiosa* group
Parachloroidium sp.	**FXLrT1L1R4**		**OR362915**	China	Diqing, Yunnan	KUN-L	*Lobaria retigera*
Parachloroidium sp.	**FXLrT1L1R5**	**OR343857**		China	Diqing, Yunnan	KUN-L	*Lobaria isidiosa* group
Parachloroidium sp.	**FXLrT1L1R6**		**OR362916**	China	Diqing, Yunnan	KUN-L	*Lobaria isidiosa* group
Parachloroidium sp.	**FXLrT1L1R7b**		**OR362917**	China	Diqing, Yunnan	KUN-L	*Lobaria retigera*

Parachloroidium sp.	**FXLrT1L1R8**		**OR362918**	China	Diqing, Yunnan	KUN-L	*Lobaria retigera*	
Parachloroidium sp.	**FXLrT2L1R10**		**OR362920**	China	Diqing, Yunnan	KUN-L	*Lobaria isidiosa* group	
Parachloroidium sp.	**FXLrT2L1R2**		**OR362921**	China	Diqing, Yunnan	KUN-L	*Lobaria isidiosa* group	
Parachloroidium sp.	**FXLrT2L1R3**		**OR362922**	China	Diqing, Yunnan	KUN-L	*Lobaria isidiosa* group	
Parachloroidium sp.	**FXLrT2L1R5**		**OR362923**	China	Diqing, Yunnan	KUN-L	*Lobaria isidiosa* group	
Parachloroidium sp.	**FXLrT2L1R6**		**OR362924**	China	Diqing, Yunnan	KUN-L	*Lobaria isidiosa* group	
Parachloroidium sp.	**FXLrT2L1R7**		**OR362925**	China	Diqing, Yunnan	KUN-L	*Lobaria isidiosa* group	
Parachloroidium sp.	**FXLrT2L1R8**	**OR343861**	**OR362926**	China	Diqing, Yunnan	KUN-L	*Lobaria isidiosa* group	
Parachloroidium sp.	**FXLrT2L1R9**		**OR362927**	China	Diqing, Yunnan	KUN-L	*Lobaria isidiosa* group	
Parachloroidium sp.	**FXLrT3L1R1**		**OR362928**	China	Diqing, Yunnan	KUN-L	*Lobaria isidiosa* group	
Parachloroidium sp.	**FXLrT3L1R10**		**OR362929**	China	Diqing, Yunnan	KUN-L	*Lobaria isidiosa* group	
Parachloroidium sp.	**FXLrT3L1R2**		**OR362930**	China	Diqing, Yunnan	KUN-L	*Lobaria isidiosa* group	
Parachloroidium sp.	**FXLrT3L1R3**		**OR362931**	China	Diqing, Yunnan	KUN-L	*Lobaria isidiosa* group	
Parachloroidium sp.	**FXLrT3L1R5**		**OR362932**	China	Diqing, Yunnan	KUN-L	*Lobaria isidiosa* group	
Parachloroidium sp.	**FXLrT3L1R8**		**OR362933**	China	Diqing, Yunnan	KUN-L	*Lobaria isidiosa* group	
Parachloroidium sp.	**FXLrT3L1R9**	**OR343862**	**OR362934**	China	Diqing, Yunnan	KUN-L	*Lobaria isidiosa* group	
Parachloroidium sp.	**LJT2Li2**	**OR343871**		China	Lijiang, Yunnan	KUN-L	*Lobaria kurokawae*	
Pseudochlorella pringsheimii	SAG 211-1a	KM020138	AM260446					
Pseudochlorella pyrenoidosa	SAG 18.95	LT560358		Italy	South Tyrol		free-living	soil
Pseudochlorella signiensis	SAG 2374	JX169837		Germany	Gottingen		free-living	on green plastic biowaste bin

Pseudochlorella sp.	**FLrT1L1R1b**	**OR343823**		China	Diqing, Yunnan	KUN-L	*Lobaria isidiosa* group	
Pseudochlorella sp.	**FXLrT2L1R1**	**OR343860**		China	Diqing, Yunnan	KUN-L	*Lobaria retigera*	
Pseudochlorella sp.	**LJT1Lr1**	**OR343870**		China	Lijiang, Yunnan	KUN-L	*Lobaria retigera*	
Symbiochloris irregularis	KR154345		KR154345	Austria			free-living	bark
Symbiochloris irregularis	SAG 2036	KC333475.1	JF502553	Austria				
Symbiochloris pauciautosporica	MPN167	KC333486	KC333596	Brazil			*Megalospora sulphurata*	
Symbiochloris pauciautosporica	UTEX 2599	GU017665					*Phlyctis argena*	
Symbiochloris reticulata	**DQT1Li1**	**OR343817**		China	Diqing, Yunnan	KUN-L	*Lobaria pindarensis*	
S. reticulata	**DQT1Li2**	**OR343818**		China	Diqing, Yunnan	KUN-L	*Lobaria costata*	
S. reticulata	**DQT2Li1a**	**OR343820**	**OR362862**	China	Diqing, Yunnan	KUN-L	*Lobaria* sp.	
S. reticulata	**DQT2Li1b**	**OR343821**	**OR362863**	China	Diqing, Yunnan	KUN-L	*Lobaria* sp.	
S. reticulata	**DQT3Li1**	**OR343822**		China	Diqing, Yunnan	KUN-L	*Lobaria pindarensis*	
S. reticulata	**FXLiT1L1R1**	**OR343828**	**OR362883**	China	Lijiang, Yunnan	KUN-L	*Lobaria* sp. 2	
S. reticulata	**FXLiT1L1R10**	**OR343829**	**OR362884**	China	Lijiang, Yunnan	KUN-L	*Lobaria pindarensis*	
S. reticulata	**FXLiT1L1R2**	**OR343830**	**OR362885**	China	Lijiang, Yunnan	KUN-L	*Lobaria* sp. 2	
S. reticulata	**FXLiT1L1R3**	**OR343831**	**OR362886**	China	Lijiang, Yunnan	KUN-L	*Lobaria pindarensis*	
S. reticulata	**FXLiT1L1R4**	**OR343832**	**OR362887**	China	Lijiang, Yunnan	KUN-L	*Lobaria perelegans*	
S. reticulata	**FXLiT1L1R5**	**OR343833**	**OR362888**	China	Lijiang, Yunnan	KUN-L	*Lobaria pindarensis*	
S. reticulata	**FXLiT1L1R6**	**OR343834**	**OR362889**	China	Lijiang, Yunnan	KUN-L	*Lobaria* sp. 2	
S. reticulata	**FXLiT1L1R7**	**OR343835**	**OR362890**	China	Lijiang, Yunnan	KUN-L	*Lobaria* sp. 2	
S. reticulata	**FXLiT1L1R8**	**OR343836**	**OR362891**	China	Lijiang,	KUN-L	*Lobaria* sp. 2	

					Yunnan		
S. reticulata	**FXLiT1L1R9**	**OR343837**	**OR362892**	China	Lijiang, Yunnan	KUN-L	*Lobaria pindarensis*
S. reticulata	**FXLiT2L1R1**	**OR343838**	**OR362893**	China	Lijiang, Yunnan	KUN-L	*Lobaria pindarensis*
S. reticulata	**FXLiT2L1R10**	**OR343839**	**OR362894**	China	Lijiang, Yunnan	KUN-L	*Lobaria pindarensis*
S. reticulata	**FXLiT2L1R2**	**OR343840**	**OR362895**	China	Lijiang, Yunnan	KUN-L	*Lobaria pindarensis*
S. reticulata	**FXLiT2L1R3**	**OR343841**	**OR362896**	China	Lijiang, Yunnan	KUN-L	*Lobaria pindarensis*
S. reticulata	**FXLiT2L1R4a**	**OR343842**	**OR362897**	China	Lijiang, Yunnan	KUN-L	*Lobaria pindarensis*
S. reticulata	**FXLiT2L1R4b**	**OR343843**	**OR362898**	China	Lijiang, Yunnan	KUN-L	*Lobaria pindarensis*
S. reticulata	**FXLiT2L1R5**	**OR343844**	**OR362899**	China	Lijiang, Yunnan	KUN-L	*Lobaria pindarensis*
S. reticulata	**FXLiT2L1R6**	**OR343845**	**OR362900**	China	Lijiang, Yunnan	KUN-L	*Lobaria pindarensis*
S. reticulata	**FXLiT2L1R7**	**OR343846**	**OR362901**	China	Lijiang, Yunnan	KUN-L	*Lobaria pindarensis*
S. reticulata	**FXLiT2L1R8**	**OR343847**	**OR362902**	China	Lijiang, Yunnan	KUN-L	*Lobaria pindarensis*
S. reticulata	**FXLiT2L1R9**		**OR362903**	China	Lijiang, Yunnan	KUN-L	*Lobaria pindarensis*
S. reticulata	**FXLiT3L1R1**	**OR343848**	**OR362904**	China	Lijiang, Yunnan	KUN-L	*Lobaria pindarensis*
S. reticulata	**FXLiT3L1R10**		**OR362905**	China	Lijiang, Yunnan	KUN-L	*Lobaria pindarensis*
S. reticulata	**FXLiT3L1R2**	**OR343849**	**OR362906**	China	Lijiang, Yunnan	KUN-L	*Lobaria pindarensis*
S. reticulata	**FXLiT3L1R3**	**OR343850**	**OR362907**	China	Lijiang, Yunnan	KUN-L	*Lobaria pindarensis*
S. reticulata	**FXLiT3L1R4**	**OR343851**	**OR362908**	China	Lijiang, Yunnan	KUN-L	*Lobaria pindarensis*
S. reticulata	**FXLiT3L1R6**	**OR343852**	**OR362910**	China	Lijiang, Yunnan	KUN-L	*Lobaria pindarensis*
S. reticulata	**FXLiT3L1R7**	**OR343853**	**OR362911**	China	Lijiang,	KUN-L	*Lobaria pindarensis*

					Yunnan		
S. reticulata	**FXLiT3L1R8**	**OR343854**	**OR362912**	China	Lijiang, Yunnan	KUN-L	*Lobaria pindarensis*
S. reticulata	**FXLiT3L1R9**	**OR343855**	**OR362913**	China	Lijiang, Yunnan	KUN-L	*Lobaria pindarensis*
S. reticulata	**FXLrT1L1R9a**		**OR362919**	China	Lijiang, Yunnan	KUN-L	*Lobaria isidiosa* group
S. reticulata	**HHAMLi1a**	**OR343863**		China	Honghe, Yunnan	KUN-L	*Lobaria* sp.1
S. reticulata	**HHAMLi2**	**OR343865**		China	Honghe, Yunnan	KUN-L	*Lobaria* sp.1
S. reticulata	**HHAMLr2a**		**OR362938**	China	Honghe, Yunnan	KUN-L	*Lobaria kurokawae*
S. reticulata	JF502554		JF502554				*Lobaria pulmonaria*
S. reticulata	**LJT1Li2**	**OR343867**		China	Lijiang, Yunnan	KUN-L	*Lobaria pindarensis*
S. reticulata	**LJT1Li3**	**OR343868**		China	Lijiang, Yunnan	KUN-L	*Lobaria pindarensis*
S. reticulata	**LJT1Li7**	**OR343869**		China	Lijiang, Yunnan	KUN-L	*Lobaria pindarensis*
S. reticulata	**LJT1Lk1**		**OR362939**	China	Lijiang, Yunnan	KUN-L	*Lobaria retigera*
S. reticulata	**LJT1Lk2**		**OR362940**	China	Lijiang, Yunnan	KUN-L	*Lobaria retigera*
S. reticulata	**LJT2Li3**	**OR343872**		China	Lijiang, Yunnan	KUN-L	*Lobaria pindarensis*
S. reticulata	**LJT2Li5**	**OR343873**		China	Lijiang, Yunnan	KUN-L	*Lobaria pindarensis*
S. reticulata	**LJT3Li1**	**OR343874**		China	Lijiang, Yunnan	KUN-L	*Lobaria pindarensis*
S. reticulata	**LJT3Li2**	**OR343875**		China	Lijiang, Yunnan	KUN-L	*Lobaria pindarensis*
S. reticulata	**LJT3Li3**	**OR343876**		China	Lijiang, Yunnan	KUN-L	*Lobaria* sp.
S. reticulata	**LJT3Li5**	**OR343877**		China	Lijiang, Yunnan	KUN-L	*Lobaria pindarensis*
S. reticulata	**LJT3Li6**	**OR343878**		China	Lijiang, Yunnan	KUN-L	*Lobaria pindarensis*

S. reticulata	SAG 5387	KC333466		Spain	Teneriffe		*Lobaria macaronesica*
Symbiochloris sp.	4677	MT314403	MT316667	Colombia			*Sticta sp.*
Symbiochloris sp.	4691	MT314417	MT316653	Colombia			*Sticta sp.*
Symbiochloris sp.	4704	MT314418	MT316668	Colombia			*Sticta sp.*
Symbiochloris sp.	6198	MT314421	MT316685	Colombia			*Sticta puracensis*
Symbiochloris sp.	7382	MT314405	MT316688	Ecuador			*Sticta aff. granatensis*
Symbiochloris sp.	AB06 006A2	KC333463	KC333577	Germany			*Lobaria pulmonaria*
Symbiochloris sp.	CCHU 5616	KC333476	KC333590	Japan			*Brigantiaea ferruginea*
Symbiochloris sp.	**FLrT1L1R2**		**OR362865**	China	Diqing, Yunnan	KUN-L	*Lobaria isidiosa* group
Symbiochloris sp.	**FLrT1L1R9**		**OR362870**	China	Diqing, Yunnan	KUN-L	*Lobaria isidiosa* group
Symbiochloris sp.	**FLrT2L1R2**		**OR362872**	China	Diqing, Yunnan	KUN-L	*Lobaria isidiosa* group
Symbiochloris sp.	**HHABLid1**		**OR362935**	China	Honghe, Yunnan	KUN-L	*Lobaria hengduanensis*
Symbiochloris sp.	**HHABLr1a**		**OR362936**	China	Honghe, Yunnan	KUN-L	*Lobaria hengduanensis*
Symbiochloris sp.	**HHABLr1b**		**OR362937**	China	Honghe, Yunnan	KUN-L	*Lobaria hengduanensis*
Symbiochloris sp.	UCONN4148	MT314360		Chile			*Sticta ainoae*
Symbiochloris tschermakiae	SCH 17084	KC333467	KC333571	Ecuador	Galapagos		*Lobaria patinifera*
Symbiochloris tschermakiae	SCH 6004	KC333462		USA	North Carolina		*Crocodia aurata*
Trebouxia aggregata	SAG 219-1d	EU123942.1	EU123967				
Trebouxia jamesii	UBT-86.132E2	Z68700					
Trebouxia angustilobata	SAG 2204		JF502557				
Trebouxia arboricola	SAG 219-1a	Z68705					
Trebouxia asymmetrica	Z21553	Z21553					*Diploschistes diacapsis*
Trebouxia australis	cort_4312		MT135880	USA	Eastern Cascades		*Melanohalea exasperatula*
Trebouxia corticola	UTEX 909		AB194847	Japan			
Trebouxia impressa	Z21551	Z21551					*Physcia stellaris*

Trebouxia magna	Z215521	Z21552					*Pilophorus acicaulis*	
Trebouxia sp.	142	KM056291		Italy	Torino		free-living	black patina on the rock surface of a gneiss quarry
Trebouxia sp.	AEW6BK241	KP081357		Germany			free-living	soil
Trebouxia sp.	AEW7RK112	KP081356		Germany			free-living	tree bark
Trebouxia sp.	B1-4-1E-144	JQ627431		Ireland			free-living	microbial biofilm on mineral composite substrate
Trebouxia sp.	FGSwa_K17	JX391012					free-living	marble monument
Trebouxia sp.	**FXLrT1L1R9b**	**OR343859**		China	Diqing, Yunnan	KUN-L	*Lobaria isidiosa* group	
Trebouxia sp.	GOGsM_K51	JX169846					free-living	plastic waste bin
Trebouxia sp.	**HHAMLi1b**	**OR343864**		China	Honghe, Yunnan	KUN-L	*Lobaria* sp. 1	
Trebouxia sp.	**HHAMLr2b**	**OR343866**		China	Honghe, Yunnan	KUN-L	*Lobaria hengduanensis*	
Trebouxia sp.	MPN676	MN563073					*Thamnolia sp.*	
Trebouxia sp.	RP2_6_1E_142	JQ627441					free-living	microbial biofilm on mineral composite substrate
Trebouxia sp.	UTEX SNO74	LC360463	LC360464				free-living	snow
Viridiella fridericiana	KR154344		KR154344	Italy	Campania		free-living	soil
Viridiella fridericiana	SAG 10.92	FM958481		UK				
**Outgroup**								
Ulva prolifera	LYG-HT	HQ850569		China				
Ulva prolifera	WTU 344778		AF499670					

Ulva rigida	EL0102	AJ005414		
Ulva rigida	UL051		KP233772	South Korea

(c)

Taxon	Specimen ID	GenBank number	Country	Region	Herbarium	Collector	Mycobiont
		16S					
Nostoc strain 1	EF174215	EF174215	Chile			A. Elvebakk, D. Papaefthimiou, E.H. Robertsen, A. Liaimer	*Pannaria aff. leproloma*
Nostoc strain 1	EF174218	EF174218	Chile	IX Región, Parque Nacional Conguillío		A. Elvebakk, D. Papaefthimiou, E.H. Robertsen, A. Liaimer	*Pannaria pallida*
Nostoc strain 1	EF174219	EF174219	Chile	X Región, Lago Llanquihue, Ensenada		A. Elvebakk, D. Papaefthimiou, E.H. Robertsen, A. Liaimer	*Pannaria tavaresii*
Nostoc strain 1	EF174220	EF174220	Chile			A. Elvebakk, D. Papaefthimiou, E.H. Robertsen, A. Liaimer	*Pannaria rubiginosa*
Nostoc strain 1	EF536021	EF536021	Chile	Archipiélago De Juan Fernández		A. Elvebakk, D. Papaefthimiou, E.H. Robertsen, A. Liaimer	*Pannaria andina*
Nostoc strain 1	**FXLiT3L1R5**	**OR335380**	China	Yunnan, Diqing	KUN-L	F.R. Worthy	*Lobaria* sp.
Nostoc strain 1	**HHABLi1**	**OR335403**	China	Yunnan, Honghe	KUN-L	F.R. Worthy	*Lobaria* sp.
Nostoc strain 1	**HHABLi2**	**OR335404**	China	Yunnan, Honghe	KUN-L	F.R. Worthy	*Lobaria* sp.
Nostoc strain 1	**HHABLid1**	**OR335405**	China	Yunnan, Honghe	KUN-L	F.R. Worthy	*Lobaria hengduanensis*
Nostoc strain 1	KF218	HQ591534	Finland			Fedrowitz et al	*Nephroma resupinatum*
Nostoc strain 1	LGR1008	KF704321	Reunion Island		LG	N. Magain, E. Serusiaux	*Pannaria rubiginosa*
Nostoc strain 1	LGR1011	KF704323	Reunion Island		LG	N. Magain, E. Serusiaux	*Pannaria rubiginosa*
Nostoc strain 2	LGR1123	KF704325	Reunion Island		LG	N. Magain	*Fuscopannaria leucosticta*
Nostoc strain 2	LGR1124	KF704353	Reunion Island		LG	N. Magain, E. Serusiaux	*Fuscopannaria leucosticta*
Nostoc strain 2	**LJT3Lr2**	**OR335412**	China	Yunnan, Lijiang	KUN-L	F.R. Worthy	*Lobaria kurokawae*
Nostoc strain 3	**FXLrT1L1R1**	**OR335381**	China	Yunnan, Diqing	KUN-L	F.R. Worthy	*Lobaria isidiosa* group
Nostoc strain 3	**LJT2Lk1**	**OR335408**	China	Yunnan, Lijiang	KUN-L	F.R. Worthy	*Lobaria retigera*
Nostoc strain 4	**DQT2Lr1**	**OR335359**	China	Yunnan, Diqing	KUN-L	F.R. Worthy	*Lobaria retigera*
Nostoc strain 4	**FLrT1L1R6**	**OR335365**	China	Yunnan, Diqing	KUN-L	F.R. Worthy	*Lobaria retigera*
Nostoc strain 4	**FLrT2L1R8**	**OR335372**	China	Yunnan, Diqing	KUN-L	F.R. Worthy	*Lobaria retigera*

Nostoc strain 4	**FLrT3L1R10**	**OR335374**	China	Yunnan, Diqing	KUN-L	F.R. Worthy	*Lobaria isidiosa* group
Nostoc strain 4	**FLrT3L1R2**	**OR335375**	China	Yunnan, Diqing	KUN-L	F.R. Worthy	*Lobaria isidiosa* group
Nostoc strain 4	**FLrT3L1R5**	**OR335377**	China	Yunnan, Diqing	KUN-L	F.R. Worthy	*Lobaria latilobulata*
Nostoc strain 4	**FXLrT1L1R3**	**OR335382**	China	Yunnan, Diqing	KUN-L	F.R. Worthy	*Lobaria retigera*
Nostoc strain 4	**FXLrT1L1R4**	**OR335383**	China	Yunnan, Diqing	KUN-L	F.R. Worthy	*Lobaria retigera*
Nostoc strain 4	**FXLrT1L1R7**	**OR335385**	China	Yunnan, Diqing	KUN-L	F.R. Worthy	*Lobaria retigera*
Nostoc strain 4	**FXLrT1L1R8**	**OR335386**	China	Yunnan, Diqing	KUN-L	F.R. Worthy	*Lobaria retigera*
Nostoc strain 4	**FXLrT2L1R10**	**OR335388**	China	Yunnan, Diqing	KUN-L	F.R. Worthy	*Lobaria isidiosa* group
Nostoc strain 4	**FXLrT2L1R6**	**OR335392**	China	Yunnan, Diqing	KUN-L	F.R. Worthy	*Lobaria isidiosa* group
Nostoc strain 4	**FXLrT2L1R8**	**OR335394**	China	Yunnan, Diqing	KUN-L	F.R. Worthy	*Lobaria isidiosa* group
Nostoc strain 4	**LJT1Lk1**	**OR335407**	China	Yunnan, Lijiang	KUN-L	F.R. Worthy	*Lobaria retigera*
Nostoc strain 4	**LJT2Lr6**	**OR335409**	China	Yunnan, Lijiang	KUN-L	F.R. Worthy	*Lobaria* sp.
Nostoc strain 5	661	DQ185236	Austria		H	E. Hansen	*Nephroma bellum*
Nostoc strain 5	03005	AY333618	Finland		H	Rikkinen *et al.*	*Nephroma parile*
Nostoc strain 5	13239	AY333621	Canada	SW	H	Vitikainen	*Nephroma bellum*
Nostoc strain 5	13242	AY333619	Canada	SW	H	Vitikainen	*Nephroma parile*
Nostoc strain 5	AF506253	AF506253	Canada			J. Rikkinen, I. Oksanen, K. Lohtander	*Nephroma parile*
Nostoc strain 5	AF506255	AF506255	Finland			J. Rikkinen, I. Oksanen, K. Lohtander	*Parmeliella triptophylla*
Nostoc strain 5	AF506257	AF506257	Finland			J. Rikkinen, I. Oksanen, K. Lohtander	*Nephroma parile*
Nostoc strain 5	**FLrT1L1R1**	**OR335360**	China	Yunnan, Diqing	KUN-L	F.R. Worthy	*Lobaria isidiosa* group
Nostoc strain 5	**FLrT1L1R10**	**OR335361**	China	Yunnan, Diqing	KUN-L	F.R. Worthy	*Lobaria isidiosa* group
Nostoc strain 5	**FLrT1L1R2**	**OR335362**	China	Yunnan, Diqing	KUN-L	F.R. Worthy	*Lobaria isidiosa* group
Nostoc strain 5	**FLrT1L1R4**	**OR335363**	China	Yunnan, Diqing	KUN-L	F.R. Worthy	*Lobaria isidiosa* group
Nostoc strain 5	**FLrT1L1R5**	**OR335364**	China	Yunnan, Diqing	KUN-L	F.R. Worthy	*Lobaria isidiosa* group
Nostoc strain 5	**FLrT1L1R7**	**OR335366**	China	Yunnan, Diqing	KUN-L	F.R. Worthy	*Lobaria retigera*
Nostoc strain 5	**FLrT1L1R9**	**OR335367**	China	Yunnan, Diqing	KUN-L	F.R. Worthy	*Lobaria isidiosa* group
Nostoc strain 5	**FLrT2L1R1**	**OR335368**	China	Yunnan, Diqing	KUN-L	F.R. Worthy	*Lobaria isidiosa* group
Nostoc strain 5	**FLrT2L1R2**	**OR335369**	China	Yunnan, Diqing	KUN-L	F.R. Worthy	*Lobaria isidiosa* group
Nostoc strain 5	**FLrT2L1R4**	**OR335370**	China	Yunnan, Diqing	KUN-L	F.R. Worthy	*Lobaria isidiosa* group

Nostoc strain 5	**FLrT2L1R6**	**OR335371**	China	Yunnan, Diqing	KUN-L	F.R. Worthy	*Lobaria retigera*
Nostoc strain 5	**FLrT3L1R1**	**OR335373**	China	Yunnan, Diqing	KUN-L	F.R. Worthy	*Lobaria retigera*
Nostoc strain 5	**FLrT3L1R3**	**OR335376**	China	Yunnan, Diqing	KUN-L	F.R. Worthy	*Lobaria isidiosa* group
Nostoc strain 5	**FLrT3L1R7**	**OR335378**	China	Yunnan, Diqing	KUN-L	F.R. Worthy	*Lobaria isidiosa* group
Nostoc strain 5	**FLrT3L1R9**	**OR335379**	China	Yunnan, Diqing	KUN-L	F.R. Worthy	*Lobaria isidiosa* group
Nostoc strain 5	**FXLrT1L1R6**	**OR335384**	China	Yunnan, Diqing	KUN-L	F.R. Worthy	*Lobaria isidiosa* group
Nostoc strain 5	**FXLrT1L1R9**	**OR335387**	China	Yunnan, Diqing	KUN-L	F.R. Worthy	*Lobaria isidiosa* group
Nostoc strain 5	**FXLrT2L1R3**	**OR335389**	China	Yunnan, Diqing	KUN-L	F.R. Worthy	*Lobaria isidiosa* group
Nostoc strain 5	**FXLrT2L1R4**	**OR335390**	China	Yunnan, Diqing	KUN-L	F.R. Worthy	*Lobaria isidiosa* group
Nostoc strain 5	**FXLrT2L1R5**	**OR335391**	China	Yunnan, Diqing	KUN-L	F.R. Worthy	*Lobaria isidiosa* group
Nostoc strain 5	**FXLrT2L1R7**	**OR335393**	China	Yunnan, Diqing	KUN-L	F.R. Worthy	*Lobaria isidiosa* group
Nostoc strain 5	**FXLrT2L1R9**	**OR335395**	China	Yunnan, Diqing	KUN-L	F.R. Worthy	*Lobaria isidiosa* group
Nostoc strain 5	**FXLrT3L1R1**	**OR335396**	China	Yunnan, Diqing	KUN-L	F.R. Worthy	*Lobaria isidiosa* group
Nostoc strain 5	**FXLrT3L1R10**	**OR335397**	China	Yunnan, Diqing	KUN-L	F.R. Worthy	*Lobaria isidiosa* group
Nostoc strain 5	**FXLrT3L1R2**	**OR335398**	China	Yunnan, Diqing	KUN-L	F.R. Worthy	*Lobaria isidiosa* group
Nostoc strain 5	**FXLrT3L1R3**	**OR335399**	China	Yunnan, Diqing	KUN-L	F.R. Worthy	*Lobaria isidiosa* group
Nostoc strain 5	**FXLrT3L1R8**	**OR335401**	China	Yunnan, Diqing	KUN-L	F.R. Worthy	*Lobaria isidiosa* group
Nostoc strain 5	**FXLrT3L1R9**	**OR335402**	China	Yunnan, Diqing	KUN-L	F.R. Worthy	*Lobaria isidiosa* group
Nostoc strain 5	KF10	HQ591509	Finland			Fedrowitz et al	*Nephroma bellum*
Nostoc strain 5	KF139	HQ591520	Finland			Fedrowitz et al	*Nephroma parile*
Nostoc strain 5	KF17	HQ591510	Finland			Fedrowitz et al	*Nephroma bellum*
Nostoc strain 5	KF188	HQ591527	Finland			Fedrowitz et al	*Nephroma resupinatum*
Nostoc strain 5	KF199	HQ591531	Finland			Fedrowitz et al	*Nephroma resupinatum*
Nostoc strain 5	KF216	HQ591533	Finland			Fedrowitz et al	*Nephroma resupinatum*
Nostoc strain 5	KF224	HQ591529	Finland			Fedrowitz et al	*Nephroma resupinatum*
Nostoc strain 5	KF233	HQ591532	Finland			Fedrowitz et al	*Nephroma resupinatum*
Nostoc strain 5	KF33	HQ591513	Finland			Fedrowitz et al	*Nephroma bellum*
Nostoc strain 5	KF54	HQ591516	Finland			Fedrowitz et al	*Nephroma bellum*
Nostoc strain 5	KF71B	HQ591514	Finland			Fedrowitz et al	*Nephroma bellum*
Nostoc strain 5	**LJT3Lk1**	**OR335410**	China	Yunnan, Lijiang	KUN-L	F.R. Worthy	*Lobaria isidiosa* group
Nostoc strain 5	**LJT3Lr1**	**OR335411**	China	Yunnan, Lijiang	KUN-L	F.R. Worthy	*Lobaria isidiosa* group

Nostoc strain 5	Lpul376b	EF102243	Finland	Evo	H	Myllys, Kuusinen	*Lobaria pulmonaria*
Nostoc strain 5	Lpul385	EF102244	Finland	Evo	H	Myllys, Kuusinen	*Lobaria pulmonaria*
Nostoc strain 5	Lpul391	EF102245	Finland	Evo	H	Myllys, Kuusinen	*Lobaria pulmonaria*
Nostoc strain 5	Lpul431	EF102249	Finland	Myrskylä	H	Myllys, Kuusinen	*Lobaria pulmonaria*
Nostoc strain 5	Nbel21173	EF102257	Finland	Evo	H	Pykälä	*Nephroma bellum*
Nostoc strain 5	Nbel21173b	EF102257	Finland	Evo	H	Pykälä	*Nephroma bellum*
Nostoc strain 5	Nbel383	EF102253	Finland	Evo	H	Myllys, Kuusinen	*Nephroma bellum*
Nostoc strain 5	Nbel393	EF102255	Finland	Evo	H	Myllys, Kuusinen	*Nephroma bellum*
Nostoc strain 5	Nbel415	EF102256	Finland	Evo	H	Myllys, Kuusinen	*Nephroma bellum*
Nostoc strain 5	Npar386	EF102262	Finland	Evo	H	Myllys, Kuusinen	*Nephroma parile*
Nostoc strain 5	Npar402	EF102263	Finland	Evo	H	Myllys, Kuusinen	*Nephroma parile*
Nostoc strain 5	Npar416	EF102264	Finland	Evo	H	Myllys, Kuusinen	*Nephroma parile*
Nostoc strain 5	Npar416	EF102256	Finland	Evo	H	Myllys, Kuusinen	*Nephroma parile*
Nostoc strain 5	Nres19573	EF102266	Finland	Lohia	H	Pykälä	*Nephroma resupinatum*
Nostoc strain 5	Ptri387	EF102269	Finland	Evo	H	Myllys, Kuusinen	*Parmeliella triptophylla*
Nostoc strain 5	UK105	HQ591519				Fedrowitz et al	*Nephroma parile*
Nostoc strain 5	UK108	HQ591530				Fedrowitz et al	*Nephroma bellum*
Nostoc strain 5	UK110	HQ591511				Fedrowitz et al	*Nephroma resupinatum*
Nostoc strain 5	UK119	HQ591512				Fedrowitz et al	*Nephroma bellum*
Nostoc strain 5	UK371	JN847360	Norway	Vestfold		Fedrowitz *et al.*	*Nephroma parile*
Nostoc strain 6	**HHABLr3**	**OR335406**	China	Yunnan, Honghe	KUN-L	F.R. Worthy	*Lobaria* sp.
Nostoc strain 7	**FXLrT3L1R6**	**OR335400**	China	Yunnan, Diqing	KUN-L	F.R. Worthy	*Lobaria isidiosa* group
Nostoc sp.	25Nash	AY333616	USA	SW	H	Nash III	*Nephroma resupinatum*
Nostoc sp.	26Nash	AY333617	USA	SW	H	Nash III	*Nephroma bellum*
Nostoc sp.	EF174222	EF174222	New Zealand	Northland		A. Elvebakk, D. Papaefthimiou, E.H. Robertsen, A. Liaimer	*Pannaria araneosa*
Nostoc sp.	EF536023	EF536023	Chile	Archipiélago De Juan Fernández		A. Elvebakk, D. Papaefthimiou, E.H. Robertsen, A. Liaimer	*Pseudocyphellaria imshaugii*
Nostoc sp.	KF91B	HQ591518	Finland			Fedrowitz et al	*Nephroma bellum*
Nostoc sp.	KT884013	KT884013	Reunion Island			N. Magain, E. Serusiaux	*Fuscopannaria ahlneri*
Nostoc sp.	LGR1021	KF704327	Reunion island		LG	N. Magain, E. Serusiaux	*Parmeliella polyphyllina*

Nostoc sp.	LGR1058	KF704326	Reunion island		LG	N. Magain, E. Serusiaux	*Parmeliella polyphyllina*
Nostoc sp.	Nbel19679	EF102258	Finland	Lohia	H	Pykälä	*Nephroma bellum*
Nostoc sp.	Nbel388	EF102254	Finland	Evo	H	Myllys, Kuusinen	*Nephroma bellum*
Nostoc sp.	SS5087	DQ265924	Brazil	Campos do Jordao	TUR	S. Stenroos	*Pseudocyphellaria clathrata*
Nostoc sp.	UK368	JN847358	Norway	Vestfold		S. Olsson, U. Kaasalainen, J. Rikkinen	*Lobaria virens*
**Outgroup**							
Nostoc commune	Y12687	Y12687	China			J. Rikkinen, I. Oksanen, K. Lohtander	

Table S3

List of (a) chlorolichen and (b) chloro-cyanolichen specimens used in this study. Green algae were observed in cross-sections of all these specimens, but in some cases no high-quality sequence was obtained (marked as ~). Identities of *Lobaria* mycobionts and their associated green algal and cyanobacterial photobionts were based on phylogenetic analyses of gene sequence data. Accession numbers are provided in Table S2. Study sites were in Diqing (DQ), Lijiang (LJ) and Honghe (HH) prefectures.

(a)

Specimen ID	Site	Elevation (m)	Mycobiont	Green algae	
			ITS-EF-1α-RPB	*18S*	*RBC-L*
DQT1Li2	DQ	3555	*Lobaria costata*	*Symbiochloris reticulata*	~
FXLiT1L1R4	LJ	3447	*L. perelegans*	*S. reticulata*	*S. reticulata*
LJT2Li1	LJ	3450	*L. pindarensis*	~	~
FXLiT3L1R10	LJ	3393	*L. pindarensis*	~	*S. reticulata*
LJT1Li1	LJ	3445	*L. pindarensis*	~	~
FXLiT2L1R7	LJ	3351	*L. pindarensis*	*S. reticulata*	*S. reticulata*
LJT2Li5	LJ	3487	*L. pindarensis*	*S. reticulata*	~
LJT3Li2	LJ	3163	*L. pindarensis*	*S. reticulata*	~
LJT3Li5	LJ	3407	*L. pindarensis*	*S. reticulata*	~
LJT3Li6	LJ	3409	*L. pindarensis*	*S. reticulata*	~
DQT1Li1	DQ	3922	*L. pindarensis*	*S. reticulata*	~
DQT3Li1	DQ	3995	*L. pindarensis*	*S. reticulata*	~
FXLiT1L1R10	LJ	3447	*L. pindarensis*	*S. reticulata*	*S. reticulata*
FXLiT1L1R3	LJ	3447	*L. pindarensis*	*S. reticulata*	*S. reticulata*
FXLiT1L1R5	LJ	3447	*L. pindarensis*	*S. reticulata*	*S. reticulata*
FXLiT1L1R9	LJ	3447	*L. pindarensis*	*S. reticulata*	*S. reticulata*
FXLiT2L1R1	LJ	3351	*L. pindarensis*	*S. reticulata*	*S. reticulata*
FXLiT2L1R10	LJ	3351	*L. pindarensis*	*S. reticulata*	*S. reticulata*
FXLiT2L1R2	LJ	3351	*L. pindarensis*	*S. reticulata*	*S. reticulata*
FXLiT2L1R3	LJ	3351	*L. pindarensis*	*S. reticulata*	*S. reticulata*

FXLiT2L1R4a&b	LJ	3351	*L. pindarensis*	*S. reticulata*	*S. reticulata*
FXLiT2L1R5	LJ	3351	*L. pindarensis*	*S. reticulata*	*S. reticulata*
FXLiT2L1R6	LJ	3351	*L. pindarensis*	*S. reticulata*	*S. reticulata*
FXLiT2L1R8	LJ	3351	*L. pindarensis*	*S. reticulata*	*S. reticulata*
FXLiT2L1R9	LJ	3351	*L. pindarensis*	*S. reticulata*	*S. reticulata*
FXLiT3L1R1	LJ	3393	*L. pindarensis*	*S. reticulata*	*S. reticulata*
FXLiT3L1R2	LJ	3393	*L. pindarensis*	*S. reticulata*	*S. reticulata*
FXLiT3L1R3	LJ	3393	*L. pindarensis*	*S. reticulata*	*S. reticulata*
FXLiT3L1R4	LJ	3393	*L. pindarensis*	*S. reticulata*	*S. reticulata*
FXLiT3L1R6	LJ	3393	*L. pindarensis*	*S. reticulata*	*S. reticulata*
FXLiT3L1R7	LJ	3393	*L. pindarensis*	*S. reticulata*	*S. reticulata*
FXLiT3L1R8	LJ	3393	*L. pindarensis*	*S. reticulata*	*S. reticulata*
FXLiT3L1R9	LJ	3393	*L. pindarensis*	*S. reticulata*	*S. reticulata*
LJT1Li2	LJ	3406	*L. pindarensis*	*S. reticulata*	~
LJT1Li3	LJ	3409	*L. pindarensis*	*S. reticulata*	~
LJT1Li7	LJ	3211	*L. pindarensis*	*S. reticulata*	~
LJT2Li3	LJ	3489	*L. pindarensis*	*S. reticulata*	~
LJT3Li1	LJ	3177	*L. pindarensis*	*S. reticulata*	~
DQT2Li2a&b	DQ	3713	*L. pindarensis*	*S. reticulata*	~
DQT2Li1	DQ	3700	*Lobaria* sp.	~	*S. reticulata*
LJT3Li3	LJ	3163	*Lobaria* sp.	*S. reticulata*	~
HHAMLi2	HH	2150	*Lobaria* sp. 1	*S. reticulata*	~
HHAMLi1a&b	HH	2111	*Lobaria* sp. 1	*Trebouxia* & *S. reticulata*	
FXLiT1L1R1	LJ	3447	*Lobaria* sp. 2	*S. reticulata*	*S. reticulata*
FXLiT1L1R2	LJ	3447	*Lobaria* sp. 2	*S. reticulata*	*S. reticulata*
FXLiT1L1R6	LJ	3447	*Lobaria* sp. 2	*S. reticulata*	*S. reticulata*
FXLiT1L1R7	LJ	3447	*Lobaria* sp. 2	*S. reticulata*	*S. reticulata*
FXLiT1L1R8	LJ	3447	*Lobaria* sp. 2	*S. reticulata*	*S. reticulata*

(b)

Specimen ID	Site	Elevation (m)	Mycobiont	Green algae		Cyanobiont
			ITS-EF-1α-RPB	*18S*	*RBC-L*	*16S*
HHAMLr1	HH	2150	*L. hengduanensis*	~	~	*Nostoc* sp.

HHAMLr3	HH	2100	*L. hengduanensis*	~	~	*Nostoc* sp.
HHABLr1a&b	HH	2142	*L. hengduanensis*	~	*Symbiochloris* sp.	*Nostoc* sp.
HHABLid1	HH	2142	*L. hengduanensis*	~	*Symbiochloris* sp.	*Nostoc* strain 1
FLrT2L1R10	DQ	3847	*L. isidiosa* group	~	~	*Nostoc* sp.
FXLrT1L1R10	DQ	3964	*L. isidiosa* group	~	~	*Nostoc* sp.
FXLrT3L1R7	DQ	4045	*L. isidiosa* group	~	~	*Nostoc* sp.
FXLrT1L1R5	DQ	3964	*L. isidiosa* group	*Parachloroidium* sp.	~	*Nostoc* sp.
DQT2Lr1	DQ	3882	*L. isidiosa* group	*Parachloroidium* sp.	~	*Nostoc* strain 4
FLrT1L1R10	DQ	3964	*L. isidiosa* group	~	~	*Nostoc* strain 5
FXLrT2L1R4	DQ	3847	*L. isidiosa* group	~	~	*Nostoc* strain 5
LJT3Lk1	LJ	3404	*L. isidiosa* group	~	~	*Nostoc* strain 5
LJT3Lr1	LJ	3178	*L. isidiosa* group	~	~	*Nostoc* strain 5
FXLrT3L1R6	DQ	4045	*L. isidiosa* group	~	~	*Nostoc* strain 7
FXLrT2L1R2	DQ	3847	*L. isidiosa* group	~	*Parachloroidium* sp.	*Nostoc* sp.
FXLrT3L1R5	DQ	4045	*L. isidiosa* group	~	*Parachloroidium* sp.	*Nostoc* sp.
FXLrT1L1R1	DQ	3964	*L. isidiosa* group	~	*Parachloroidium* sp.	*Nostoc* strain 3
FLrT3L1R10	DQ	4045	*L. isidiosa* group	~	*Parachloroidium* sp.	*Nostoc* strain 4
FLrT3L1R2	DQ	4045	*L. isidiosa* group	~	*Parachloroidium* sp.	*Nostoc* strain 4
FXLrT2L1R10	DQ	3847	*L. isidiosa* group	~	*Parachloroidium* sp.	*Nostoc* strain 4
FXLrT2L1R6	DQ	3847	*L. isidiosa* group	~	*Parachloroidium* sp.	*Nostoc* strain 4
FXLrT2L1R8	DQ	3847	*L. isidiosa* group	*Parachloroidium* sp.	*Parachloroidium* sp.	*Nostoc* strain 4
FLrT1L1R4	DQ	3964	*L. isidiosa* group	~	*Parachloroidium* sp.	*Nostoc* strain 5
FLrT1L1R5	DQ	3964	*L. isidiosa* group	~	*Parachloroidium* sp.	*Nostoc* strain 5
FLrT3L1R3	DQ	4045	*L. isidiosa* group	~	*Parachloroidium* sp.	*Nostoc* strain 5
FLrT3L1R7	DQ	4045	*L. isidiosa* group	~	*Parachloroidium* sp.	*Nostoc* strain 5
FLrT3L1R9	DQ	4045	*L. isidiosa* group	~	*Parachloroidium* sp.	*Nostoc* strain 5
FXLrT1L1R6	DQ	3964	*L. isidiosa* group	~	*Parachloroidium* sp.	*Nostoc* strain 5
FXLrT2L1R3	DQ	3847	*L. isidiosa* group	~	*Parachloroidium* sp.	*Nostoc* strain 5
FXLrT2L1R5	DQ	3847	*L. isidiosa* group	~	*Parachloroidium* sp.	*Nostoc* strain 5
FXLrT2L1R7	DQ	3847	*L. isidiosa* group	~	*Parachloroidium* sp.	*Nostoc* strain 5
FXLrT2L1R9	DQ	3847	*L. isidiosa* group	~	*Parachloroidium* sp.	*Nostoc* strain 5
FXLrT3L1R1	DQ	4045	*L. isidiosa* group	~	*Parachloroidium* sp.	*Nostoc* strain 5
FXLrT3L1R10	DQ	4045	*L. isidiosa* group	~	*Parachloroidium* sp.	*Nostoc* strain 5
FXLrT3L1R2	DQ	4045	*L. isidiosa* group	~	*Parachloroidium* sp.	*Nostoc* strain 5
FXLrT3L1R3	DQ	4045	*L. isidiosa* group	~	*Parachloroidium* sp.	*Nostoc* strain 5
FXLrT3L1R8	DQ	4045	*L. isidiosa* group	~	*Parachloroidium* sp.	*Nostoc* strain 5

FLrT2L1R1	DQ	3847	*L. isidiosa* group	*Parachloroidium* sp.	*Parachloroidium* sp.	*Nostoc* strain 5
FLrT2L1R4	DQ	3847	*L. isidiosa* group	*Parachloroidium* sp.	*Parachloroidium* sp.	*Nostoc* strain 5
FXLrT3L1R9	DQ	4045	*L. isidiosa* group	*Parachloroidium* sp.	*Parachloroidium* sp.	*Nostoc* strain 5
FLrT1L1R1	DQ	3964	*L. isidiosa* group	*Pseudochlorella* sp.	*Parachloroidium* sp.	*Nostoc* strain 5
FLrT1L1R2	DQ	3964	*L. isidiosa* group	~	*Symbiochloris* sp.	*Nostoc* strain 5
FLrT1L1R9	DQ	3964	*L. isidiosa* group	~	*Symbiochloris* sp.	*Nostoc* strain 5
FLrT2L1R2	DQ	3847	*L. isidiosa* group	~	*Symbiochloris* sp.	*Nostoc* strain 5
FXLrT1L1R9	DQ	3964	*L. isidiosa* group	*Trebouxia* sp.	*S. reticulata*	*Nostoc* strain 5
LJT2Li2	LJ	3476	*L. kurokawae*	*Parachloroidium* sp.	~	*Nostoc* sp.
LJT3Lr2	LJ	3401	*L. kurokawae*	~	~	*Nostoc* strain 2
HHAMLr2	HH	2044	*L. kurokawae*	*Trebouxia* sp.	*S. reticulata*	*Nostoc* sp.
DQT2Lr2	DQ	3756	*L. latilobulata*	~	~	*Nostoc* sp.
FLrT3L1R5	DQ	4045	*L. latilobulata*	*Parachloroidium* sp.	*Parachloroidium* sp.	*Nostoc* strain 4
FLrT3L1R4	DQ	4045	*L. retigera* 1	~	~	*Nostoc* sp.
FLrT3L1R6	DQ	4045	*L. retigera* 1	~	~	*Nostoc* sp.
LJT2Lk1	LJ	3486	*L. retigera* 1	~	~	*Nostoc* strain 3
FXLrT1L1R3	DQ	3964	*L. retigera* 1	*Chloroidium* sp.	~	*Nostoc* strain 4
FLrT1L1R6	DQ	3964	*L. retigera* 1	~	*Parachloroidium* sp.	*Nostoc* strain 4
FXLrT1L1R4	DQ	3964	*L. retigera* 1	~	*Parachloroidium* sp.	*Nostoc* strain 4
FXLrT1L1R8	DQ	3964	*L. retigera* 1	~	*Parachloroidium* sp.	*Nostoc* strain 4
FLrT3L1R1	DQ	4045	*L. retigera* 1	~	*Parachloroidium* sp.	*Nostoc* strain 5
FLrT1L1R7	DQ	3964	*L. retigera* 1	*Parachloroidium* sp.	*Parachloroidium* sp.	*Nostoc* strain 5
LJT1Lk2	LJ	3413	*L. retigera* 1	~	*S. reticulata*	*Nostoc* sp.
DQT3Lr1	DQ	3933	*L. retigera* 2	~	~	*Nostoc* sp.
FXLrT2L1R1	DQ	3847	*L. retigera* 2	*Pseudochlorella* sp.	~	*Nostoc* sp.
LJT1Lr1	LJ	3439	*L. retigera* 2	*Pseudochlorella* sp.	~	*Nostoc* sp.
FXLrT1L1R7	DQ	3964	*L. retigera* 2	*Apatococcus* sp.	*Parachloroidium* sp.	*Nostoc* strain 4
FLrT2L1R8	DQ	3847	*L. retigera* 2	~	*Parachloroidium* sp.	*Nostoc* strain 4
FLrT2L1R6	DQ	3847	*L. retigera* 2	~	*Parachloroidium* sp.	*Nostoc* strain 5
LJT1Lk1	LJ	3419	*L. retigera* 2	~	*S. reticulata*	*Nostoc* strain 4
FLrT2L1R5	DQ	3847	*Lobaria* sp.	~	~	*Nostoc* sp.
DQT1Lr1	DQ	3964	*Lobaria* sp.	*Parachloroidium* sp.	~	*Nostoc* sp.
HHABLr3	HH	2140	*Lobaria* sp.	~	~	*Nostoc* strain 6
FXLiT3L1R5	DQ	3393	*Lobaria* sp.	~	*Parachloroidium* sp.	*Nostoc* strain 1
LJT2Lr6	LJ	3195	*Lobaria* sp.	~	~	*Nostoc* strain 4

27

Table S4. Bartlett's K^2 test for difference in the variance of chlorophyll content (µg mL^{-1}) of *Lobaria* spp. specimens collected along an elevational gradient, according to their morphotype, mycobiont and photobionts.

**Chlorophyll *a***			**Chlorophyll *b***			**Chlorophyll *a/b* ratio**		
K^2	d.f.	*P*	K^2	d.f.	*P*	K^2	d.f.	*P*
Morphotype × elevation			Morphotype × elevation			Morphotype × elevation		
89.4	36	**<0.0001**	103	36	**<0.0001**	74.2	36	**0.0002**
Lobaria spp.× elevation			*Lobaria* spp. × elevation			*Lobaria* spp. × elevation		
88.7	37	**<0.0001**	102	37	**<0.0001**	75.7	37	**0.0002**
Green algae × elevation			Green algae × elevation			Green algae × elevation		
90.1	37	**<0.0001**	102	37	**<0.0001**	75.6	37	**0.0002**
For the chloro-cyanolichen morphotype								
Nostoc × elevation			*Nostoc* × elevation			*Nostoc* × elevation		
1.08	4	**0.89**	5.87	4	**0.21**	3.43	4	**0.49**

Table S5. Linear model for CO_2 exchange rate (μmol CO_2 min^{-1} g^{-1}) of *Lobaria* specimens (mycobionts LA to SN2) exposed to 33% humidity conditions. The chlorolichens were *L. pindarensis* (PI), *L. perelegans* (PE) and *Lobaria* sp. 2 (SN2). The chloro-cyanolichens were *L. isidiosa*, *L. latilobulata* (LA) and *L. retigera* (RT).

**Net photosynthetic rate**				
All specimens				
	Est.	S.E.	*t*	*P*
Lobaria				
(Intercept)	-0.001	0.001	-1.02	0.31
L. latilobulata (LA)	0.003	0.003	0.90	0.37
L. perelegans (PE)	-0.0002	0.002	-0.09	0.93
L. pindarensis (PI)	0.001	0.001	0.61	0.54
L. retigera (RT)	0.002	0.003	0.75	0.46
Lobaria 2 (SN2)	0.001	0.002	0.73	0.47
**Green algae**				
(Intercept)	0.002	0.002	1.22	0.22
Parachloroidium (PC)	-0.003	0.002	-1.60	0.11
S. reticulata (SR)	-0.003	0.002	-1.28	0.20

**Gross photosynthetic rate**				
All specimens				
	Est.	S.E.	*t*	*P*
Lobaria				
(Intercept)	0.007	0.0005	16.30	< **0.0001**
LA	0.001	0.002	0.39	0.69
PE	-0.005	0.002	-3.08	**0.002**
PI	-0.001	0.001	-1.54	0.12
RT	0.004	0.001	3.43	< **0.001**
SN2	-0.002	0.001	-1.99	0.05
**Green algae**				
(Intercept)	0.006	0.001	4.40	< **0.0001**
PC	0.003	0.001	2.04	0.04
SR	0.001	0.001	0.48	0.63

**Dark respiration**				
All specimens				
	Est.	S.E.	*t*	*P*
Lobaria				
(Intercept)	0.002	0.001	2.21	0.03
LA	-0.003	0.003	-0.89	0.37
PE	0.002	0.002	1.12	0.27
PI	-0.001	0.001	-0.90	0.37
RT	-0.001	0.003	-0.50	0.62
SN2	0.0001	0.001	0.05	0.96
**Green algae**				
(Intercept)	0.003	0.002	1.42	0.16
PC	-0.001	0.002	-0.72	0.48
SR	-0.001	0.002	-0.78	0.44

Specimens with green algae *S. reticulata*				
(Intercept)	-0.001	0.002	-0.50	0.62
PI	0.001	0.002	0.37	0.71
SN2	0.001	0.003	0.55	0.58

Specimens with green algae *Parachloroidium*				
(Intercept)	-0.001	0.001	-1.70	0.10
LA	0.004	0.003	1.13	0.27
RT	0.003	0.003	0.97	0.34

L. isidiosa with green algae *Parachloroidium*				
(Intercept)	0.001	0.001	0.41	0.68
Nostoc clade 5 (N5)	-0.003	0.002	-1.75	0.09

Specimens with green algae *S. reticulata*				
(Intercept)	0.003	0.002	1.68	0.10
PI	0.001	0.002	0.38	0.71
SN2	0.0005	0.002	0.24	0.81

Specimens with green algae *Parachloroidium*				
(Intercept)	0.003	0.001	5.67	< **0.0001**
LA	-0.0002	0.002	-0.10	0.92
RT	-0.002	0.002	-0.73	0.47

L. isidiosa with green algae *Parachloroidium*				
(Intercept)	0.003	0.001	2.99	**0.006**
N5	0.0003	0.001	0.21	0.84

Specimens with green algae *S. reticulata*				
(Intercept)	0.004	0.002	2.22	0.03
PI	-0.003	0.002	-1.68	0.10
SN2	-0.002	0.002	-1.01	0.32

Specimens with green algae *Parachloroidium*				
(Intercept)	0.001	0.001	1.47	0.15
LA	-0.002	0.004	-0.68	0.50
RT	-0.001	0.004	-0.36	0.72

L. isidiosa with green algae *Parachloroidium*				
(Intercept)	-0.002	0.001	-1.37	0.18
N5	0.005	0.002	2.86	**0.009**

Table S6. Dynamic linear model for CO_2 exchange rate (µmol CO_2 min^{-1} g^{-1}) of *Lobaria* specimens (mycobionts LA to SN2). The linear time series is expressed according to either progressively increasing humidity conditions alone (humidity) or by the eight steps of the experiment (step). The chlorolichens were *L. pindarensis* (PI), *L. perelegans* (PE) and *Lobaria* sp. 2. (SN2). The chloro-cyanolichens were *L. isidiosa, L. latilobulata* (LA) and *L. retigera* (RT).

**Net photosynthetic rate**				
All specimens				
	Est.	S.E.	*t*	*P*
Lobaria				
(Intercept)	-0.005	0.002	-2.27	0.02
L. latilobulata (LA)	0.008	0.008	1.02	0.31
L. perelegans (PE)	0.002	0.007	0.33	0.74
L. pindarensis (PI)	0.007	0.003	2.53	0.012
L. retigera (RT)	0.001	0.006	0.19	0.85
Lobaria 2 (SN2)	0.012	0.004	2.69	**0.01**
step	-0.001	0.0004	-1.99	0.05
LA × step	-0.0003	0.002	-0.20	0.84
PE × step	0.001	0.001	0.59	0.56
PI × step	-0.0002	0.001	-0.32	0.75
RT × step	0.001	0.001	0.46	0.65
SN2 × step	-0.002	0.001	-1.86	0.06
**Green algae**				
(Intercept)	0.009	0.003	2.87	**0.004**
Parachloroidium (PC)	-0.008	0.003	-2.95	**0.003**

**Gross photosynthetic rate**				
All specimens				
	Est.	S.E.	*t*	*P*
Lobaria				
(Intercept)	0.006	0.001	6.08	**<0.0001**
LA	-0.003	0.004	-0.73	0.47
PE	-0.005	0.003	-1.46	0.14
PI	-0.004	0.001	-3.10	**0.002**
RT	0.000	0.003	0.14	0.89
SN2	-0.007	0.002	-3.31	**0.001**
step	0.0003	0.0002	1.23	0.22
LA × step	0.001	0.001	0.97	0.33
PE × step	0.000	0.001	-0.07	0.95
PI × step	0.001	0.0003	2.66	**0.01**
RT × step	0.001	0.001	1.36	0.17
SN2 × step	0.001	0.000	2.66	**0.01**
**Green algae**				
(Intercept)	0.002	0.001	1.45	0.15
PC	0.003	0.001	2.16	0.03

**Dark respiration**				
All specimens				
	Est.	S.E.	*t*	*P*
Lobaria				
(Intercept)	-0.004	0.002	-2.10	0.04
LA	0.006	0.008	0.78	0.43
PE	0.008	0.007	1.11	0.27
PI	0.008	0.003	2.93	**0.003**
RT	0.0003	0.006	0.05	0.96
SN2	0.015	0.004	3.53	**0.0004**
step	-0.002	0.000	-4.12	< **0.0001**
LA × step	-0.0005	0.002	-0.30	0.76
PE × step	0.0005	0.001	0.35	0.73
PI × step	-0.001	0.001	-1.13	0.26
RT × step	0.002	0.001	1.46	0.14
SN2 × step	-0.003	0.001	-3.02	**0.003**
**Green algae**				
(Intercept)	0.006	0.003	2.38	0.02
PC	-0.008	0.003	-3.12	**0.002**

S. reticulata (SR)	-0.002	0.003	-0.91	0.36
humidity	-0.0001	0.000	-4.57	<**0.0001**

Specimens with green algae *Symbiochloris reticulata*				
(Intercept)	-0.002	0.005	-0.50	0.61
PI	0.004	0.005	0.87	0.38
SN2	0.009	0.006	1.69	0.09
step	0.000	0.001	-0.02	0.99
PI × step	-0.001	0.001	-0.98	0.33
SN2 × step	-0.002	0.001	-2.14	**0.03**

Specimens with green algae *Parachloroidium*				
(Intercept)	0.008	0.006	1.31	0.19
LA	0.008	0.005	1.66	0.10
RT	0.005	0.003	1.62	0.11
humidity	-0.000	0.0001	-3.06	**0.002**

L. isidiosa with green algae *Parachloroidium*				
(Intercept)	0.008	0.007	1.15	0.25
Nostoc clade 5 (N5)	0.006	0.003	1.96	0.05
humidity	-0.000	0.0001	-3.14	**0.002**

SR	0.001	0.001	0.55	0.58
step	0.001	0.000	6.87	< **0.0001**

Specimens with green algae *Symbiochloris reticulata*				
(Intercept)	-0.004	0.002	-1.94	0.05
PI	0.004	0.002	2.64	**0.01**
SN2	0.003	0.002	1.76	0.08
humidity	0.0001	0.00001	4.96	< **0.0001**

Specimens with green algae *Parachloroidium*				
(Intercept)	0.005	0.001	4.54	< **0.0001**
LA	0.0003	0.002	0.14	0.89
RT	0.004	0.001	2.65	**0.01**
step	0.001	0.0002	2.16	0.03

L. isidiosa with green algae *Parachloroidium*				
(Intercept)	0.002	0.003	0.75	0.46
N5	0.001	0.001	0.94	0.35
humidity	0.0001	0.00	1.88	0.06

SR	-0.004	0.003	-1.47	0.14
step	-0.002	0.0002	-9.57	< **0.0001**

Specimens with green algae *Symbiochloris reticulata*				
(Intercept)	0.009	0.003	3.25	**0.001**
PI	-0.004	0.003	-1.72	0.09
SN2	-0.006	0.003	-2.10	0.04
step	-0.003	0.0002	-11.3	< **0.0001**

Specimens with green algae *Parachloroidium*				
(Intercept)	0.011	0.005	2.00	0.05
LA	0.005	0.005	1.12	0.27
RT	0.010	0.003	3.21	**0.001**
humidity	-0.0003	0.0001	-4.53	< **0.0001**

L. isidiosa with green algae *Parachloroidium*				
(Intercept)	0.012	0.006	2.01	0.05
N5	0.005	0.003	2.03	0.04
humidity	0.000	0.0001	-4.90	< **0.0001**

Table S7. Summary statistics for CO_2 exchange rate of dried *Lobaria* specimens which were re-activated when exposed to light and progressively increased relative humidity, shown as % of their original response to liquid water (NP%, GP% and *R*%).

		**Net photosynthetic rate (NP%)**					**Gross photosynthetic rate (GP%)**					**Dark respiration (*R*%)**				
		min	max	mean	S.E.	n	min	max	mean	S.E.	n	min	max	mean	S.E.	n
	Lobaria						*Lobaria*					*Lobaria*				
Chlorolichen	*L. pindarensis*	0.00	13.7	1.30	0.09	289	0.00	13.6	1.25	0.07	457	0.08	72.3	6.12	0.45	400
	L. perelegans	0.15	0.68	0.36	0.08	6	0.04	0.98	0.33	0.07	15	2.29	5.09	4.07	0.27	15
	Lobaria sp. 2	0.01	1.69	0.45	0.06	44	0.02	5.45	0.82	0.10	85	0.08	22.6	5.01	0.69	80
Chloro-cyanolichen	*L. isidiosa*	0.00	43.1	6.61	0.60	159	0.00	29.7	3.92	0.31	254	0.05	72.2	11.0	0.89	
	L. retigera	-14.6	33.6	5.32	1.91	33	0.07	14.4	2.59	0.41	49	0.15	31.8	9.60	1.52	36
	L. latilobulata	0.52	14.4	7.07	1.12	13	0.40	13.9	5.26	1.02	19	0.75	19.5	8.37	1.50	18
	Green algae						Green algae					Green algae				
	Parachloroidium	-14.6	43.1	6.6	0.61	187	0.003	29.7	3.87	0.29	282	0.052	72.2	11.6	0.83	265
	S. reticulata	0.003	13.7	1.17	0.08	339	0	13.6	1.16	0.06	557	0.076	72.3	5.88	0.38	495
	other	0	10.6	4.47	0.79	18	0.02	11.9	3.28	0.48	40	0.19	9.70	3.29	0.49	33
	Nostoc						*Nostoc*					*Nostoc*				
	Nostoc clade 4	0.16	33.6	6.53	0.85	79	0.07	20.5	3.20	0.35	123	0.05	72.2	10.8	117	1
	Nostoc clade 5	-14.6	43.1	6.46	0.78	118	0.00	29.7	4.08	0.39	176	0.15	38.3	10.6	163	1
	other *Nostoc*	0.00	10.4	4.98	1.32	8	0.02	11.9	4.82	0.72	23	0.19	32.4	10.4	18.0	3

Table S8. Dynamic linear model for CO_2 exchange rate of dried thalli which were re-activated when exposed to light and progressively increased relative humidity, shown as % of their original response to liquid water (NP%, GP% and *R*%). The linear time series is expressed according to either progressively increasing humidity conditions alone (humidity) or by the eight steps of the experiment (step). The chlorolichens were *L. pindarensis* (PI), *L. perelegans* (PE) and *Lobaria* sp. 2. (SN2). The chloro-cyanolichens were *L. isidiosa*, *L. latilobulata* (LA) and *L. retigera* (RT).

**Net photosynthetic rate (NP%)**				
All specimens				
	Est.	S.E.	*t*	*P*
Lobaria				
(Intercept)	6.67	0.82	8.10	< **0.0001**
L. latilobulata (LA)	3.17	2.63	1.20	0.23
L. perelegans (PE)	-6.19	3.39	-1.83	0.07
L. pindarensis (PI)	-5.23	1.02	-5.10	< **0.0001**
L. retigera (RT)	-9.87	1.98	-4.99	< **0.0001**
Lobaria sp. 2 (SN2)	-5.96	1.81	-3.30	**0.001**
step	-0.01	0.18	-0.08	0.94
LA × step	-0.80	0.64	-1.25	0.21
PE × step	-0.02	0.77	-0.03	0.98
PI × step	-0.02	0.22	-0.11	0.92
RT × step	2.13	0.43	4.94	< **0.0001**
SN2 × step	-0.06	0.44	-0.14	0.89

**Gross photosynthetic rate (GP%)**				
All specimens				
	Est.	S.E.	*t*	*P*
Lobaria				
(Intercept)	3.49	0.45	7.68	< **0.0001**
LA	-2.40	1.48	-1.62	0.11
PE	-3.46	2.25	-1.53	0.13
PI	-2.99	0.56	-5.32	< **0.0001**
RT	-1.39	1.10	-1.26	0.21
SN2	-3.51	0.90	-3.90	**0.0001**
step	0.09	0.09	1.04	0.30
LA × step	0.91	0.31	2.95	**0.003**
PE × step	-0.04	0.40	-0.09	0.93
PI × step	0.07	0.11	0.60	0.55
RT × step	0.02	0.22	0.07	0.95
SN2 × step	0.08	0.17	0.48	0.63

**Dark respiration (*R*%)**				
All specimens				
	Est.	S.E.	*t*	*P*
Lobaria				
(Intercept)	8.23	1.61	5.10	< **0.0001**
LA	-10.1	5.62	-1.81	0.07
PE	-4.28	8.44	-0.51	0.61
PI	-9.15	2.01	-4.56	< **0.0001**
RT	6.66	4.96	1.34	0.18
SN2	-12.7	3.47	-3.66	**0.0003**
step	0.60	0.32	1.89	0.06
LA × step	1.69	1.12	1.51	0.13
PE × step	-0.58	1.44	-0.40	0.69
PI × step	0.91	0.39	2.32	0.02
RT × step	-1.68	0.94	-1.78	0.08
SN2 × step	1.27	0.65	1.96	0.05

**Green algae**				
(Intercept)	9.54	4.35	2.19	0.03
Parachloroidium (PC)	-7.50	4.59	-1.63	0.10
S. reticulata (SR)	-8.18	4.47	-1.83	0.07
humidity	-0.06	0.05	-1.21	0.23
PC × humidity	0.12	0.05	2.20	0.03
SR × humidity	0.06	0.05	1.13	0.26

Specimens with green algae *Symbiochloris reticulata*				
(Intercept)	0.57	0.61	0.93	0.35
PI	0.98	0.58	1.70	0.09
SN2	0.13	0.61	0.21	0.83
humidity	0.00	0.00	-0.91	0.36

Specimens with green algae *Parachloroidium*				
(Intercept)	6.46	1.46	4.44	**< 0.0001**
LA	3.38	4.37	0.77	0.44
RT	-9.66	3.31	-2.92	**0.004**
step	0.11	0.32	0.33	0.74
LA × step	-0.92	1.07	-0.86	0.39
RT × step	2.01	0.72	2.78	**0.01**

**Green algae**				
(Intercept)	1.78	0.68	2.62	**0.01**
PC	0.53	0.51	1.04	0.30
SR	-2.17	0.50	-4.35	**< 0.0001**
humidity	0.02	0.01	3.12	**0.002**

Specimens with green algae *Symbiochloris reticulata*				
(Intercept)	-0.73	0.48	-1.54	0.12
PI	0.96	0.38	2.53	0.012
SN2	0.53	0.40	1.30	0.19
humidity	0.01	0.00	3.58	**0.0004**

Specimens with green algae *Parachloroidium*				
(Intercept)	0.60	1.58	0.38	0.70
LA	1.46	1.15	1.27	0.21
RT	-1.41	0.76	-1.86	0.06
humidity	0.04	0.02	2.23	0.03

**Green algae**				
(Intercept)	-9.86	2.60	-3.79	**0.0002**
PC	9.07	1.86	4.88	**< 0.0001**
SR	3.38	1.81	1.87	0.06
humidity	0.14	0.02	6.83	**< 0.0001**

Specimens with green algae *Symbiochloris reticulata*				
(Intercept)	-4.51	2.23	-2.02	0.04
PI	3.51	2.07	1.69	0.09
SN2	1.73	2.21	0.78	0.44
step	1.53	0.17	9.28	**< 0.0001**

Specimens with green algae *Parachloroidium*				
(Intercept)	-3.27	4.37	-0.75	0.45
LA	-3.15	3.25	-0.97	0.33
RT	-3.37	2.39	-1.41	0.16
humidity	0.18	0.05	3.63	**0.0003**

L. isidiosa with green algae *Parachloroidium*				
(Intercept)	2.69	2.85	0.95	0.35
Nostoc clade 5 (N5)	1.31	1.51	0.87	0.39
humidity	0.04	0.03	1.26	0.21

L. isidiosa with green algae *Parachloroidium*				
(Intercept)	0.98	2.07	0.47	0.64
N5	0.73	0.78	0.94	0.35
humidity	0.03	0.02	1.25	0.21

L. isidiosa with green algae *Parachloroidium*				
(Intercept)	2.80	3.54	0.79	0.43
N5	6.59	4.91	1.34	0.18
step	2.35	0.75	3.13	**0.002**
N5 × step	-1.91	1.00	-1.91	0.06
