# Supplementary material for: Acquisition of green algal photobionts enables both chlorolichens and chloro-cyanolichens to activate photosynthesis at low humidity without liquid water
Source: AoB Plants. 2024 Apr 29;16(3):plae025. doi: 10.1093/aobpla/plae025 (PMC11102867; doi:10.1093/aobpla/plae025)
Supplement: plae025_suppl_Supplementary_Information_S3 [file plae025_suppl_supplementary_information_s3.pdf]

Acquisition of green algal photobionts enables both chlorolichens and chloro-cyanolichens to activate photosynthesis at low humidity without liquid water

Fiona Ruth Worthy, Douglas Allen Shaefer, Dhanushka Wanasinghe, Jian Chu Xu, Li Song Wang and Xin Yu Wang

Supporting Information 3: Methods

Methods S1. Lichen dry mass

Comparison of thallus dry mass *M* after drying over $CaCl_2$ for 1 day or approximately 2 weeks, indicated that additional duration of drying made negligible difference to *M* of the chlorolichens, but that the chloro-cyanolichens did have slight reductions in *M* over time (Supporting Information 3: Fig. S2). Because only subsets of the specimens were used in the final humidity-control experiment, it is not valid to compare mass at 2 weeks with that after > 4 months drying over silica gel. We did not repeatedly measure *M* during those 4 months, in order to avoid unnecessarily exposing specimens to room humidity or light prior to the intended time point of measuring humidity and light response in the final experiment. Thallus surface area *S* and *M* were used to calculate Specific Thallus Mass STM (g cm^{-2}):

$$\text{STM} = M / S \qquad \text{(S1)}$$

These values are not truly comparable between the original specimens and their subsets, but do indicate that it is unlikely that thalli absorbed moisture between these time intervals (Supporting Information Fig. S2).

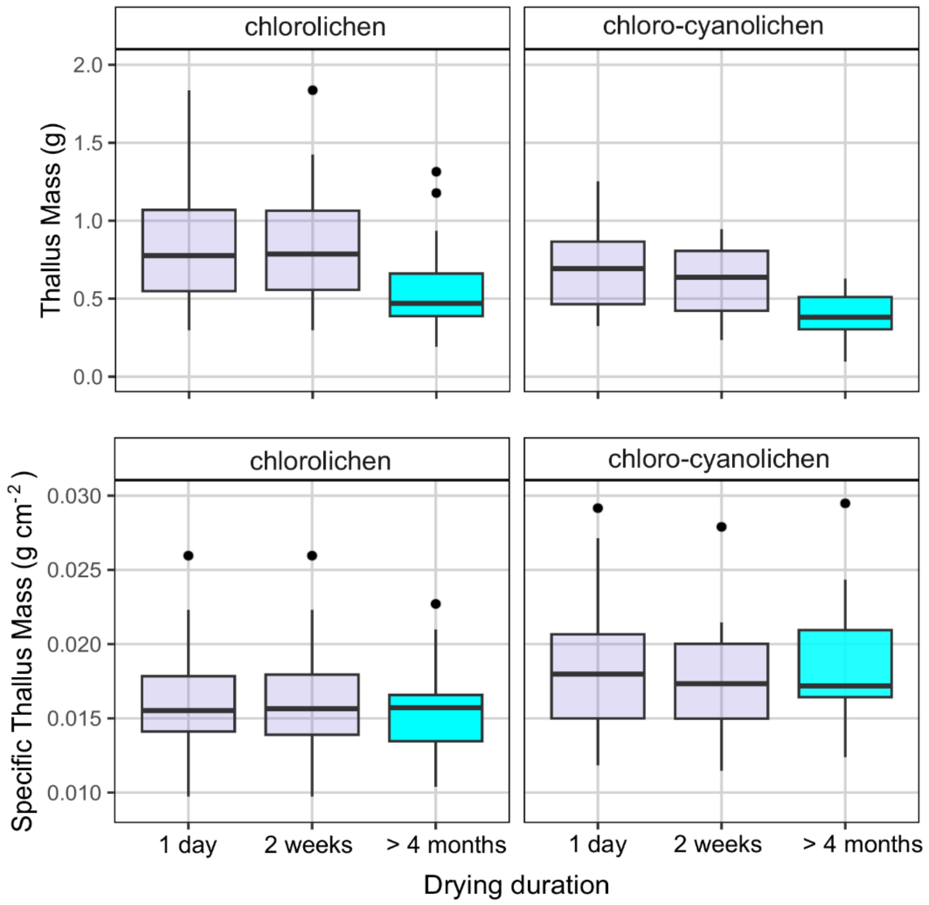

Figure S2. Thallus dry mass (g) and Specific Thallus Mass (g cm^{-2}) of chlorolichen and chloro-cyanolichen specimens. Initial thallus dry mass and thallus area were measured after specimens had been dried over $CaCl_2$ for 1 day. After ≈ 2 weeks, thallus dry mass and thallus area were measured again prior to adding water and measuring CO_2 exchange rates. Thalli were then air dried, desiccated over $CaCl_2$ then stored over silica gel. After > 4 months, a subset of the original specimen was measured, then used for the humidity response experiment (turquoise boxes). Thus, only the change in mass between 1 day and 2 weeks represents reductions in thallus water content.

Methods S2. LICOR experiment - humidity control

A schematic diagram of the photosynthesis experiment set-up is shown in Supporting Information 3: Fig. S3. Photographs of the LICOR set-up and data output are shown in Supporting Information 3: Fig. S4. Great care was taken to avoid condensation, including by keeping humidity-control jars and lichen chambers at the same temperature (15°C). Within the lichen chambers, specimens were suspended above netting, thus avoiding direct contact with the base of the chamber. After each 24-hour humidity input, if any specimens had an odour indicative of decay, they were excluded from further use in the experiment.

After any session with K_2SO_4, before returning to any lower-humidity condition, the experimental loop was attached to a jar containing $CaCl_2$ for at least 30 minutes, in order to ensure no humid air was retained within the LICOR.

Before each experiment, thallus area was checked using the digital Leafscan mobile application software (version 2.1.1). Before each day's humidity trials, each specimen was weighed. Thus, they were briefly exposed to ambient RH while being measured. Therefore, room humidity and temperature were recorded throughout the experiment, and it was checked that room RH was not related to specimens with higher CO_2 exchange rates.

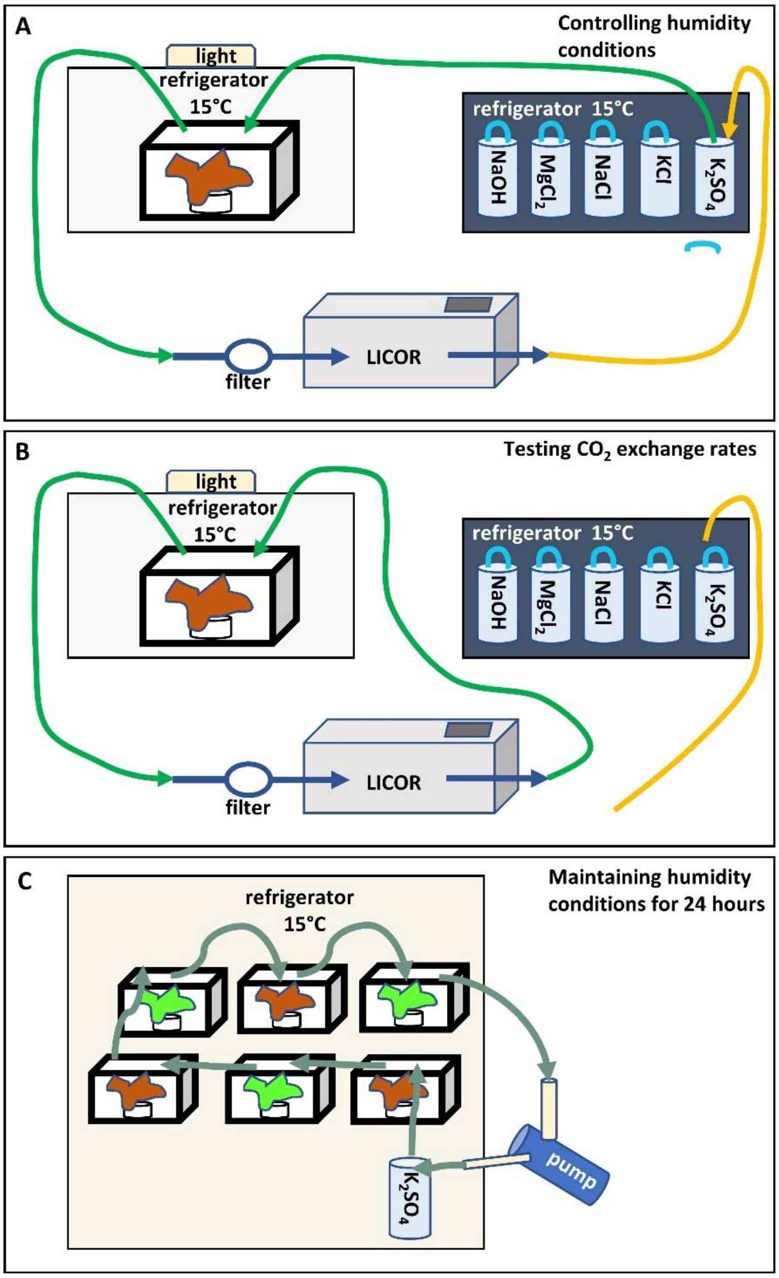

Figure S3. Schematic diagram of photosynthesis experiment set-up. Air circulating through tubes (arrows show direction of flow) following a circuit. Different sets of tubes are shown in different colours to indicate which tubes are attached or detached in each panel. (A) Air passes over one of four humidity-control jars (either $MgCl_2$, NaCl, KCl or K_2SO_4), then a sealed lichen-chamber, then finally back to the LICOR. Air was pumped by the LICOR, with no additional external pump required. (B) Humidity-control jars are removed and LICOR is

used to record CO_2 levels. (C) Lichens kept in the dark in individual chambers for 24 hours, while maintained at constant temperature and humidity. Air was pumped over either KCl or K_2SO_4 saturated solution, then through the lichen chambers.

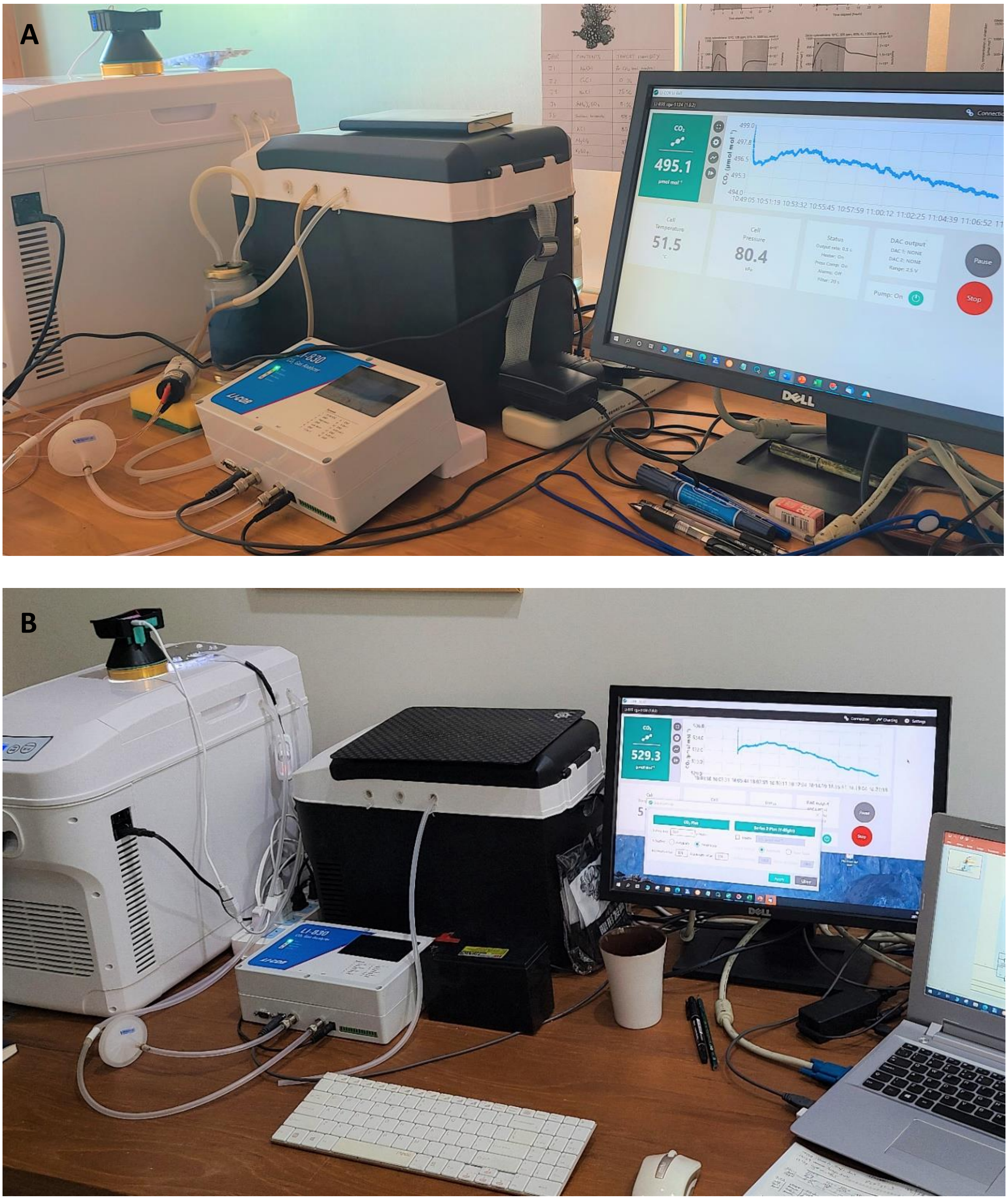

Figure S4. Photographs of LICOR set-up and outputs for: (A) *Lobaria retigera* at 33% RH, (B) *L. retigera* after 24 hours at 98% RH. The external pump and humidity-control jars have been detached from the circuit prior to recording CO_2 levels.

Methods S3. LICOR experiment - Minimising leaks from the circuit

In order to measure CO_2 exchange at low relative humidity (RH), our equipment needed to be highly sensitive, leaks prevented and ambient room CO_2 levels kept near to the experimental levels, to minimise partial pressure differences. Many different designs and volumes of lichen chambers and tubing were pre-tested, before selecting Sistema Brilliance 380 mL transparent sealed chambers (SIS1007675) (Supporting Information Fig. S5A), with 6 mm drilled holes in the lid (Supporting Information 3: Fig. S5B), connected with rubber washers (Supporting Information 3: Fig. S5D) and Runze Fluid panel mount bulk-head fittings (DC-032-1/4-28-032P) (Supporting Information 3: Fig. S5C,E) to silicone tubes (96402-16) (Supporting Information 3: Fig. S5I).

Multiple aspects of the chamber design were critical in minimising leaks from the chamber itself. The Sistema chambers are manufactured with a rubber seal between the lid and the body of the container. Pressure can be applied with two side clamps (Supporting Information 3: Fig. S5E). In this experiment, white Teflon tape was placed over a hole beneath the clamps in order to further improve the seal (Supporting Information 3: Fig. S5F – H). The two holes drilled in the lid to allow air flow in and out of the chamber (Supporting Information 3: Fig. S5B) were as close as possible in diameter to that of the bulk head fittings. A thin rubber seal was placed above and beneath each hole (Supporting Information 3: Fig. S5D), then bulk head fittings were screwed on. Tubing was attached to the bulk head fittings (Supporting Information 3: Fig. S5I). Each chamber was tested for leaks before the start of each week of experiments. If leaks were detected, then each of the above aspects of the chamber were checked and testing repeated (example displayed in Supporting Information 3: Fig. S6).

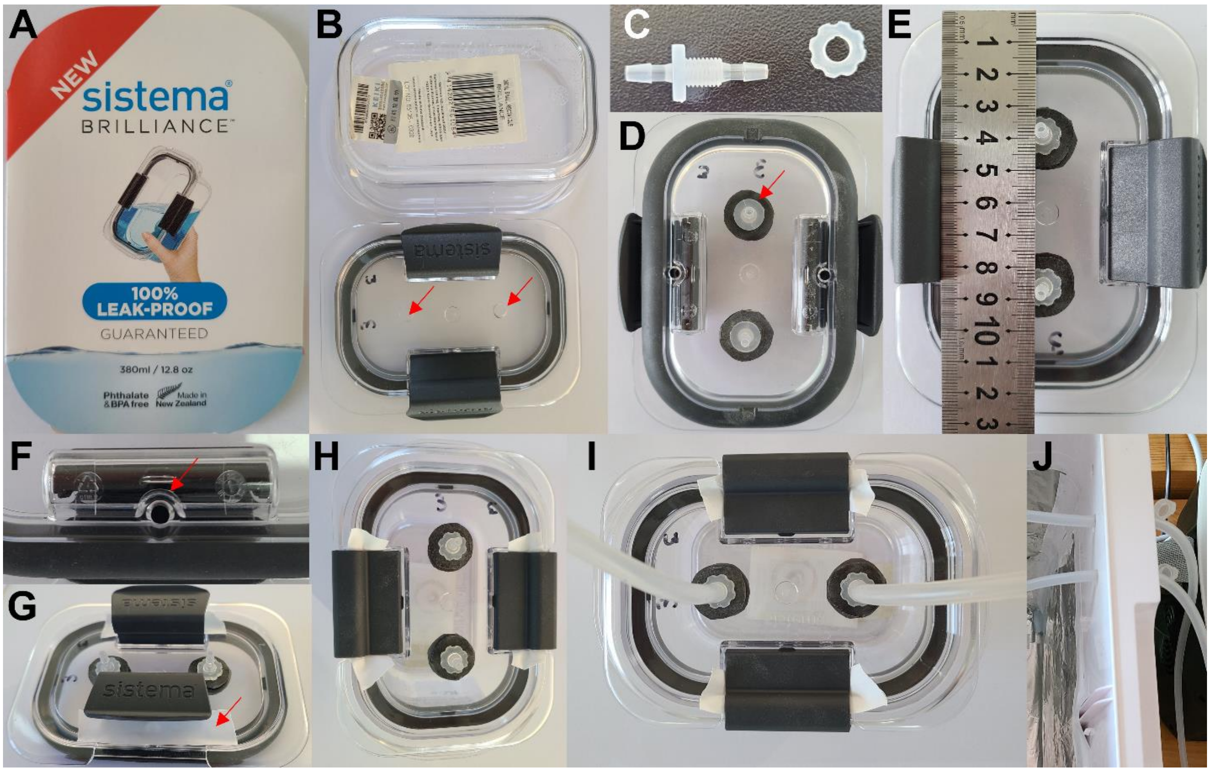

Figure S5. Construction of the lichen chambers used in the LICOR experiment. (A) Manufacturer's description of 380 mL Sistema Brilliance container. (B) Base and lid of lichen chamber. Red arrows indicate 6 mm diameter holes drilled through the lid. (C) Bulk head fittings. (D) Underside of lid after fitting rubber seals (red arrow) and bulk head fittings. Black oval is the manufacturer's rubber seal. (E) Top view of lid after fitting rubber seals and bulk head fittings. (F) Hole beneath clamp (red arrow). (G) White Teflon tape (red arrow) placed beneath the clamp. (H) Top view of completed lichen chamber. (I) Tubing attached to lichen chamber. (J) Tubing passing through the wall of the refrigeration unit.

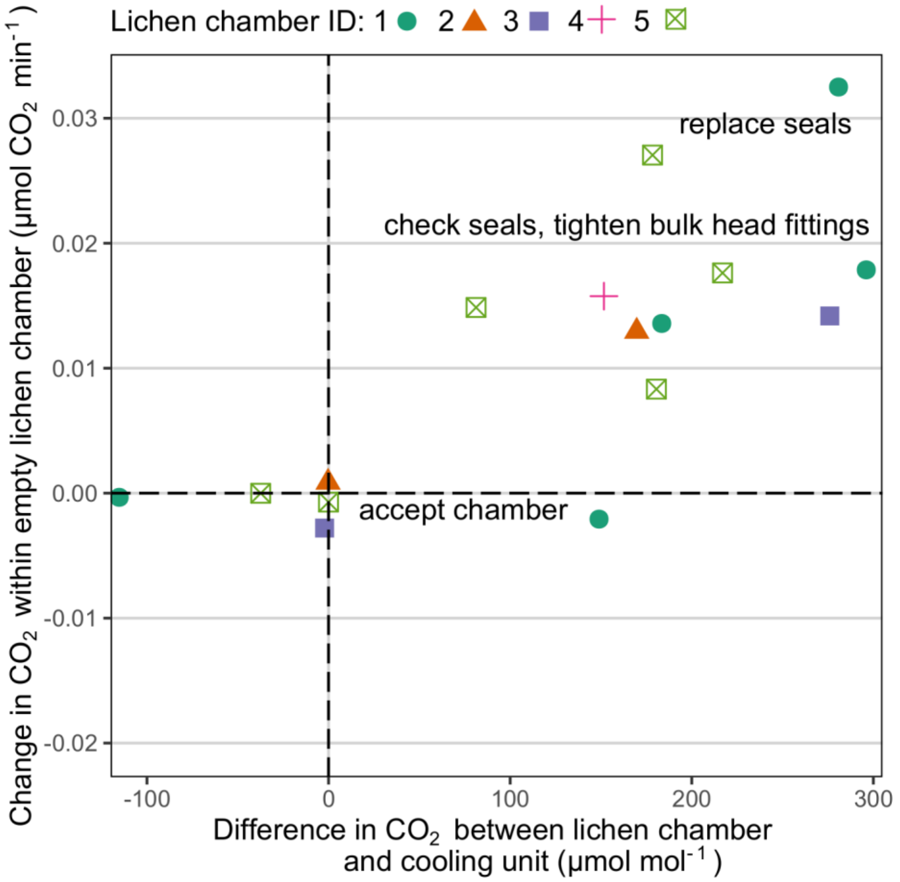

Figure S6. Examples of checking for CO_2 changes when the circuit included only empty lichen chambers, new tubing and the LICOR. Any such changes would be considered leaks from the circuit. Increasing the difference in CO_2 between the lichen chamber and the cooling unit aided the detection of leaks. On this day, replacing seals and tightening bulk head fittings minimised leaks for chambers 1, 2, 3 and 5. When both lichen chamber and cooling unit had the same starting CO_2 µmol mol^{-1}, the total circuit leak rate was < 0.003 µmol CO_2 min^{-1}, and these chambers were accepted for use in the experiment. Chamber 4 was not used in that day's photosynthesis experiments.

As LICOR equipment ages, leaks could potentially increase from within the LICOR itself. This experiment was conducted with a newly purchased LICOR 830. Tubing is also more likely to leak with age. The leak rate will increase in proportion to the length of the tubing. The tubing was replaced before the start of the experiment. With high room CO_2, the leak rate from a circuit with only the LICOR and the 103 cm of tubing used in the experiment was 0.001 µmol CO_2 min^{-1}: thus 1.07×10^{-5} µmol CO_2 min^{-1} cm^{-1}. Leaks between sections of tubing were reduced by having the minimum number of joins consistent with circuit design. This required drilling holes through the side of the refrigerator through which intact tubing could pass (Supporting Information 3: Fig. S5J).

Attachment of humidity-control jars caused major leaks from the circuit. Therefore, jars were always removed from the circuit prior to recording changes in CO_2 concentration.

Despite the above steps, if the difference in CO_2 concentration between the outside and inside of the chamber exceeded 150 µmol mol^{-1}, there was a substantial risk that the leak rate might exceed the CO_2 exchange rate of the lichen (compare Supporting Information 3: Fig. S6 with Supporting Information 1: Fig. S11). Therefore, the lab was kept well ventilated. Prior to each stage at which a lichen chamber was attached to the circuit, room CO_2 levels were brought as close to ≈ 500 µmol mol^{-1} as reasonably practicable. As far as possible, only one observer was present in the room, in order to reduce the extent to which breathing would elevate room CO_2 concentration. The lichen chamber was placed inside a small refrigerator (SAST PD-28); providing a double level of sealing, along with temperature control. CO_2 levels were brought to ≈ 500 µmol mol^{-1} within this refrigerator prior to sealing the lichen chamber.

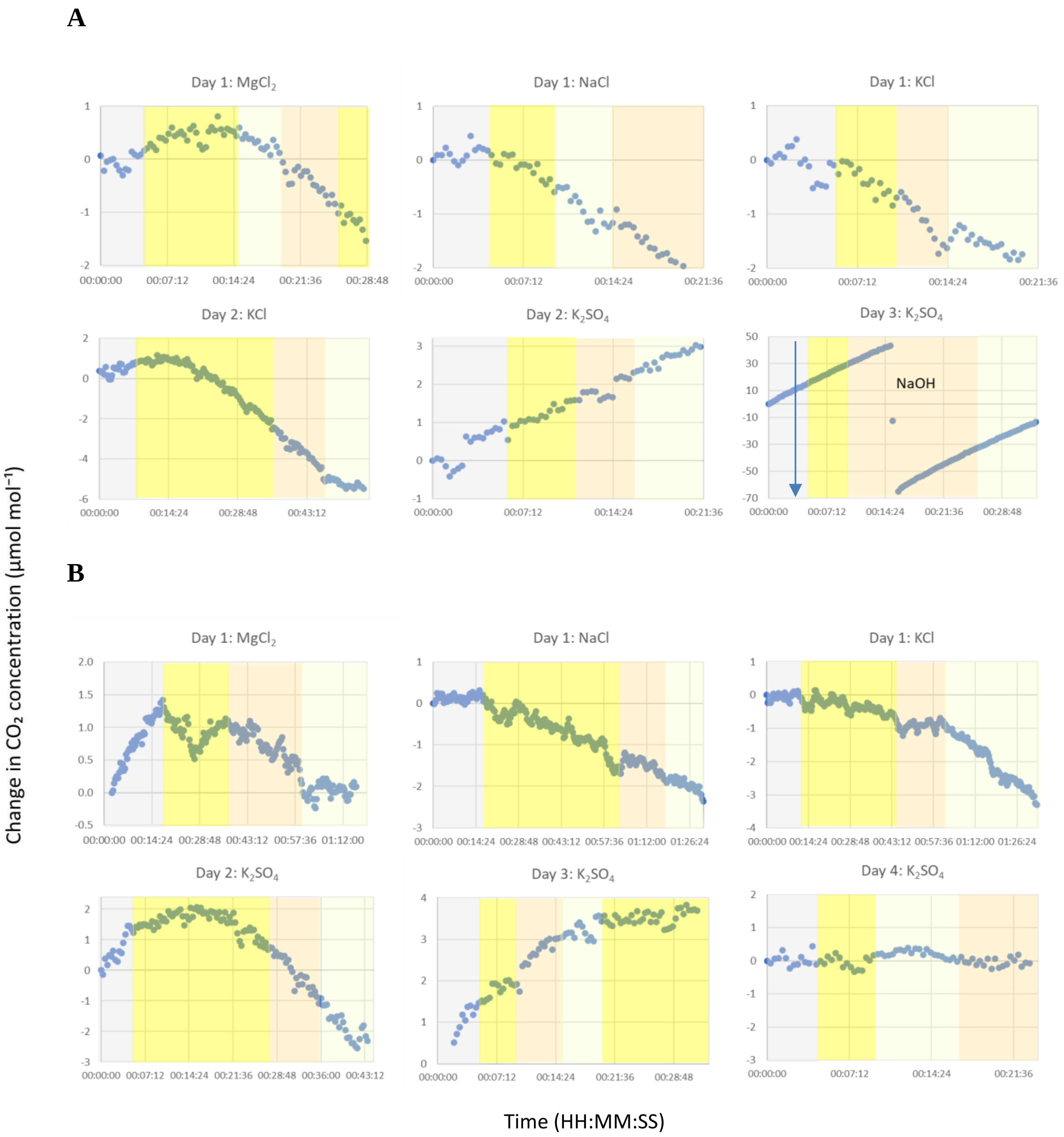

Figure S7. Examples of raw LICOR data for a desiccated specimen of (A) *Lobaria pindarensis* with photobiont *Symbiochloris reticulata* and (B) *Lobaria isidiosa* with photobionts *Nostoc* 5 and *Parachloroidium* sp. clade 1. Light conditions are dark (grey), 300 µmol m^{-2} s^{-1} (bright yellow), 170 µmol m^{-2} s^{-1} (orange) and 30 µmol m^{-2} s^{-1} (pale yellow).

Specimens were exposed to sequentially increasing humidity conditions (33%, 76%, 86%, and 98%) obtained by circulating air over humidity-control chambers containing the following solutions: $MgCl_2$, NaCl, KCl and K_2SO_4.

Methods S4. LICOR experiment - CO_2 flux rates

The flow rate W through the LICOR was 0.73 ± 0.0014 L min^{-1} (mean ± S.E.). The volume of the circuit was composed of the lichen chamber V_{ch} (0.38 L), tubing V_{tu} (≈ 0.02 L) and LICOR V_{cor} (≈ 0.0145 L). Addition of a lichen specimen reduced the system volume. The volume of each lichen specimen V_{sp} was estimated from its thallus surface area S and thallus thickness H. Thallus thickness was estimated as 3 mm. Thallus surface area was directly measured by scanning each specimen with the digital Leafscan 2.1.1 mobile application software. Total system volume V was calculated as:

$$V_{sp} = S \times H \qquad \text{(S2)}$$

$$V = (V_{ch} + V_{tu} + V_{cor}) - V_{sp} \qquad \text{(S3)}$$

The turnover time of gas U within the system was calculated as:

$$U = V / W \qquad \text{(S4)}$$

The total system volume would be well mixed and would have turned over in 33 seconds.

The ideal gas law was used to calculate the number of moles of gas n:

$$n = PV / rT \qquad \text{(S5)}$$

Where P was pressure, measured as 80,000 Pascals. Total system volume was V (converted to m^3), r was the gas constant 8.31 J K^{-1} mol^{-1} and T was room temperature (K).

The CO_2 flux rate F (μmol min^{-1}) at each stage of the experiment was:

$$F = - (\Delta C \times n) / t \quad \text{(S6)}$$

Where t was time (min) and ΔC was change in CO_2 concentration in the chamber (µmol mol^{-1}).

Examples of such calculated CO_2 flux rates are displayed in Fig. 7.

The above estimates of V_{sp} are imprecise as H will vary even across a single thallus and both S and H would tend to increase as the thallus absorbed water vapour. Such changes could not be directly measured by water displacement because water absorption would alter $V_{sp.}$ For comparisons between specimens and over time, calculated values for V are unlikely to be more precise than $V_{ch.}$ Attempted 3D scanning with the applications SCANN3D version 3.1.0-10024 and ON 3D-CameraMeasure did not produce repeatable measures of $V_{sp.}$

Therefore, for the purposes of the main manuscript, respiratory and photosynthetic rates were calculated based on:

$$V = V_{ch} + V_{tu} + V_{cor} \quad \text{(S7)}$$

Where M represents lichen thallus dry mass (g). While in the dark, lichen dark respiration rate R (µmol CO_2 min^{-1} g^{-1}) was calculated as:

$$R = - ((\Delta C \times n) / t) / M) \quad \text{(S8)}$$

While exposed to light, lichen net photosynthetic rate (NP) (µmol CO_2 min^{-1} g^{-1}) was calculated as:

$$\text{NP} = - ((\Delta C \times n) / t) / M) \quad \text{(S9)}$$
